# A Symbol of Immortality: Evidence of Honey in Bronze Jars Found in a Paestum Shrine Dating to 530–510 BCE

**DOI:** 10.1021/jacs.5c04888

**Published:** 2025-07-30

**Authors:** Luciana da Costa Carvalho, Elisabete Pires, Kelly Domoney, Gabriel Zuchtriegel, James S. O. McCullagh

**Affiliations:** † Mass Spectrometry Research Facility, Department of Chemistry, 6396 University of Oxford, Oxford OX1 3TA, United Kingdom; ‡ School of Archaeology, University of Oxford, Oxford OX1 3TG, United Kingdom; § Ashmolean Museum, Beaumont Street, Oxford OX1 2PH, United Kingdom; ∥ 261090 Parco Archeologico di Pompei, Via Plinio 26, Pompei, NA 80045, Italy

## Abstract

This study re-examines a 2500-year-old residue found in bronze jars at an underground shrine in Paestum (Italy), previously identified as a wax/fat/resin mixture excluding honey from its composition. Our multianalytical approach detected lipids, saccharide decomposition products, hexose sugars, and major royal jelly proteins supporting the hypothesis that the jars once also contained honey/honeycombs. The research highlights the value of reinvestigating archeological residues in museums with advanced biomolecular techniques and offers a more specific method for detecting bee products in ancient contexts.

## Graphical abstract

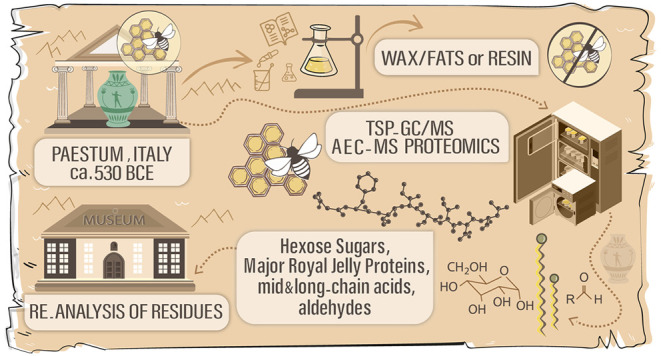

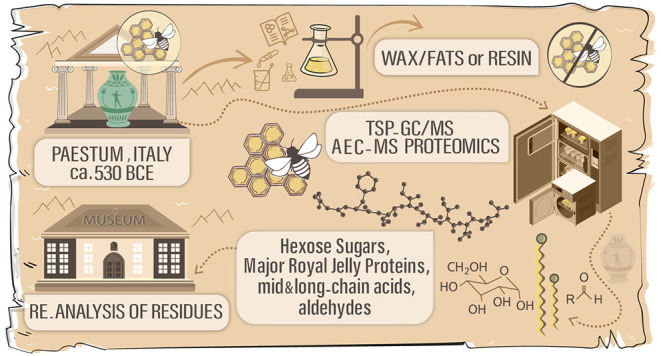

## Introduction

Honey was a pivotal substance in ancient societies. Historical accounts and images indicate that honey was used as an early sweetener in medicinal preparations,^1^ in rituals,^2^ and in cosmetics.^3^ In ancient Greek and Roman cultures, bees and honey held significant religious and symbolic importance. Honey was believed to nurture wisdom, with myths suggesting that Zeus himself was fed honey as a child.^4^ The identification of honey in archeological residues provides direct chemical evidence of bee product collection, exploitation, and processing, shedding light on early farming and subsistence strategies in different regions of the world.^5,6^

In archeological and historical contexts, identifying evidence of honey has traditionally relied on the identification of wax esters and long-chain alcohols—characteristics of beeswax—in lipid extracts from porous ceramics assumed to have been used for processing of honeycombs (hexagonal cells made of beeswax and filled with honey).^7–9^ More recently, carbohydrates have also been detected.^10^

In 1954, archeologists excavating in the sixth century BCE Greek settlement in Paestum (in southern Italy) found an underground shrine to an unknown deity (Figure 1A) containing bronze jars—six hydrae (e.g., Figure 1B) and two amphorae—surrounding an empty iron bed (Figure 1C). The jars contained a paste-like residue with a strong wax aroma (Figure 1D). Archaeologists reported the residue to have been originally a liquid or viscous liquid, as traces of it were found on the exterior of the vessels, which were originally sealed with cork disks.^11^ Their excavation report emphasized the sacredness of the shrine: the empty bed and the inaccessibility of the shrine signify that the deity was there. Moreover, the archeologists identified the original contents of the bronze jars as having been honey, “a symbol of immortality,”^11^ originally offered as honeycombs but of which only beeswax remained as the main element. However, three subsequent laboratory analyses of different samples of the residue excluded honey from its composition.

**Figure 1:**
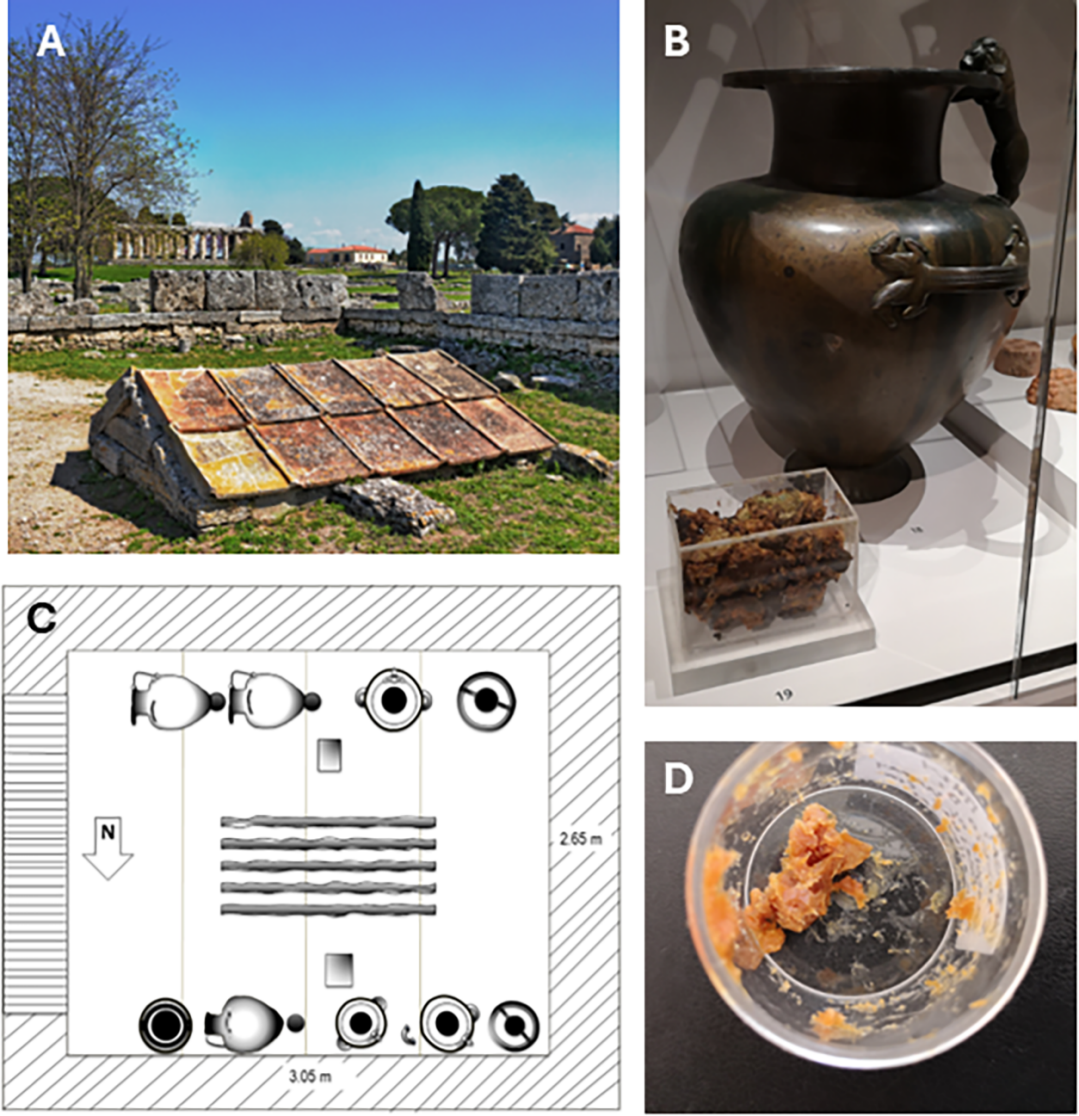
(A) Underground shrine in Paestum. (B) One of the hydrias on display alongside a Perspex box containing the residue at the Ashmolean Museum in 2019. (C) Graphic representation of the arrangement of the bronze jars inside the shrine, based on Sestieri 1956. (D) Sample from the core of the residue.

The first characterization of the residue was carried out a few years after excavation by a German laboratory at the request of London’s Bee Research Association.^12^ The residue was not soluble in water; when other solvents were used, the soluble fraction was identified as a fatty substance similar to wax.^12^ Plant and insect remains, fungi and pollen, found in the sediment leftover were considered to be contamination, and it was suggested that perhaps the sample submitted for analysis represented a wax layer added on top of the original content of the jars.^12^ In 1970, samples of the residue taken from the neck and bottom of one of the amphorae were analyzed by the *Istituto Centrale del Restauro* in Rome. Various solubility tests detected only saponifiable substances (i.e., fats, waxes or resins) in the samples and excluded the presence of sugars or proteins.^12^

The most recent analysis of the residue was conducted in 1983 by the Laboratory of the Rome Chamber of Commerce. Their report stated the sample was not soluble in water and did not contain sugary or starchy substances.^12^ The residue was soluble in ethyl ether and 99% saponifiable. Its fatty acid composition (identified by gas chromatography coupled with mass spectrometry) was 77.4% palmitic acid, 6.1% oleic acid, 5.2% stearic acid, 1.0% heptadecanoic acid, 1.0% linoleic acid + arachidic acid, 0.4% linoleic acid, and 6.5% unidentified substance.^12^ As palmitic acid is widespread in nature in the form of triglycerides, the report concluded that the residue once contained animal/vegetable fats and phospholipids.

In 2019, when the residue arrived at the Ashmolean Museum for display at the “Last Supper in Pompeii” exhibition, it provided a new opportunity to reinvestigate its biomolecular composition, taking advantage of recent advances in mass spectrometry instrumentation.

## Experimental Section

The Paestum residue arrived at the Ashmolean Museum in a nonhermetically sealed Perspex box as shown in Figure 1B. We do not have a detailed history of its collection or its storage conditions since excavation, only that it was displayed in a showcase at the Paestum Museum. To minimize the effects of postexcavation contamination and deterioration, our biomolecular characterization focused on a sample extracted from the core of the material, collected from a depth of 40 mm within the main body (Figure 1D). Due to the distinct visual differences observed between the surface and the core of the residue, microsamples—each equivalent to a few crystals—were also selectively taken from the black, green, and orange surface areas for comparative analysis. According to Sestieri,^12^ the residue’s surface discoloration likely resulted from chemical interactions between the residue and the bronze vessel over time. Samples were collected in the Conservation Department at the Ashmolean Museum using individual stainless-steel spatulas and placed in a sealed crystal box (core sample) or in individual Eppendorf Safe-Lock tubes (surface samples) for transport to the Chemistry Research Laboratory, where they were stored at ambient temperature (ca. 20 °C) until analysis. Despite being visually different, surface and core samples retained a paste-like consistency.

To aid data interpretation, in addition to samples of the residue, we also analyzed modern beeswax, honey, and honeycombs. To try to account for chemical variability due to environmental factors, we sourced honeycombs from two locations (in Italy and Greece) and subjected them to accelerated aging using heat in an attempt to simulate chemical changes that may occur overtime.

Given the chemical heterogeneity of biomarkers that could be targeted, we adopted a multianalytical approach that combined spectroscopy with high resolution chromatography coupled to mass spectrometry techniques. We used Fourier Transform Infrared Spectroscopy (FTIR) to obtain an overview of the chemical functions present and targeted thermally labile compounds by Gas Chromatography coupled to Quadrupole Time-Of-Flight Mass Spectrometry analysis using a thermal separation probe (TSP-GC/MS). This technique requires no derivatization prior to analysis,^13–15^ thus reducing sample processing biases, potential compound loss, or altered profiles. Hexose sugars and other water-soluble small molecules were targeted by Anion exchange Ion-Chromatography coupled to Mass Spectrometry (AEC-MS) using a method developed for metabolomics studies^16^ and proteins by Bottom-Up Proteomics.

Because of their small size, the analysis of the residue’s surface samples was restricted to TSP-GC/MS and X-ray Photoelectron Spectroscopy (XPS), the latter technique is used to investigate any potential interaction between the residue and its original metal container. Further experimental details are included in the Supporting Information S1.

## Results

### FTIR

Figure 2 shows the residue’s FTIR spectrum superimposed on the spectra of modern beeswax and modern honey. The residue and modern beeswax’s spectra overlap at 2916 and 2849 cm^–1^ corresponding to C–H vibrations of long aliphatic compounds, at 1463 cm^–1^ corresponding to C–CH alkane deformations and most bands in the fingerprint region (1350–900 cm^–1^) which arise from various skeletal vibrations.^17^ A crucial difference between these spectra is a strong band for C=O stretching vibrations which in beeswax appeared at 1736 cm^–1^ corresponding to esters but that in the residue, appeared at 1703 cm^–1^ corresponding to carboxylic acids. For honey, the C–H vibrations for long aliphatic compounds and the C=O vibrations for acids were weaker. Instead, honey’s spectrum was dominated by a broad band centered around 3300 cm^–1^ corresponding to OH^−^ stretching vibrations^17^ resulting from its higher water content than beeswax’s and a medium doublet band at around 1028 cm^–1^ corresponding to C–O in C–OH group or C–C stretching vibrations for carbohydrates.^18^

**Figure 2:**
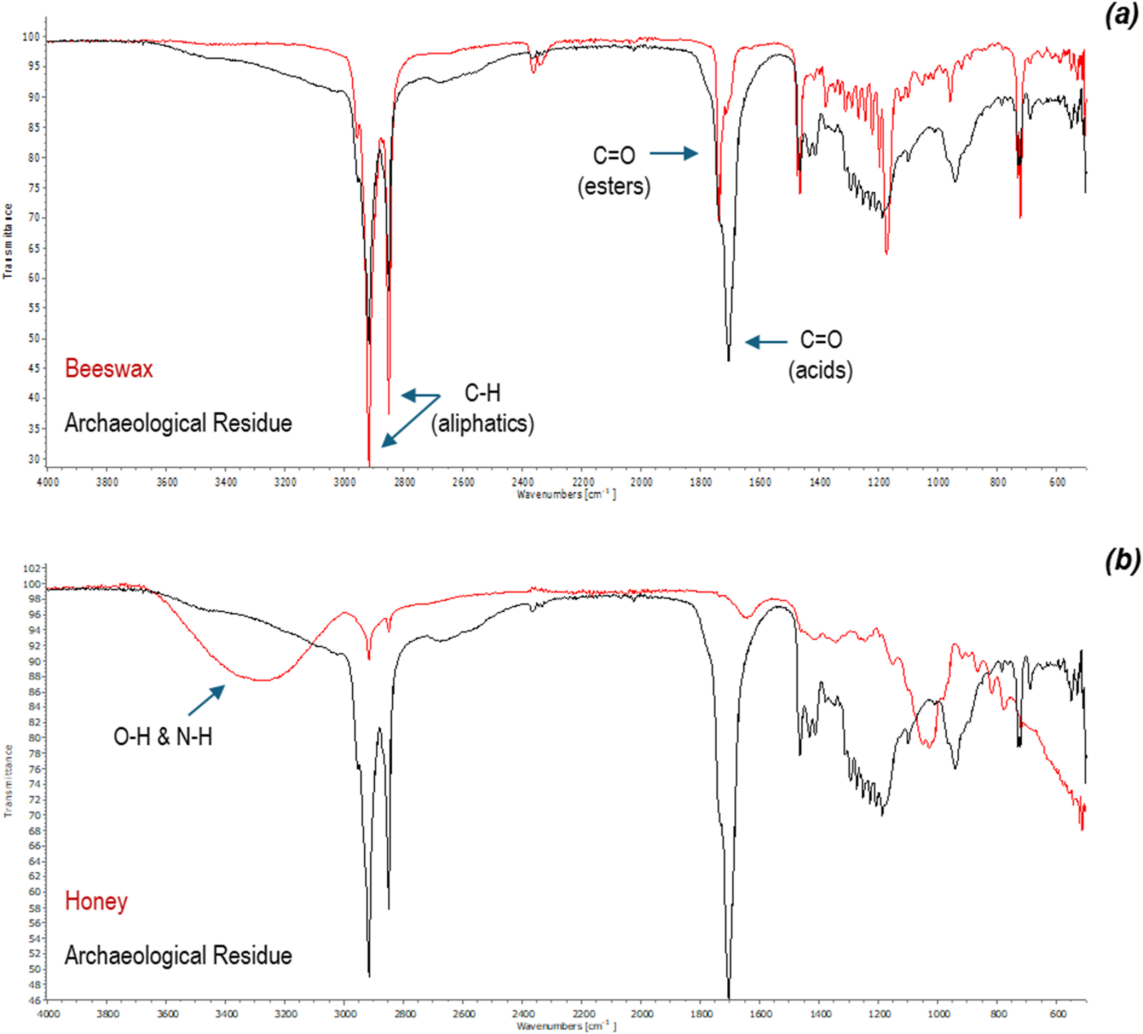
FTIR Spectrum of the core sample of the archeological residue superimposed on beeswax (a) and honey’s (b).

The spectra obtained for the fresh and aged honeycombs were practically identical (Supporting Information S2, Figures S1 and S2). Their main peaks were a broad band centered around 3340 cm^–1^ corresponding to OH^−^ stretching vibrations, strong peaks at 2914 and 2847 cm^–1^ for C–H vibrations in aliphatic compounds, medium/strong peak at 1735 cm^–1^ corresponding to C=O ester vibrations, and a shoulder peak at around 1703 cm^–1^ for C=O carboxylic acid vibrations.

### TSP-GC/MS

The total ion chromatograms (TICs) obtained from analysis of the residue core sample, beeswax, honey, and fresh and aged honeycombs are shown in Figure 3. It was observed that after analysis, the microvial containing beeswax was visually “clean,” indicating that most of the compounds present were efficiently desorbed at 300 °C without thermal resistance. In contrast, the vials used for the archeological residue, honey, and honeycomb samples all displayed a dark brown residual film with a burnt sugar odor, suggesting the presence of thermally labile saccharide components that underwent “caramelization” or charring in response to the raised temperature.

**Figure 3:**
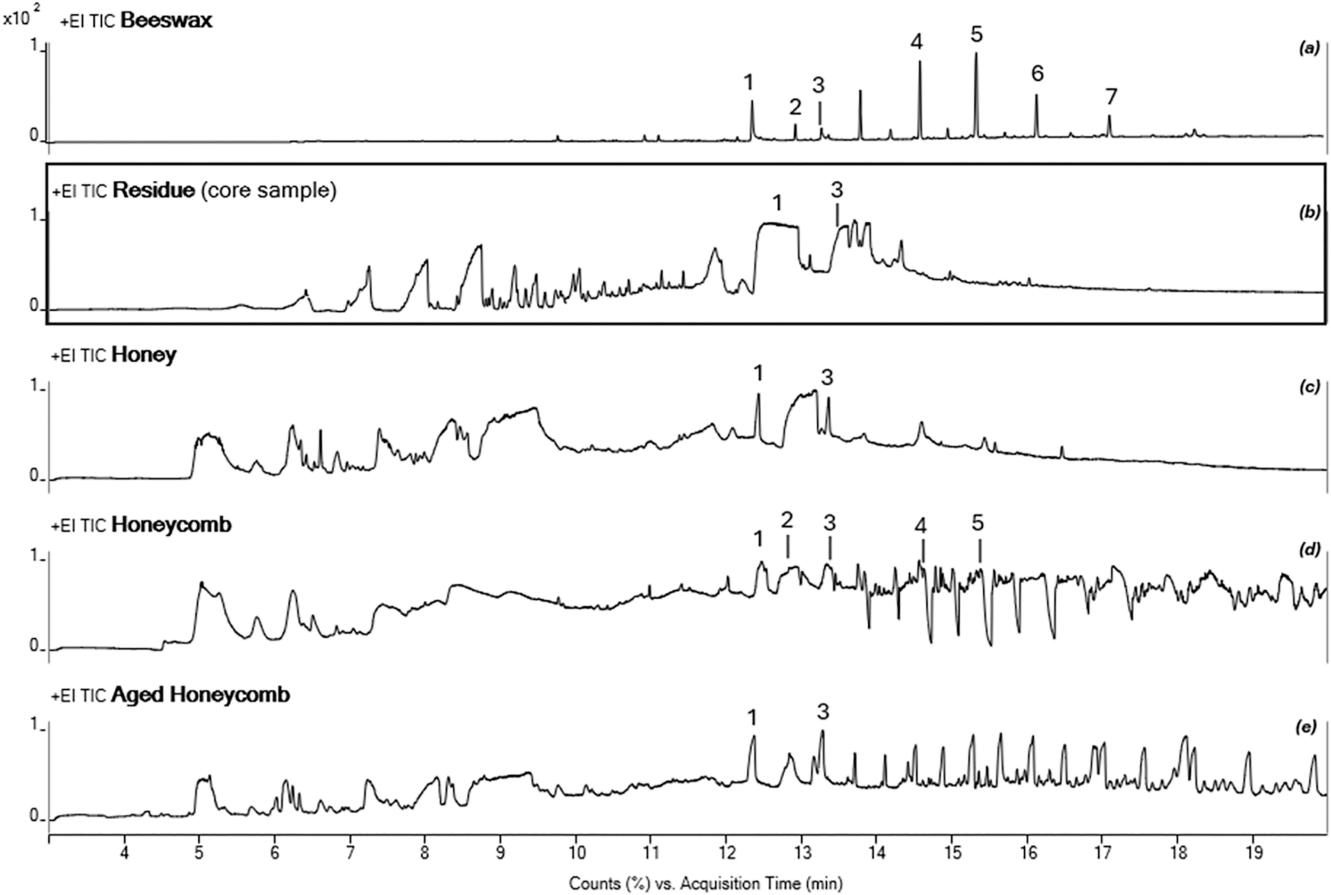
EI chromatograms from the TSP-GC/MS analysis of beeswax (a), the residue core sample (b), honey (c), and honeycomb from Greece, fresh (d) and aged (e). Compounds identified: [1] Hexadecanoic acid, [2] Heneicosane, [3] Octadecanoic acid, [4] Pentacosane, [5] Heptacosane, [6] Nonacosane, and [7] Hentriacontane. Compound identification spectral information is included in Supporting Information S2, Tables S1–S4.

As reports from previous studies of the residue did not state which part(s) of it had been sampled, we also analyzed samples from the surface of the residue, split according to color (i.e., orange, black, and green). Although the TIC obtained for these samples (Supporting Information S2, Figure S3) have a similar profile to that obtained for the residue’s core sample, it was possible to identify some compounds based on fragmentation patterns using extracted ion chromatogram. Among the compounds identified in all residue samples (Supporting Information S2, Table S5) were aliphatic aldehydes (C6, C7, and C9), midchain carboxylic acids (C7:0, C8:0, C9:0, C10:0, and C10:1), and long-chain carboxylic acids (C14:0, C16:0, C17:0, and C18:0). Two saccharide degradation compounds—5-methylfurfural and levoglucosenone—were detected only in the residue’s black surface sample.

We explored potential improvements in chromatographic resolution of the residue’s core sample via the analysis of its dichloromethane and methanol extracts (Supporting Information S2, Figure S4 and Table S10). The methanolic extract yielded a TIC largely composed of the same compounds detected in the direct analysis of the solid sample of the residue, identifiable by an extracted ion chromatogram. This confirmed that our compound identifications were not limited by sample overloading or volatility constraints.

### AEC-MS

These analyses yielded the identification of seven compounds based on comparison with authentic standards (Figure 4 and Supporting Information S2, Table S11), including hexose sugars (Supporting Information S2, Figures S5−S6), their abundance in the residue extract higher than in beeswax’s but lower than in the honey extract (as might be expected). Taurine, a free sulfur amino acid, was detected almost exclusively in the residue’s extract.

**Figure 4:**
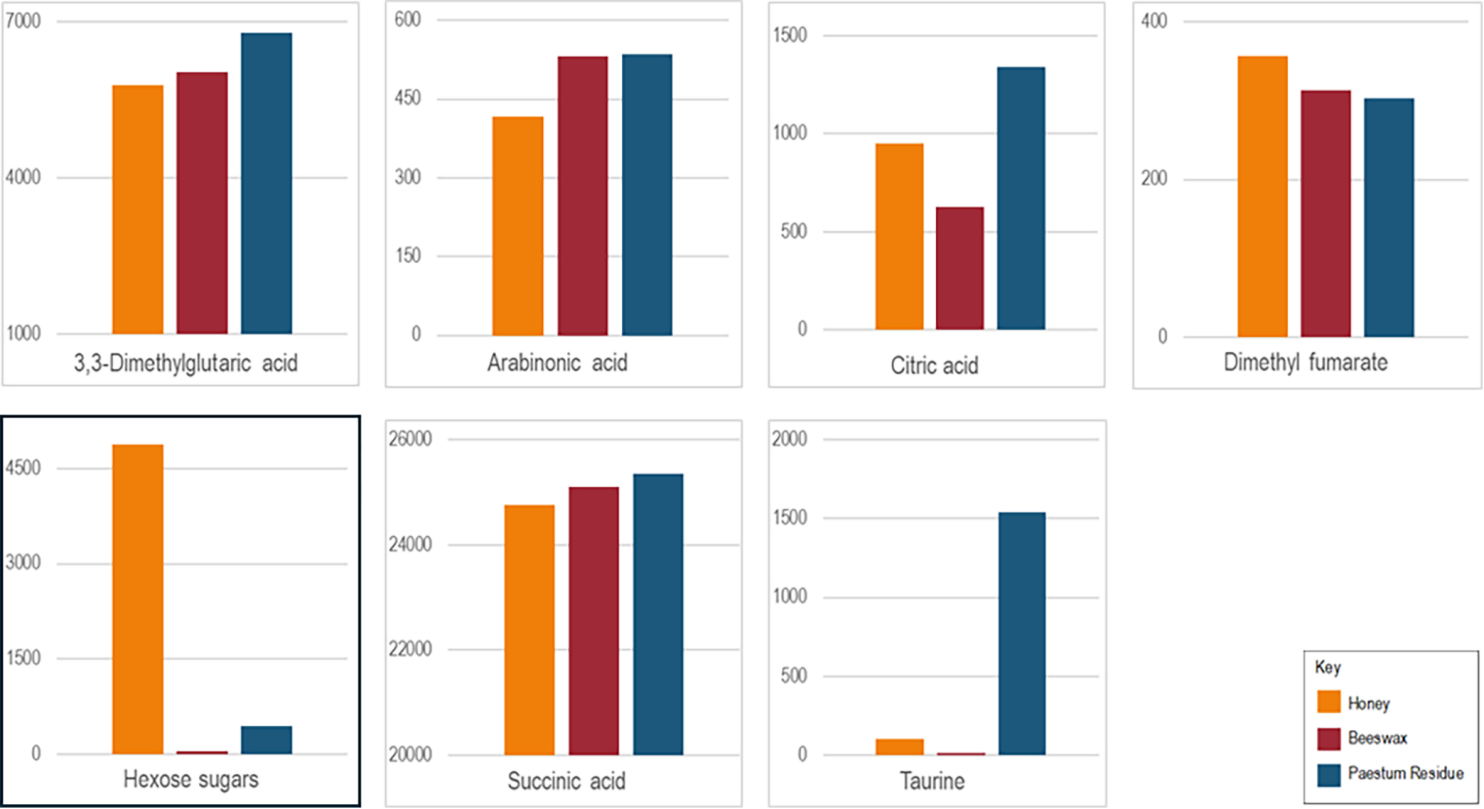
Raw abundances (expressed in ion counts) of compounds identified in aqueous extracts of honey, beeswax, and residue (core sample) by AEC-MS. Measurements (Supporting Information S2, Table S11 and Figures S5–S6) are based on one analysis per extract.

Hexose sugars were also detected in the aqueous extracts from honeycombs (Supporting Information, S2, Table S12), along with gluconolactone (a derivative of glucose) and galacturonic acid. Succinic, malic, and citric acids were present at much lower ion counts. All compounds exhibited higher relative abundances in extracts from fresh honeycombs compared to aged honeycombs (Supporting Information S2, Figure S7).

### Bottom-Up Proteomics

Only the residue’s core sample, beeswax and honeycombs were analyzed by this technique. Our untargeted search of the UniProt All proteins database^19^ using the peptides generated from digestion of the residue yielded matches for three primary royal jelly proteins derived from (the Western honeybee),^20^ several bacteria-derived proteins and a series of proteins commonly recognized contaminant proteins (e.g., keratins, caseins, lysyl endopeptidase, trypsin) listed in the cRAPCommon Repository of Adventitious Proteins database^21^ (Supporting Information S2, Table S13). This result led to a subsequent search of the UniProt Honey database. This search yielded 11 matches, the most significant are listed in Table 1 (the full list is included in Supporting Information S2, Table S14).

**Table 1:** Selected Protein Matches Obtained from the Paestum Residue Peptides against UniProt Honey Database[Table-fn t1fn9999]

protein	uniprot accession code^a^ taxa	–10 lg *P* ^b^	coverage (%)	#peptides (unique)	highest scoring unique peptides [peptide score (−10 lg *P* ^c^)]
major royal jelly protein 1 (MRJP-1)	**O18330**	156.20	26	10 (10)	R.TSDYQQNDIHYEGVQNILDTQSSAK.V [**64.42**]

major royal jelly protein 2 (MRJP-2)	**O77061**	147.70	13	5 (4)	K.IVNDDFNFDDVNFR.I [**71.40**]

major royal jelly protein 3 (MRJP-3)	**Q17060**	130.74	18	10 (7)	K.IINNDFNFNDVNFR.I [**65.53**]

actin 5C	**A0A2A3EM69**	127.07	17	5 (4)	K.SYELPDGQVITIGNER.F [**66.93**]

heat shock	**A0A2A3EG18**	74.11	12	5 (5)	K.VEIIANDQGNR.T [**48.18**]

trypsin-2	**A0A2A3ECX5**	47.43	11	2 (2)	K.DSC(+57.02)QGDSGGPMVAG.G [**36.88**]

actin 2	**A0A2H3EAA9**	69.38	13	3 (2)	K.DYELPDGQVITIGNER.F [**42.69**]

uncharacterized protein	**A0A1 V9XCK9**	41.78	34	4 (4)	K.GGQPARIQ(+.98)GGGQ(+.98)GSSGGGGGGGGGASGSGKKK.N [**22.10**]


a All high scored peptides present a good MS/MS spectrum match of b and y ions with an error map of 0 Da.

b Each code denotes a different biological source.

c The −10 lg *P* score indicates the statistical significance of the protein identification

d For each spectrum in the MS/MS data set, −10 lg *P* score gives the most likely correct peptide from the database search. Normally, a score >20 represents a relative high degree of confidence in its identification.

Similar searches against the UniProt All Proteins and UniProt Honey databases were carried out for the peptides obtained from fresh and aged honeycombs (Supporting Information S2, Tables S15–S22). The most significant protein matches common to the residue and the honeycombs are shown in Figure 5. No peptides were recovered from modern beeswax, which demonstrated that the peptides we recovered from the honeycombs derived from their honey fraction. Variability in major royal jelly proteins (MRJPs) detection was observed between the Greek and Italian honeycombs. This result was not unexpected, as the two honeycombs were visually distinct. Climate, temperature,^22^ and floral sources^23^ all affect protein expression in bees and their products. Moreover, postharvest handling and storage conditions can also affect the chemical composition of bee products.

**Figure 5:**
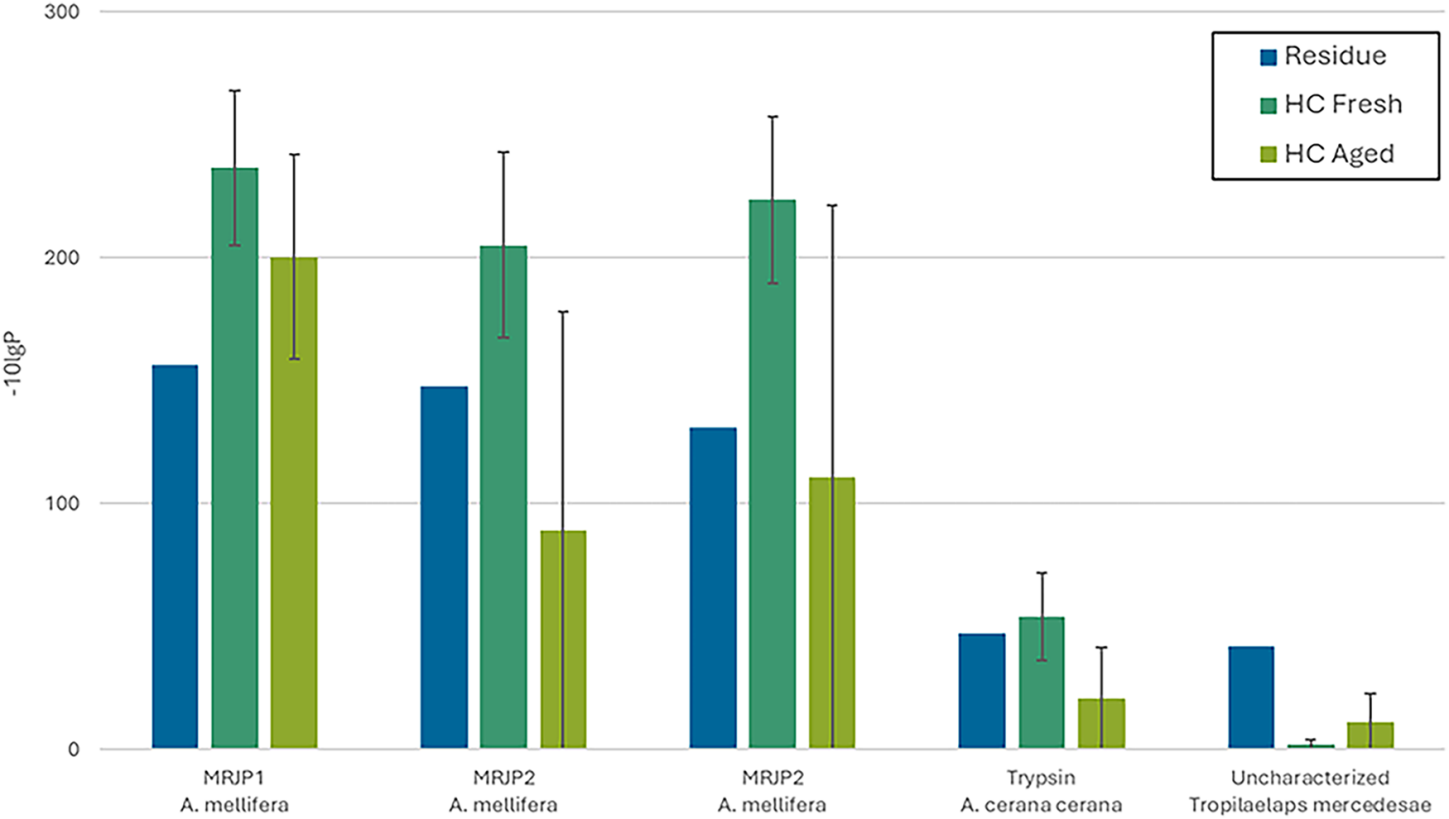
Common protein matches were obtained for the Paestum residue and fresh and aged honeycombs (HC). The error bars represent the standard deviation of values obtained for HC from Greece and HC from Italy obtained from one analysis per sample.

### XPS

We analyzed a single sample of the surface of the residue where green, black, and orange color (in this order) were represented. The elemental composition obtained for the green area was: 74.98% carbon, 20.78% oxygen, and 4.24% copper. For the black area, the composition was: 77.96% carbon, 20.12% oxygen, and 1.92% copper. And, for the orange area, the elemental composition was 86.50% carbon and 13.50% oxygen. In XPS, Cu^2+^ ions can be differentiated from Cu^1+^ ions by the presence of shakeup (or satellite) peaks in the Cu 2p spectrum, independent of the ligand.^24–26^ The Cu 2p spectra for the areas containing copper are shown in Figure 6, indicating the presence of Cu^1+^ ions in the black area and Cu^2+^ ions in the green area of the sample.

**Figure 6:**
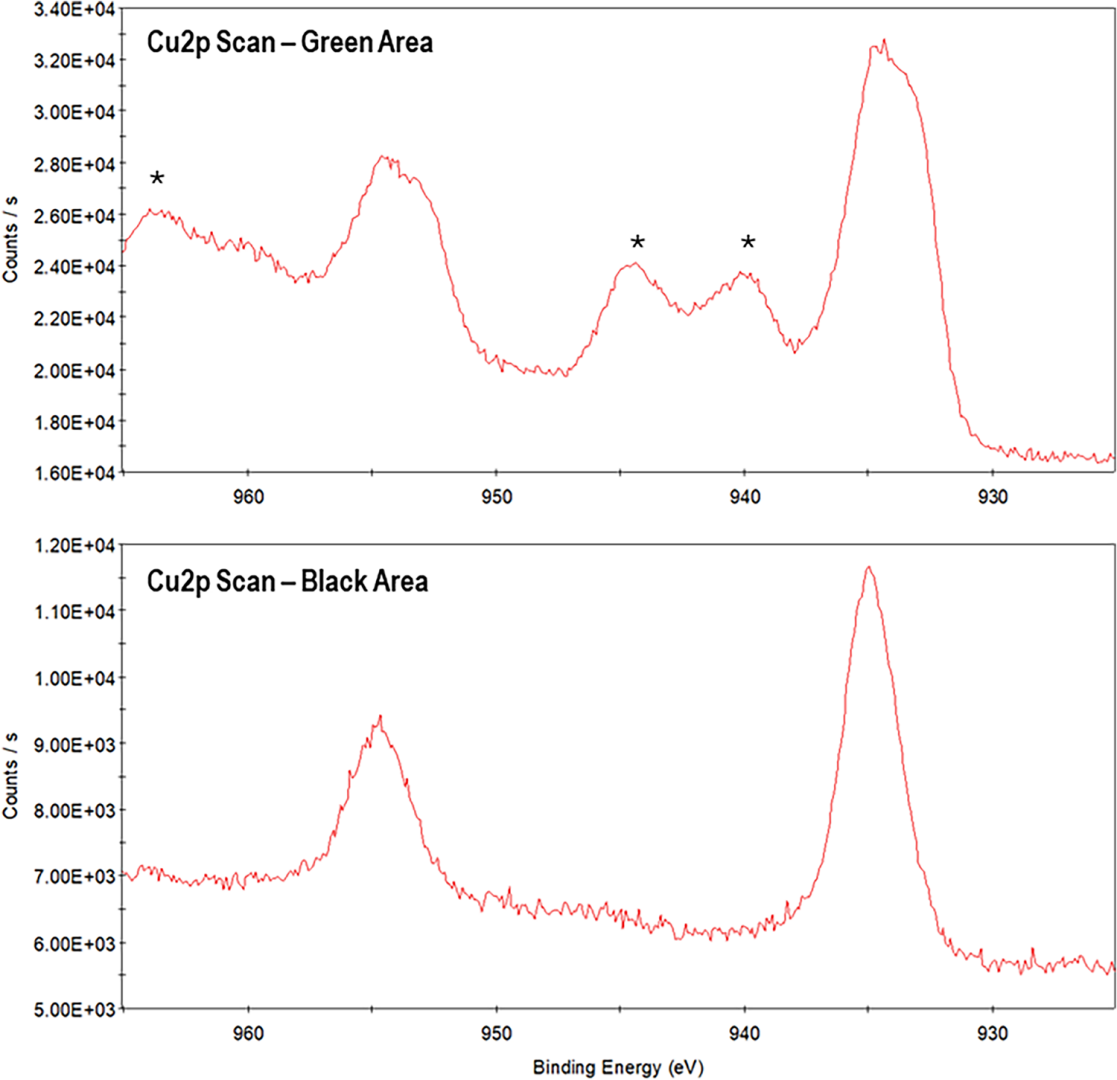
Detailed Cu 2p scans of green and black areas of surface sample of the residue; (*) denotes satellite peaks.

## Discussion

Fresh honey is composed of 79% hexose sugars (of which fructose is the most abundant at 39%), 18% water, 1.1% proteins, 0.17–1.17% acids (formic, citric and gluconic being the most abundant) and traces of vitamins, enzymes, flavonoids, and phenolic compounds.^27^ Over time, honey constituents undergo degradation, including through Maillard secondary reactions,^28^ changing in appearance and chemical composition. Maillard reactions occur between reducing sugars and amino acids in food when subjected to heat and/or storage. Their products are responsible for the change of color and taste of food, such as the development of a brown crust in baked bread. During long-term storage (particularly at temperatures above 20 °C), honey acquires a darker hue, sugars degrade into furans, and its acid content increases.^28^

The first analysis of the Paestum residue suggested that it was a wax. In antiquity, beeswax was almost invariably the one used.^29^ Beeswax has a very different composition to honey. Fresh beeswax is composed of 64% esters (35% are C40–C52 monoesters, predominantly alkyl palmitates), 14% odd medium-chain *n*-alkanes (C27H56 being the most abundant), 12% free acids, 2% acid polyesters, 1% C16–C20 acid monoesters, 1% free alcohols, and 6% of unidentified material.^30^ Although beeswax is considered highly stable because most of its alkanes and esters are saturated, its degradation is marked by an increased acid content, an increase in free alcohols from the hydrolysis of wax esters and the elimination of shorter chain alkanes.^7,31,32^

Our chemical profiling of the residue’s core sample by FTIR revealed similarities with modern beeswax in the functional group region of the spectrum except the position of the carbonyl group peak: in the residue, this peak appeared at 1703 cm^–1^ and in modern beeswax at 1736 cm^–1^. FTIR spectra of accelerated aged beeswax using heat^31^ and historical beeswax artifacts^33^ reported in the literature show a medium peak at around 1700 cm^–1^. We tried to simulate honey and beeswax’s increased acidity due to aging by accelerated aging of honeycombs using heat. Although at the end of the experiment, the honeycombs had acquired a darker hue, the chemical changes responsible for this change in color were not picked up by FTIR (Figures S1–S2). However, the similarities between the FTIR spectra of the residue and beeswax in the fingerprint region (1350–900 cm^–1^) are noteworthy. The spectra of the residue and beeswax overlap in this region, except for a strong peak at 1171 cm^–1^ observed in beeswax which corresponds to C–O esters and C­(CH_3_)_2_ skeletal alkane stretching vibrations.^17,34^ If the residue was beeswax, the reduction of this peak would correspond to an increase in acid content, as wax esters degraded into fatty acids overtime. However, our analyses of the residue by TSP-GC/MS suggested a composition much more complex than that of degraded beeswax.

Further historical analysis of the residue indicated that it could have been a resin or an animal/vegetable oil. Indeed, complex organic mixtures that yield an FTIR spectra featuring C–H aliphatic vibrations and a strong C=O peak in 1690–1705 cm^–1^ range would likely be assigned to the “tree resins and oils” category because they are rich in fatty acids.^35^ Fatty acids were among the compounds identified in our analysis of the residue samples by TSP-GC/MS but the relative intensity of their chromatographic peaks was much lower than the peaks corresponding to midchain acids. TSP-GC/MS analysis of the black surface of the archeological residue revealed the presence of 5-methylfurfural and levoglucosenone, key degradation products of saccharides. These compounds were notably absent from other areas of the residue, suggesting a localized preservation mechanism. Comparative analysis of fresh and aged honeycombs from Italy and Greece provided insights into the formation and stability of these saccharides markers. 5-Methylfurfural was detected in both types of fresh honeycombs but persisted only in the aged Greek honeycomb sample whereas levoglucosenone was identified exclusively in the aged Greek honeycomb (Supporting Information S2, Figure S8). This selective occurrence indicates a complex preservation pathway likely governed by environmental and chemical factors over time.

XPS analysis of the surface sample displaying a colored stratigraphy revealed the presence of Cu^2+^ ions in the outer green layer and Cu^1+^ ions in the black inner layer, originating from the copper vessel in which the residue was originally held.^12^ Copper alloy objects typically develop a thin layer of copper­(I) oxide upon air exposure, which provides them some protection against corrosion.^36,37^ However, over time, the degradation of the cork disc that originally sealed the bronze jar combined with the acidity of its content likely contributed to the formation of Cu^2+^ ions. But, in the presence of reducing monosaccharides, these ions would be reduced to Cu^1+^,^38^ creating a localized redox environment.

The biocidal properties of Cu^2+^ ions^39^ may have protected saccharides degradation products such as 5-methyl furfural and levoglucosenone from microbial breakdown, potentially explaining why these compounds were preserved in the black-colored area of the surface of the residue but absent from other areas. However, since these compounds were detected on the surface of the residue, the part most exposed to contamination, further investigation for the presence of saccharides had to focus on the residue core sample, where external contamination was less likely to have played a role in its composition.

To target hexose sugars in the residue, its water extract was analyzed by AEC-MS using a highly sensitive method for detection of metabolites in biological systems. It was necessary to analyze water extracts of honey and beeswax for comparison purposes, as the library of compounds used for compound identification was not exclusive to any specific type of biological sample. As expected, the highest abundance of ions corresponding to hexose sugars was detected in the water extract of modern honey. But, for the first time, hexose sugars were detected in the residue’s extract: their abundance was 10-fold higher than in the water extract obtained from modern beeswax. While honey was the only sweetener in the Mediterranean in antiquity,^40^ the presence of intact hexose sugars does not provide conclusive evidence for the presence of honey in the residue as such compounds are also present in fruits, various plant-derived substances (e.g., gums), or even degraded carbohydrate-rich foods.

An interesting finding was the strong signal for taurine observed in the residue’s water extract. Although taurine is the second most abundant amino acid in honeybee physiology,^41^ its occurrence in bee products such as royal jelly^42^ and honey^43^ is typically limited to trace levels. The primary sources of taurine are seafood, meat and milk.^44,45^ The presence of taurine in the Paestum residue may be from several possible sources. One possibility is the inclusion of milk in the original contents of the vessel. This would be reasonable given its ritual context.^46^ However, no clear evidence of lipid associated with dairy products was identified: the residue showed primarily palmitic acid (C16:0) over stearic acid (C18:0) whereas degraded animal fats normally show high abundances of both acids.^47^ This does not rule out the possibility that milk was originally present, but the evidence is equivocal.

Another explanation for the high abundance of taurine could be the result of the microbial activity. Taurine can be produced through the anaerobic degradation of sulfur-containing amino acids (e.g., cysteine and methionine) by microbes such as .^48^ The bronze vessels, sealed and buried in low-oxygen conditions for over two millennia, could have provided a favorable environment for such microbial activity. In this context, the high relative abundance of taurine in the residue may reflect microbial production rather than direct input from the original contents. Citric and succinic acids were detected in all extracts. Although common acids found in honey,^28^ they are also well-documented microbial metabolites.^49,50^

In an attempt to contextualize these findings, additional AEC-MS analyses were conducted on aqueous extracts of honeycombs (fresh and aged), resulting in the identification of six compounds including hexose sugars. The compound with the highest ion abundance in these samples was gluconolactone, a product of glucose oxidation (the other being hydrogen peroxide during the ripening of honey).^51^ Gluconolactone subsequently hydrolyzes to gluconic acid, coexisting in equilibrium, with specific conditions (e.g., temperature, pH) favoring the production of one substance over the other.^52^ Unfortunately, gluconic acid—the predominant organic acid in honey^53^—is not among the authentic standards in the library used for compound detection in this study.

Galacturonic acid was also detected in the honeycomb extracts, with an ion abundance slightly lower than that of the hexose sugars. Organic acids in honey vary depending on the floral source of the nectar. Studies linking organic acid profiles with nectar’s botanical sources have demonstrated that galacturonic acid is a biomarker for multifloral honeys^54^ and fir (coniferous) honeys.^55^ Thus, its absence from the acid profile of the honey extract (a monofloral honey from acacia tree) and its identification in the honeycomb extracts is not surprising, as coniferous trees are abundant in Greece and Italy, where the honeycombs analyzed in this study originate.

The effects of accelerated aging were also reflected in the AEC-MS results for the honeycombs. All compounds identified in their extracts exhibited higher relative abundance in fresh compared to aged honeycombs (Supporting Information S2, Figure S7). This may be attributed to increased enzymatic activity and chemical degradation processes promoted by the high temperatures used to simulate aging. However, the limited number of samples analyzed in this study restricts the statistical significance of these trends. Future work incorporating larger sample sets will be crucial for validating and expanding upon these preliminary observations.

Definitive evidence of the presence of bee products in the residue was obtained by bottom-up proteomics via the identification of major royal jelly proteins (MRJP) in the core of the residue. MRJPs are a series of nine homologous proteins detected in insects of the order *Hymenoptera*, which includes thousands of species of sawflies, ants, wasps, and bees.^56^ Representing the main proteins found in honey,^57^ they are secreted by the nurse bees’ cephalic glands and mixed with honey and pollen to feed the larvae in the hive.^58^

Our untargeted search for the origin of the peptides recovered from the residue using the UniProt All Proteins database yielded matches for MRJP-1, MRJP-2, and MRJP-3 from . As MRJP-1 from is the most significant MRJP found in honey,^59^ we carried out a subsequent targeted search against UniProt Honey database. This search yielded three additional proteins (Actin-5C, Heat shock, and Trypsin-2) from , the Eastern honeybee.^60,61^

Importantly, our findings go beyond taxonomic attribution. A recent study^62^ reported the identification of a single peptide from Arginine kinase in a residue extracted from an ancient Egyptian ceramic vessel dated to Ptolemaic-Roman period (forth century BCE–third century CE). In contrast, our analysis recovered multiple peptides specific to the MRJPs. These proteins are uniquely produced in the hypopharyngeal glands of worker bees and are the principal proteins in honey. As such, our findings offer stronger functional and taxonomic specificity.^63^ The detection of MRJPs in a heavily degraded, lipid-rich archeological matrix underscores the analytical depth and capability of our analytical approach. Other less statistically significant protein matches obtained for the residue’s peptides were for tree fungus^64^ and honeybee parasite .^65,66^

 and have evolved from a common ancestor,^67,68^ and this is reflected in the similarity of their proteomes. The differences in the amino acid sequence between MRJP-1 from and its equivalent in represent only 10% of the amino acid sequence, theoretically making the distinction between the species possible but difficult. The peptides recovered from the Paestum residue that were matched by PEAKS software to MRJP-1 from are listed in Supporting Information S2, Table S23. The second most intense peptide is R.IM (+15.99) NANVNELILNTR.C identified with only one post translation modification (i.e., oxidation). The peptide variations justify the taxonomic assignment of the origin of this protein to made by the software. However, because the three protein matches assigned to have identical amino acid sequences to similar proteins of ’s origin compounded by the fact that the entomological evidence for ancient beekeeping in Italy is also for ,^69^ we consider that this species is the correct origin of all honeybee protein matches predicted for the Paestum residue.

The proteins assigned to also merit some discussion. *Tropilaelaps* are species of parasitic mites^70^ that infect honeybees. Similar to *Varroa* mites,^71^ they are presumed to have originated in Asia and started to affect only in recent history. Thus, although it would be tempting to consider that any honey or honeycomb in the Paestum residue may have originated from Asia, it is more prudent to assume that the peptides predicted to be linked to represent common peptides from the *Acarid* family.

## Conclusions

A review of the prior analyses of the Paestum residue (summarized in Table S24 in the Supporting Information S2) reveals the long-standing uncertainty over the identity of the material. Over the past 70 years, assessments based on macroscopic examination, solubility tests, and early applications of gas chromatography couple to mass spectrometry consistently suggested the presence of waxes, fats, and/or resins—excluding honey as a component. The most detailed biomolecular characterization of the residue prior to this study was conducted in 1983, when the residue was analyzed by GC-MS. The resulting lipid profile, composed primarily of palmitic acid and other saturated and unsaturated fatty acids, was interpreted as evidence for an animal or vegetable fat. No sugars or glycerol were detected (though the precise method used is unclear). In contrast, our multianalytical study of the residue presents the first direct molecular evidence supporting the presence of honey, likely offered as honeycombs. This interpretation is supported by the identification of intact hexose sugars, saccharide decomposition products preserved within copper corrosion layers, peptides matching to major royal jelly proteins (MRJPs), and elevated acidity levels consistent with long-term degradation of honey and beeswax.

Direct comparison between previous and current analyses of the residue is complicated by methodological and experimental differences. The GC-MS analysis carried out in 1983 was likely conducted on a derivatized solvent extract optimized for lipids. In contrast, our use of thermal separation probe (TSP)-GC/MS enabled the detection of different classes of compounds while illustrating the chemical complexity of the residue including differences between the residue’s surface and the core, likely reflecting chemical interactions with the bronze vessel. The significantly increased sensitivity and mass accuracy of the mass spectrometer systems we used in our study (e.g., compared to those in previous studies) enabled us to measure a wider range of compound features, and the high resolution detection provided greater molecular specificity via chemical formulas prediction. This has provided us with a clearer picture of the material’s molecular composition at trace levels in addition to the main compounds present that make up the bulk of the residual material.

Traditionally, the detection of honey in archeological residues has focused on beeswax esters and saccharides. While the presence of other commodities, other bee products (e.g., propolis), plant oils, or milk, cannot be excluded from the original composition of the Paestum residue, our findings significantly expand the analytical toolkit available for investigating chemically complex archeological residues. By integrating TSP-GC/MS, AEC-MS, proteomics, and spectroscopy (FTIR and XPS), we have revealed the chemical complexity of the archeological residue and demonstrated the feasibility of detecting nonlipid biomarkers. Importantly, the recovery of multiple MRJP peptides, which are specific to honeybee secretions, provides greater functional specificity than other bee-related biomarkers recently reported in the literature, thereby strengthening the case for the intentional use of honeybee products in the original offering.

Finally, this study highlights the value of approaching ancient residue analysis through a hypothesis-driven framework. Given the complexity and diagenetic alteration of archeological residues, it is often more productive to test for the presence of a specific commodity than to attempt a fully open-ended reconstruction of the original material. The investigation of the Paestum residue began in 1957 with a focused question: was this residue originally honey/honeycomb? The same question guided our methodological approach and analytical choices for the current round of analyses of the residue, enabling a more robust, chemically, and archeologically grounded conclusion. We propose that this focused approach is especially valuable for future investigations of bee-derived products in legacy residues housed in museum collections, many of which have, until now, been considered analytically inaccessible.

## Supplementary Material





## Notes

Materials and Methods (PDF)
Data; FTIR spectra of fresh and aged honeycomb from Greece; EI Chromatograms for residue’s surface samples obtained with TSP-GC/MS; assignment of hexose sugars for unknown residue, honey and beeswax samples from mass spectra collected using AEC-MS; compounds identified in Modern Beeswax by TSP-GC/MS; protein matches obtained for Paestum Residue against Uniprot All Proteins Database; peptides recovered from the residue and matched to major royal jelly protein 1 (PDF)

## Abbreviations

FTIR: Fourier transform infrared spectroscopy
AEC-MS: anion exchange ion chromatography coupled to mass spectrometry
MRJP: major royal jelly protein
TIC: total ion chromatogram
TSP-GC/MS: gas chromatography coupled to quadrupole time-of-flight mass spectrometry analysis using a thermal separation probe
XPS: X-ray photoelectron spectroscopy
