# Supplementary material for: A Symbol of Immortality: Evidence of Honey in Bronze Jars Found in a Paestum Shrine Dating to 530–510 BCE
Source: J Am Chem Soc. 2025 Jul 30;147(33):29756–66. doi: 10.1021/jacs.5c04888 (PMC12371870; doi:10.1021/jacs.5c04888)
Supplement: Supplementary file 1 [file ja5c04888_si_001.pdf]

A symbol of immortality: evidence of honey in bronze jars found in a Paestum shrine dating to 530-510 BCE.

Luciana da Costa Carvalho*, Elisabete Pires, Kelly Domoney, Gabriel Zuchtriegel, and James S. O. McCullagh*

Supporting Information S1: Materials & Methods

1. Materials

The archaeological and modern samples analysed in this study, along with the respective methods used for their characterization, are listed in Table 1. All chemicals were analytical grade and sourced from Sigma-Aldrich, UK. To minimize the effects of post-excavation contamination and deterioration, analyses focused on a sample taken from the core of the residue. To control for laboratory contamination, we used clean stainless steel spatulas, disposable gloves, sterile containers and manipulated samples in the fume hood.

Samples analysed and analytical techniques used in this study

Samples	Spectroscopy		Chromatography coupled to Mass Spectrometry		
	FTIR	*XPS*	*TSP-GC/MS*	*AEC-MS*	*Proteomics*
Archaeological Residue					
Core	♦		♦	♦	♦
Surface (Black)		♦	♦		
Surface (Green)		♦	♦		
Surface (Orange)		♦	♦		
Modern Beeswax[1]	♦		♦	♦	♦
Modern Honey[2]	♦		♦	♦	
Modern Honeycombs (HC)					
Fresh HC from Italy[3]	♦		♦	♦	♦
Fresh HC from Greece[4]	♦		♦	♦	♦
Aged HC from Italy	♦		♦	♦	♦
Aged HC from Greece	♦		♦	♦	♦

Suppliers: [1]Abel & Cole (UK); [2] Conservation Resources (UK); [3] Apicoltura Cazzola via Amazon (UK) and [4] Sinanglou Farm, Pyrgos via Primefood Delicatessen (UK).

*Mass Spectrometry Research Facility, Department of Chemistry, University of Oxford, Oxford OX1 3TA, UK

2. Methods

2.1 Accelerated Aging of Honeycombs

A sample weighing around 4.5 g was taken with a stainless-steel spatula from each type of honeycomb (i.e. from Italy and from Greece) and placed at the bottom of individual 3.5 mL glass vials. The vials were closed with plastic caps with PTFE/silicone septum and heated at 70 ºC for 30 days in a block incubator. After this period, the vial contents were homogenized with a micro stainless-steel spatula prior to analysis.

2.2 Fourier Transform Infrared Spectroscopy (FTIR)

The archaeological residue's core, beeswax, honey and honeycombs (fresh and aged) were analysed by this technique. Samples were analysed in a Varian Excalibur FTS 3500 FTIR fitted with an Attenuated Total Internal Reflection (ATR) SpectraTech Golden Gate accessory containing a diamond crystal. Around 1mg of sample was smeared over the diamond crystal and just enough pressure applied to ensure good contact between them. Measurements were taken as a combination of 64 scans collected over a 4000–500 cm^{-1} range including background subtraction. The resolution was 4 cm^{-1} and the background spectrum was the blank diamond window. Data were processed using Digilab Resolutions Pro 4.0 software, and figures were created with Spectragryph v.1.2.12. Band assignments were based on FTIR tables and published articles.

2.3 Gas Chromatography coupled to Quadrupole Time-of-Flight Mass Spectrometry with a thermal separation probe (TSP-GC/MS)

The archaeological residue (core and surface samples), beeswax and honey were analysed by this technique. Around 1mg of sample was placed in a disposable glass microvial. The microvial was placed inside the thermal separation probe and analysed as reported (Carvalho, Henry et al. 2022) in an Agilent 7890B gas chromatograph equipped with a Restek Rxi-5 ms column (30 m × 320 µm × 0.25 µm) and connected to an Agilent 7250 GC/Q-TOF equipped with a low-energy-capable EI source (70 eV). The TSP was set at 300 ºC and the oven temperature was set at 40 ºC for one minute, increasing by 20 ºC/minute until it reached 320 ºC where it was held for five minutes. Helium was the carrier gas set at 1.43 mL/min flow rate and 8.70 psi pressure. The equilibration time was set at 0.5 min, and the sample injection was splitless. The mass range was 50 to 650 m/z. Agilent Mass Hunter Qualitative Analysis 10.0 was used for data analysis and compounds were identified by comparing their fragmentation spectra against the NIST (EI) Mass Spectral Library database.

Dichloromethane and methanol extracts were obtained from the archaeological residue core sample by adding 1 mL of solvent to a clean vial containing 1mg of residue, subjected to an hour sonication. After centrifugation, 10 µL of the extract was transferred to a clean glass

microvial using a micropipette and the vial placed in the fumehood for around 10-25 minutes to allow the solvent to evaporate prior to analysis by TSP-GC/MS under the conditions described above.

2.4 Anion-exchange Ion-Chromatography coupled to Mass Spectrometry (AEC-MS)

The archaeological residue's core, beeswax and honey were analysed by this technique. Around 1mg of sample was extracted with 1000μL of milli-Q water in a glass vial under sonication for 10 minutes. A 10μL aliquot of this extract was transferred to a disposable vial and analysed as reported (Walsby-Tickle, Gannon et al. 2020) using a Dionex ICS-5000 + high-pressure ion chromatography system equipped with a continuously regenerated trap column, Dionex ERS 500e suppressor and AS11-HC (2 × 250 mm, 4μm) column. This system was coupled directly to a Thermo Fisher Q-Exactive HF hybrid quadrupole-Orbitrap MS via a HESI II probe. The system incorporated an electrolytic anion generator (KOH) programmed to produce a 5 to 100 mM aqueous OH- gradient over 37 min. The column temperature was set at 30 ºC, and all samples were injected with a 5 μL partial loop injection. An inline electrolytic suppressor removed the OH^- ions and cations from the post-column eluent prior to its delivery to the electrospray ionisation source of the mass spectrometer.

The mass spectrometer was operated in negative ion mode set as follows: 60 sheath gas flow rate; 20 auxiliary gas flow rate; ; 3.6 kV spray voltage; 300 ºC capillary temperature; 70 S-lens RF level; 350ºC heater temperature. MS and MS/MS scan parameters were: microscans, 2; resolution, 7 × 104; AGC target, 1 × 106 ions; maximum IT, 250 ms; loop count, 10; MSX count, 1; isolation window, 2.0 m/z; collision energy, 35; minimum AGC target, 5 × 103 ions; apex trigger 1–15 s; charge exclusion 3–8, >8; dynamic exclusion, 20.0 s.

Progenesis software (Waters, UK) was used for data analysis. Compound searches were undertaken against an in-house database of 421 authentic standards using a 5 ppm mass accuracy threshold along with retention time and isotope pattern matching. Only compounds with an isotope accuracy of >90% were shortlisted for manual selection according to the highest fragmentation score and lowest retention time deviation.

2.5 Bottom-up Proteomics

The archaeological residue's core, beeswax, and honeycombs (fresh and aged) were analysed by this technique The method used is described in detail elsewhere (Pires, Carvalho et al. 2021). In summary, RIPA Buffer was used to extract proteins from around 5 mg of sample, and the protein extract was digested with LysC and trypsin. After desalting and concentration using C18 ZipTip the digested sample was re-suspended in milli-Q water with 2% acetonitrile and 0.1% formic acid, and 2 μL analysed by nanoLC-MS/MS using a Waters NanoAcquity-UPLC system interfaced with a Thermo LTQ Velos Orbitrap Elite mass spectrometer possessing an EASY-Spray ion source. Initial peptide trapping was carried out on a packed guard column (75 μm i.d. × 20 mm, Acclaim Pep-map100 C18, 3 μm, 120 Å) with 0.1% formic acid in deionized water at 140 bar. The peptides were separated on an EASY-spray

Acclaim Pep-Map® analytical column (75 µm i.d. × 15 mm, RSLC C18, 3 µm, 100 Å) using a 120 min linear gradient ranging from 3 to 97% of 0.1% formic acid in acetonitrile (300 nL/min flow rate and 40 ºC column temperature). The nanoESI source operated at 2100 V needle voltage with the ion transfer tube set to 275 ºC.

Peptides were ionised via electrospray directly into the mass spectrometer, operating in a data-dependent acquisition mode using a CID-based method (350–1500 m/z mass range, 120000 resolution, 1e6 AGC target, and 250 ms maximum injection time. The subsequent CID MS/MS spectra (AGC target 5e4, maximum injection time 100 ms) of the 10 most intense peaks were acquired in the Ion Trap, and fragmentation performed at 35% of normalised collision energy. The signal intensity threshold was kept at 500 counts.

Data analysis was performed using PEAKS 8.5v. Raw MS data was used for searches against UniProt All Proteins database (UniProt Release 2020-06) and identifications confirmed with UniProt taxonomy databases (UniProt Release 2020-06). For these searches LysCTryp was selected as the protease. Carbamidomethylation (Cysteine) was set as a fixed modification and Oxidation (Methionine), Deamination (Asparagine, Glutamine) and + 12 Da on N-terminal proline set as variable modifications. Precursor mass tolerance was set as 15 ppm, and fragment mass tolerances for CID were set to 0.8 Da. Only peptides with a -10lgP > 20 (P-value of 1%) were selected for PSM validation with a Target Decoy PSM Validator node based on q-values at a 5% false discovery rate (FDR) and after validation used in protein database searches.

PEAKS software uses an LDF (linear discriminative function) score to evaluate the accuracy of the peptide-spectrum match. Apart from matching the fragment ions and the peaks, the LDF score considers several other factors, such as the similarity between the de novo sequencing peptide and the database peptide. The LDF score is then converted to a p-value to make it easier for humans to understand. For a large dataset such as the UniProt All Proteins database, it is recommended to keep the FDR (false discovery rate) below 5%.

2.6 X-ray Photoelectron Spectroscopy (XPS)

Only samples from the archaeological residue's surface were analysed by this technique. A sample (estimated to weigh around 2 mg) of the surface of the archaeological residue containing orange, black and green-coloured areas was squashed onto a piece of graphite tape and spectra obtained with a Thermo Scientific K-Alpha X-ray photoelectron spectrometer calibrated for a carbon peak at 284.80 eV. A flood gun was used to stop the sample charging and the spot size was 400 µm. For the survey scan, a step size of 1 eV and dwell time of 10 ms was used and, for a detailed scan, a step size of 0.1 eV and dwell time of 50 ms. Two spots were measured for each coloured area, with peak fittings undertaken using Casa software with assignments and, where applicable, articles in the literature. Sample mounting and analyses were performed by XPS specialist Dr Phillip Holdway at Begbroke Science Park, Oxford.
