# Supplementary material for: A Symbol of Immortality: Evidence of Honey in Bronze Jars Found in a Paestum Shrine Dating to 530–510 BCE
Source: J Am Chem Soc. 2025 Jul 30;147(33):29756–66. doi: 10.1021/jacs.5c04888 (PMC12371870; doi:10.1021/jacs.5c04888)
Supplement: Supplementary file 2 [file ja5c04888_si_002.pdf]

A symbol of immortality: evidence of honey in bronze jars found in a Paestum shrine dating to 530-510 BCE.

Luciana da Costa Carvalho*, Elisabete Pires, Kelly Domoney, Gabriel Zuchtriegel, and James S. O. McCullagh*

Supporting Information S2 - DATA

Figure S 1 – FTIR spectra of fresh and aged honeycomb from Greece....2
Figure S 2 - FTIR spectra of fresh and aged honeycomb from Italy....2
Figure S 3 – EI Chromatograms for residue's surface samples obtained with TSP-GC/MS....3
Figure S 4 - EI Chromatograms for dichloromethane and methanol extracts from residue's core sample obtained with TSP-GC/MS. 4
Figure S 5 – Assignment of hexose sugars for unknown residue, honey and beeswax samples from mass spectra collected using AEC-MS:....5
Figure S 6 - The extracted ion chromatogram (top) for ions [M-H]- representing C6H12O6 (5ppm m/z range) from analysis of a mixture of 200 authentic metabolites that included hexose sugars (fructose, mannose and galactose) provided the same elution profile (at the same retention time) as for the residue, honey and beeswax samples....6
Figure S 7 - Raw abundances (expressed in ion counts) of compounds identified by AEC-MS in aqueous extracts of fresh and aged honeycombs, being the mean values of the measurements reported in Table S7. The error bars represent the standard deviation of measured obtained for honeycomb from Greece and honeycomb from Italy, obtained from one analysis per sample....7
Figure S 8 – Spectra for saccharides decomposition products identified by TSP-GC/MS in fresh and aged honeycombs (HC) from Greece and Italy....8

Table S 1 - Compounds identified in Modern Beeswax by TSP-GC/MS....9
Table S 2 - Compounds identified in Modern Honey by TSP-GC/MS....10
Table S 3 - Compounds identified in Fresh Honeycomb from Greece by TSP-GC/MS....11
Table S 4 - Compounds identified in Aged Honeycomb from Greece by TSP-GC/MS....12
Table S 5 – Compounds identified in the Paestum residue by TSP-GC/MS analysis and corresponding Match Factor / Reserve Match Factor*....12
Table S 6 - Compounds identified in the Paestum Residue Core Sample by TSP-GC/MS....13
Table S 7 Compounds identified in the Paestum Residue Surface Orange-colored Sample by TSP-GC/MS....14
Table S 8 - Compounds identified in the Paestum Residue Surface Black-colored Sample by TSP-GC/MS....15
Table S 9 - Compounds identified in the Paestum Residue Surface Green-colored Sample by TSP-GC/MS....16
Table S 10 - Compounds identified in the methanol extract of the Paestum Residue's Core Sample by TSP-GC/MS....17
Table S 11 Compounds identified in the H2O extract of honey, beeswax and Paestum residue (core sample) by AEC-MS....19
Table S 12 Compounds identified in the H2O extracts of fresh and aged honeycombs by AEC-MS....19
Table S 13 - Protein matches obtained for Paestum Residue against Uniprot All Proteins Database....20
Table S 14 - Protein matches obtained for Paestum Residue against Uniprot Honey Database....34
Table S 15 - Protein matches obtained for Honeycomb Greece Fresh against Uniprot All Proteins Database....37
Table S 16 – Protein matches obtained for Honeycomb Greece Fresh against Uniprot Honey Database....41
Table S 17 – Protein matches obtained for Honeycomb Greece Aged against Uniprot All Database....43
Table S 18- Protein matches obtained for Honeycomb Greece Aged against Uniprot Honey Database....50
Table S 19 - Protein matches obtained for Honeycomb Italy Fresh against Uniprot All Proteins Database....51
Table S 20 – Protein matches obtained for Honeycomb Italy Fresh against Uniprot Honey Database....54
Table S 21 – Protein matches obtained for Honeycomb Italy Aged against Uniprot All Proteins Database....55
Table S 22 - Protein matches obtained for Honeycomb Italy Aged against Uniprot Honey Database....59
Table S 23 - Peptides recovered from the residue and matched to major royal jelly protein 1 Apis mellifera, taxonomically informative residue and amino acid variants highlighted....60
Table S 24 - The analytical journey of the Paestum's residue....61

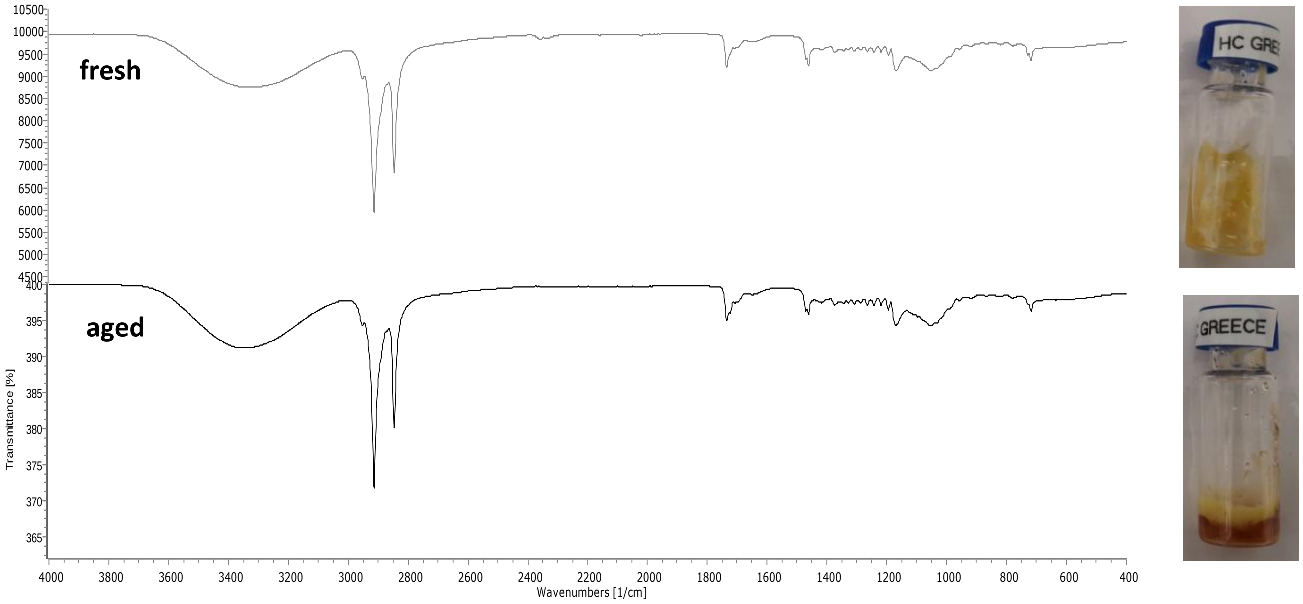

Figure S 1 – FTIR spectra of fresh and aged honeycomb from Greece

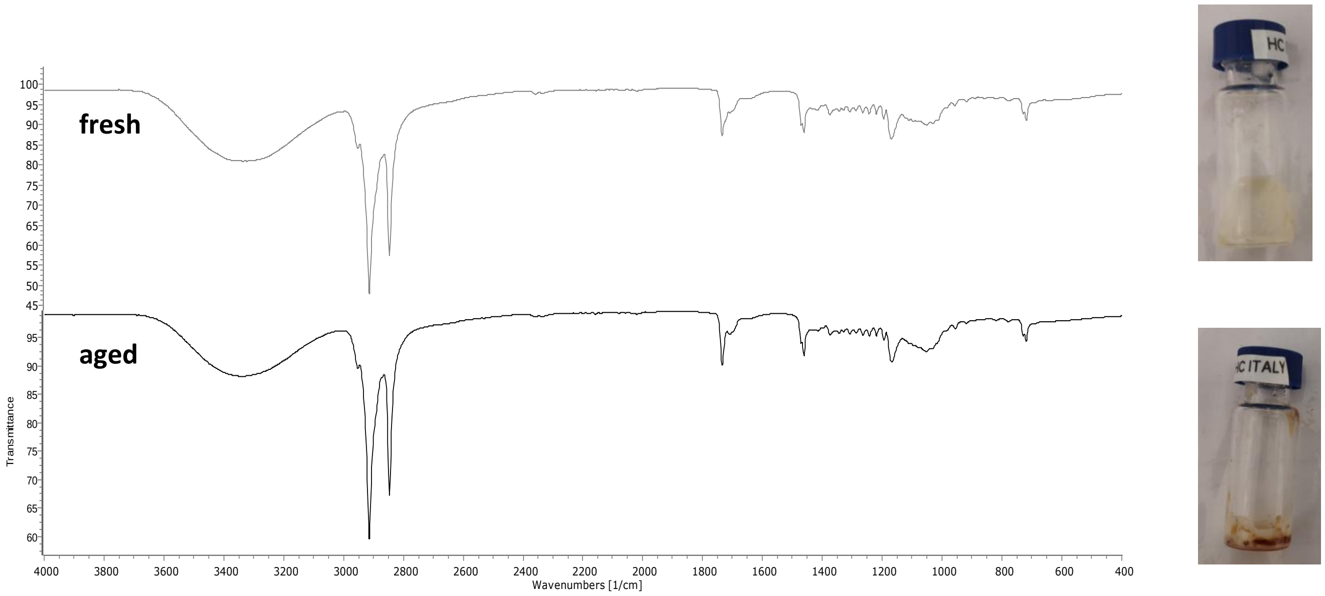

Figure S 2 - FTIR spectra of fresh and aged honeycomb from Italy

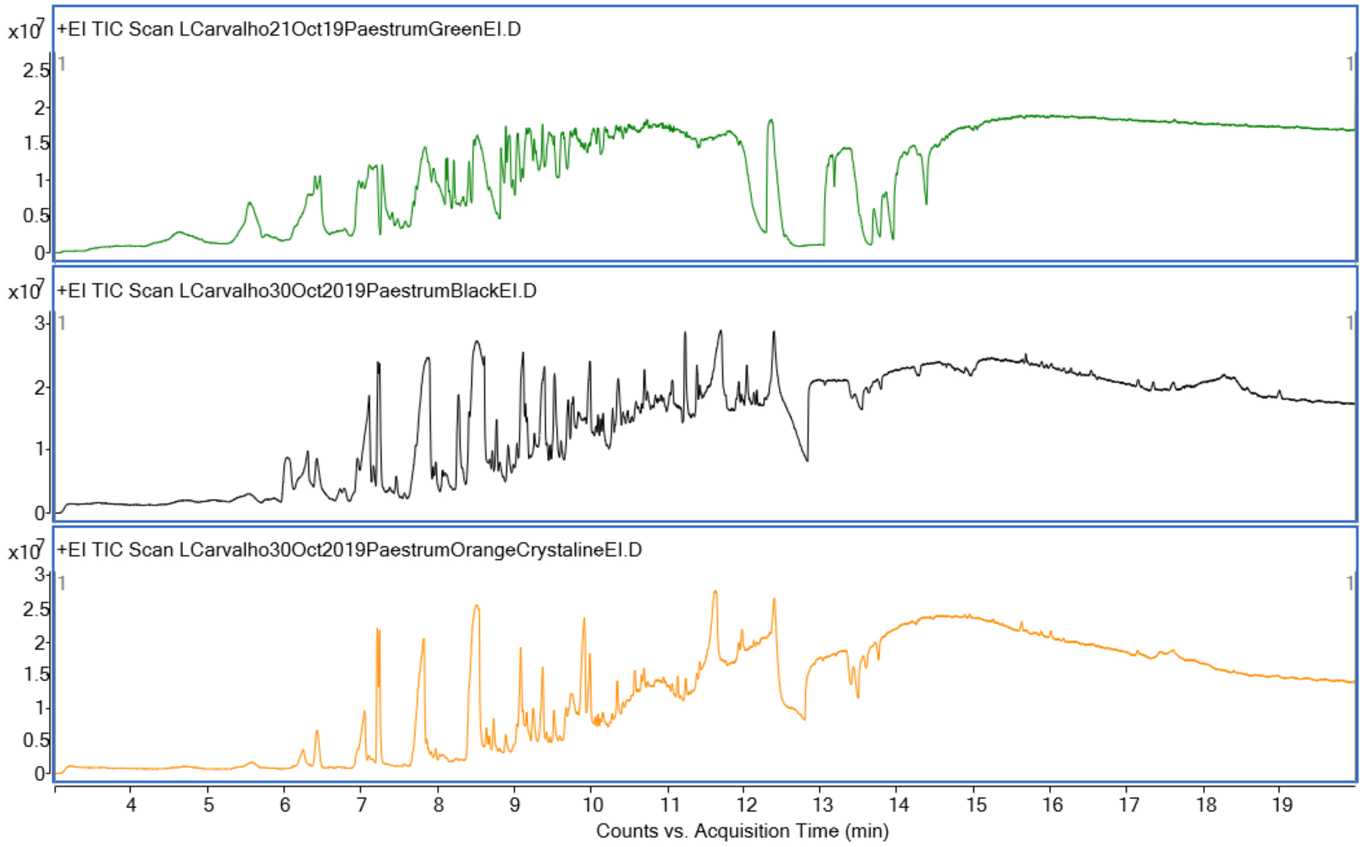

Figure S 3 – EI Chromatograms for residue's surface samples obtained with TSP-GC/MS

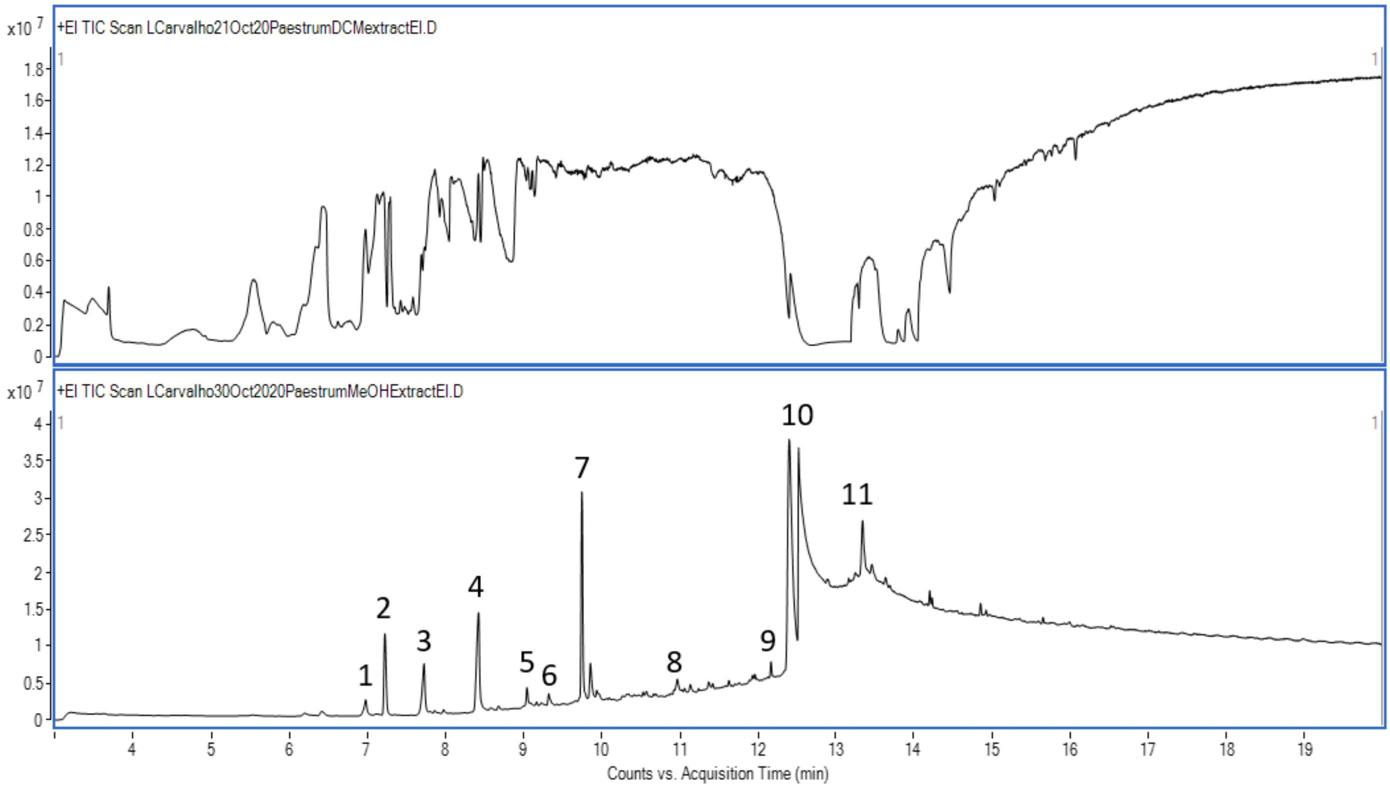

Compounds identified in the methanol extract: [1] heptanoic acid; [2] nonanal; [3] octanoic acid; [4] nonanoic acid; [5] decanoic acid; [6] decenoic acid; [7] dodecanol; [8] azelaic acid; [9] hexadecenoic acid methyl ester; [10] hexadecanoic acid (peak split into two) and [11] octadecanoic acid. Spectral information for compound identifications included in Table S5.

Figure S 4 - EI Chromatograms for dichloromethane and methanol extracts from residue's core sample obtained with TSP-GC/MS.

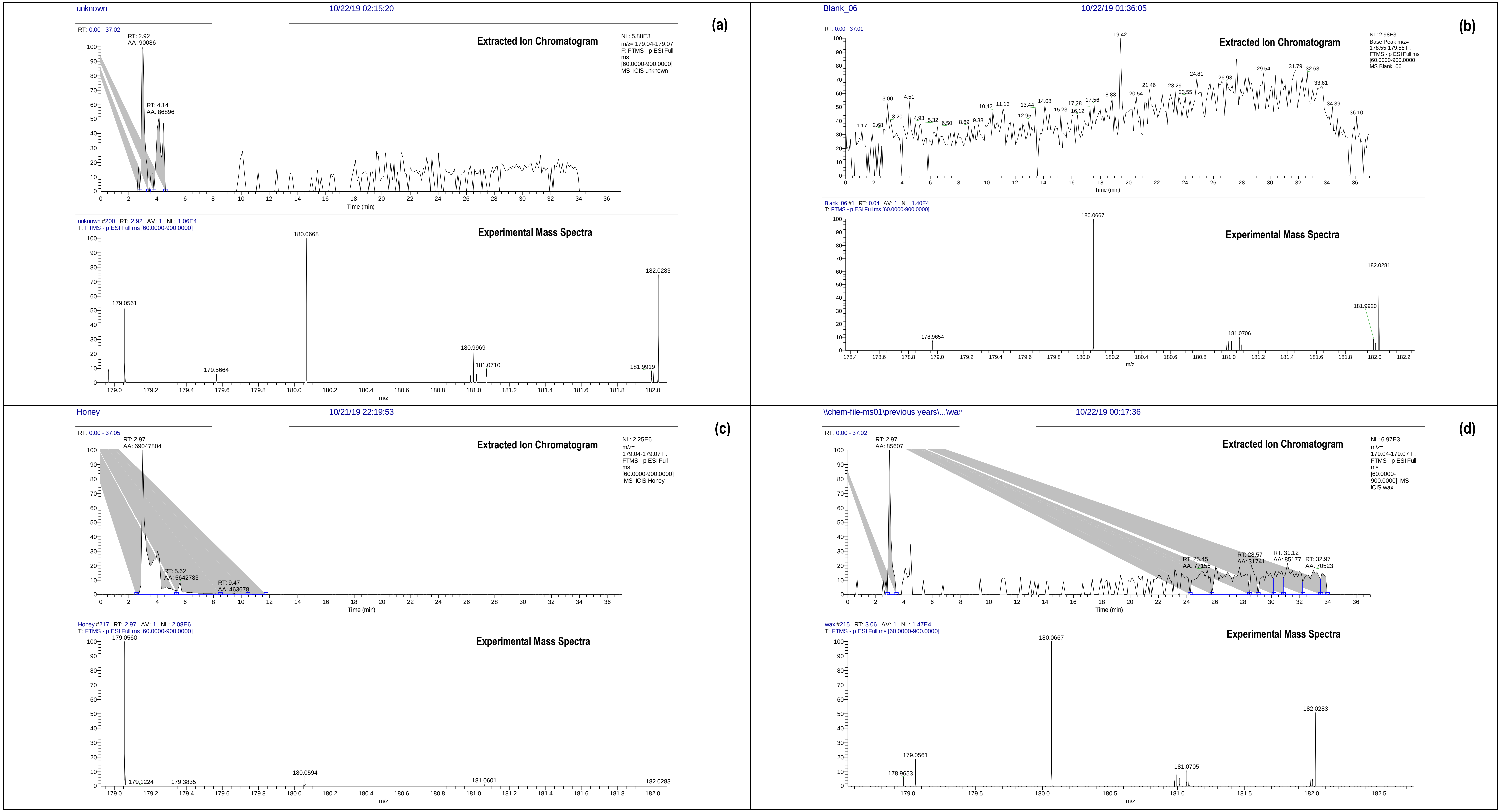

Figure S 5 – Assignment of hexose sugars for unknown residue, honey and beeswax samples from mass spectra collected using AEC-MS:

(a) 'unknown' sample extracted ion chromatogram (top) for ions $[M-H]^-$ representing C6H12O6 (5ppm *m/z* range) cluster around 2.9 mins. The experimental mass spectra shown below at *m/z* 179.0551. **(b)** Solvent blank sample run prior to 'unknown' samples shows no EIC signal at the 5ppm *m/z* range representing C6H12O6, controlling for any over between samples. The experimental mass spectra below shows the absence of ions at *m/z* 179.0551. **(c)** Authentic honey sample extracted ion chromatogram (top) for ions $[M-H]^-$ representing C6H12O6 (5ppm *m/z* range) shows the same chromatographic peak profile with the experimental mass spectra shown below at *m/z* 179.0551. **(d)** Beeswax sample extracted ion chromatogram (top) for ions $[M-H]^-$ representing C6H12O6 (5ppm *m/z* range) shows a weaker signal at the same Rt (2.97) chromatographic peak profile. The experimental mass spectra (below) shows presence of *m/z* 179.0551.

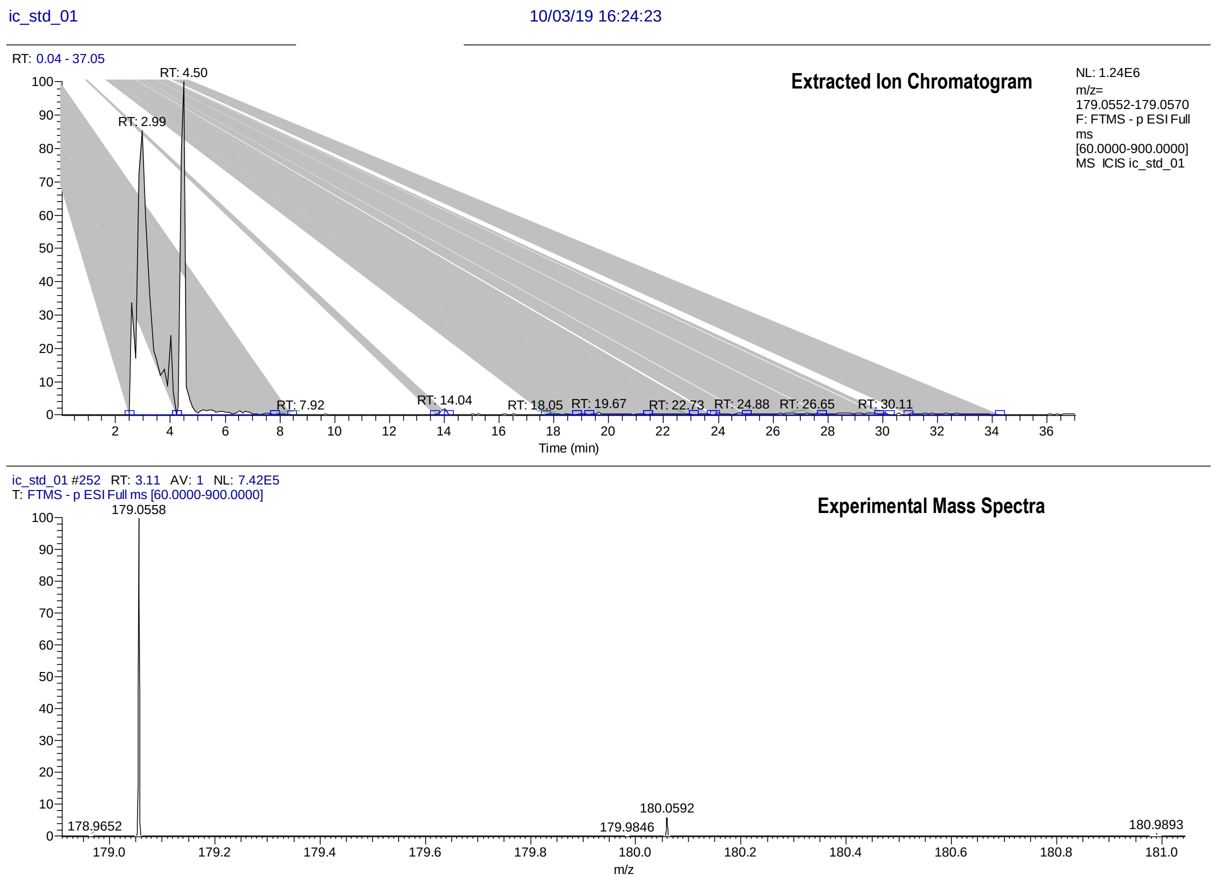

Figure S 6 - The extracted ion chromatogram (top) for ions [M-H]- representing $C_6H_{12}O_6$ (5ppm m/z range) from analysis of a mixture of 200 authentic metabolites that included hexose sugars (fructose, mannose and galactose) provided the same elution profile (at the same retention time) as for the residue, honey and beeswax samples.

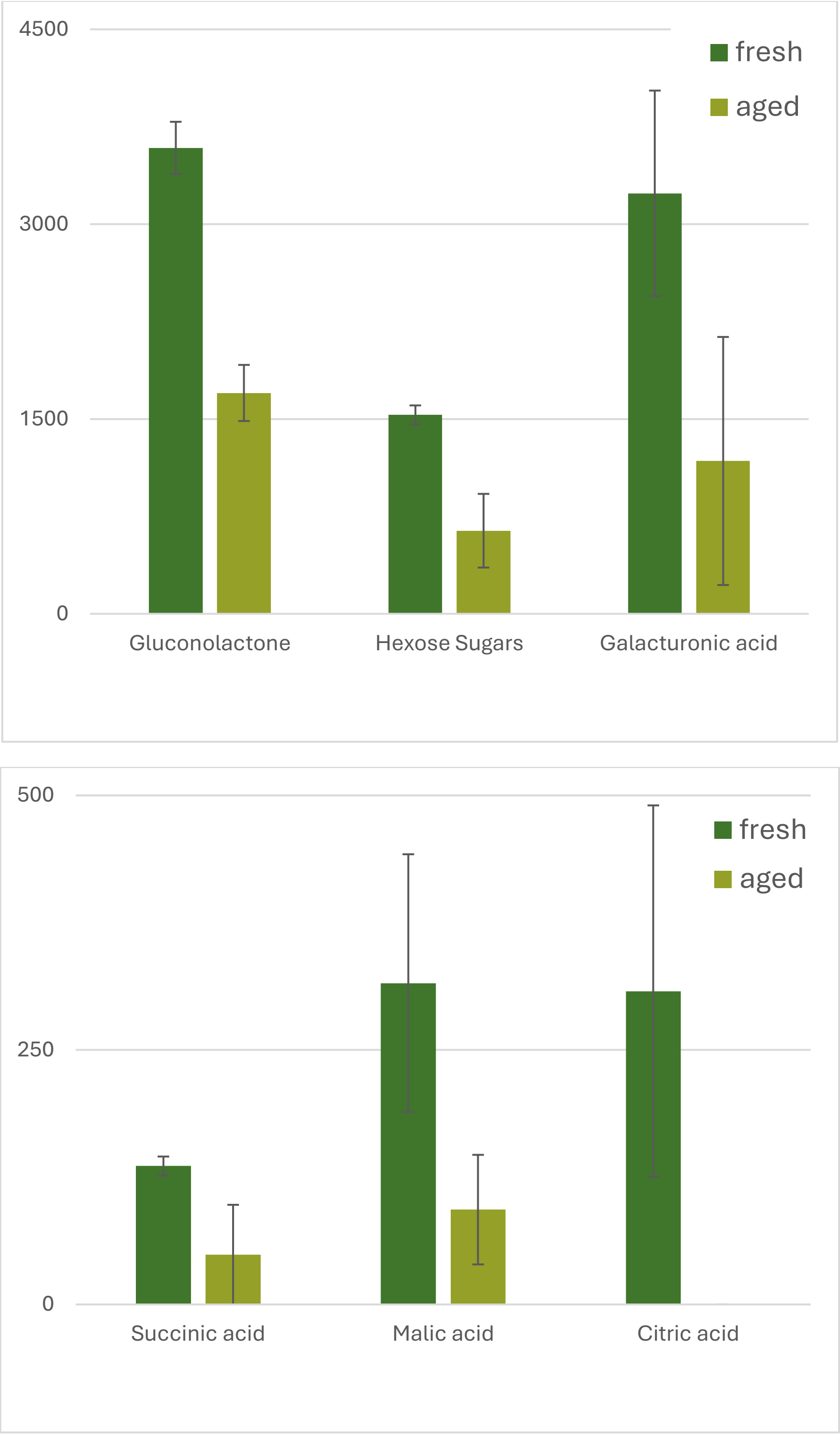

Figure S 7 - Raw abundances (expressed in ion counts) of compounds identified by AEC-MS in aqueous extracts of fresh and aged honeycombs, being the mean values of the measurements reported in Table S7. The error bars represent the standard deviation of measured obtained for honeycomb from Greece and honeycomb from Italy, obtained from one analysis per sample.

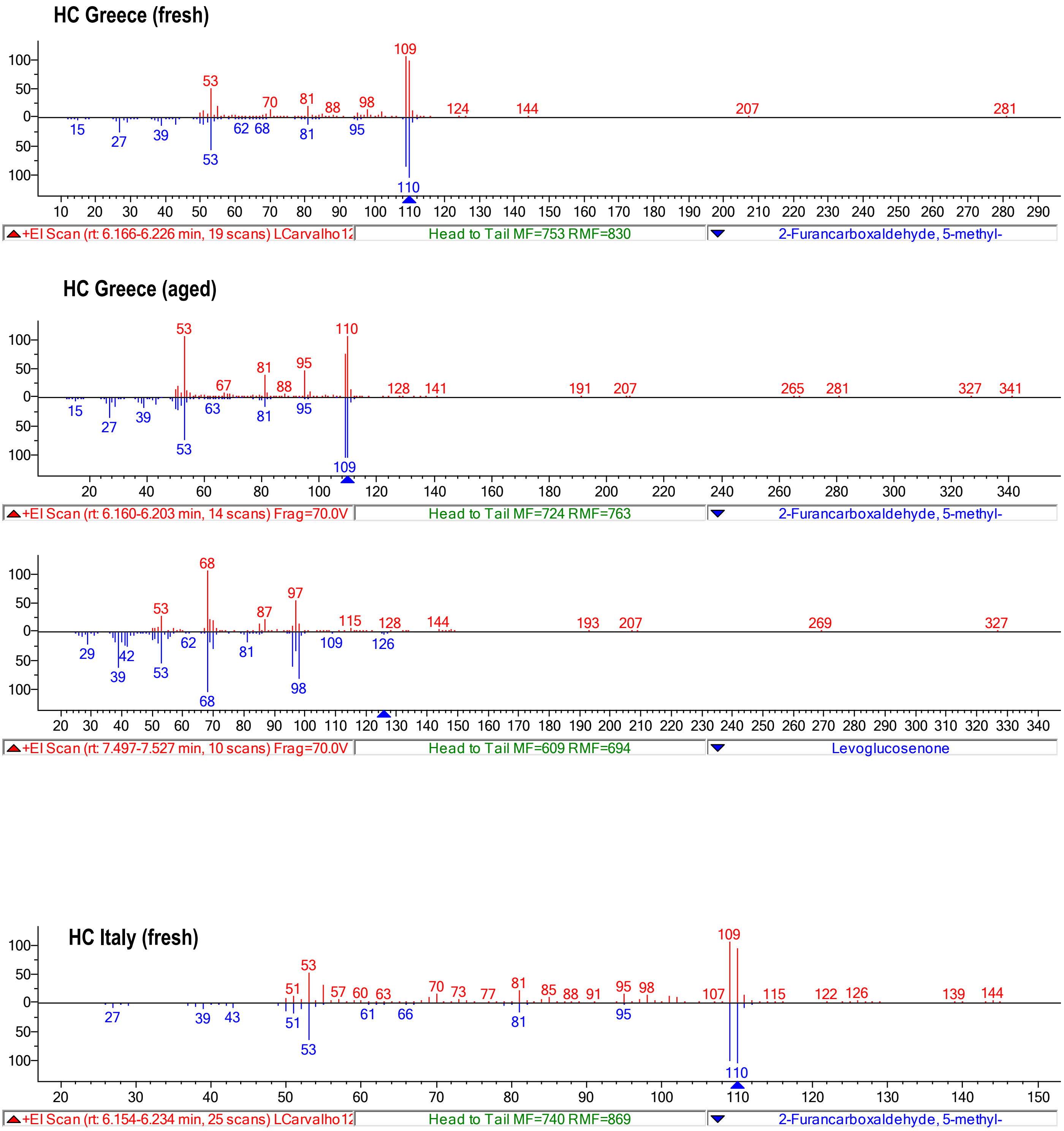

Figure S 8 – Spectra for saccharides decomposition products identified by TSP-GC/MS in fresh and aged honeycombs (HC) from Greece and Italy

Table S 1 - Compounds identified in Modern Beeswax by TSP-GC/MS

RT (min)	Compound Matching unknown in RED (top) and matched compound in BLUE (bottom)
12.4	Hexadecanoic acid – Theoretical *mz* 256.2402 – Experimental *mz* 256.2422 – Error: 7.80 ppm 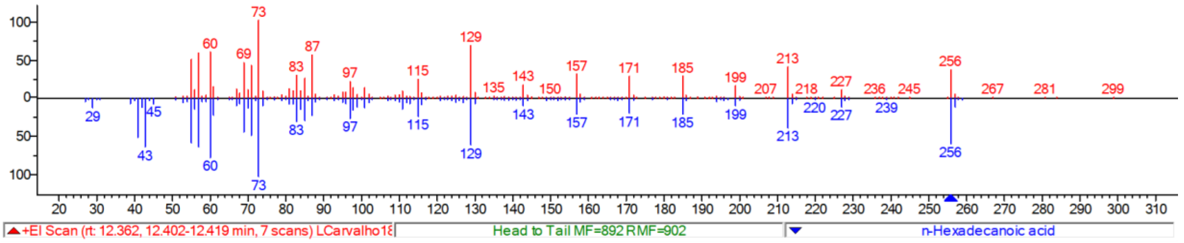
13.0	Heneicosane– Theoretical *mz* 296.3443 – Experimental *mz* 296.3454 – Error: 3.71 ppm 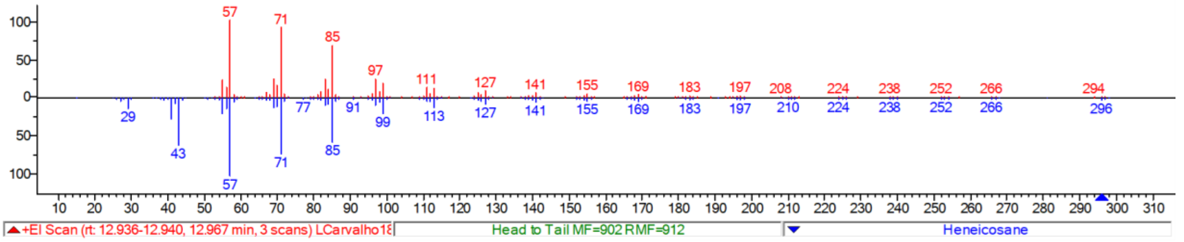
13.3	Octadecanoic acid – Theoretical *mz* 284.2715 – Experimental *mz* 284.2737 – Error: 7.74 ppm 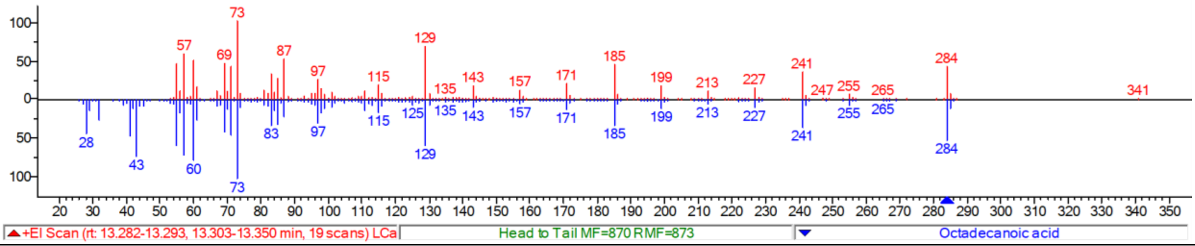
14.6	Pentacosane – Theoretical *mz* 352.4069 – Experimental *mz* 352.4070 – Error: 0.28 ppm 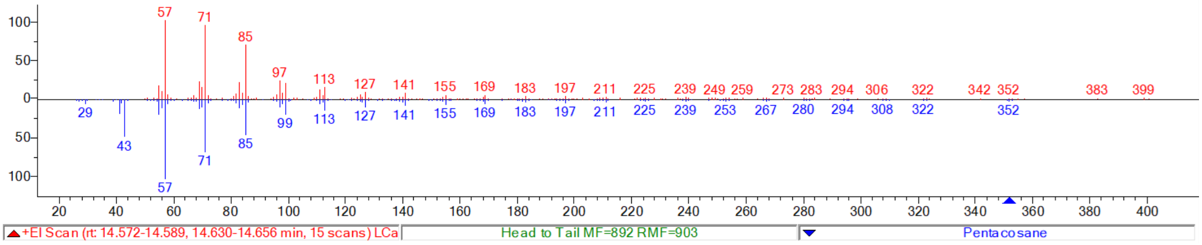
15.4	Heptacosane – Theoretical *mz* 380.4382 – Experimental *mz* 380.4366 – Error: -4.20 ppm 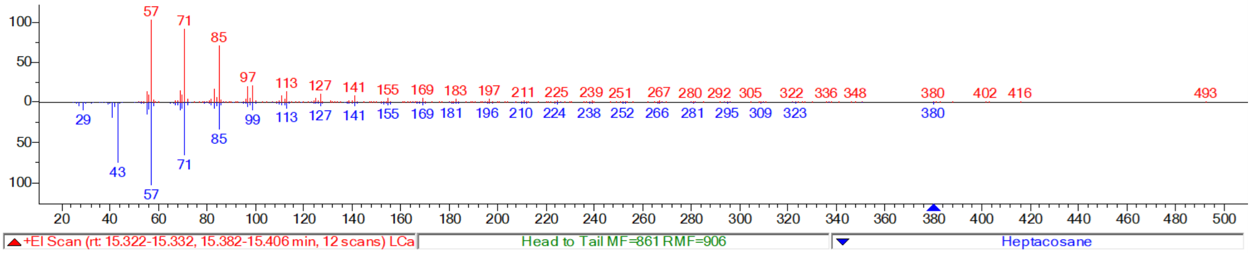

16.2	Nonacosane – Theoretical *mz* 408.4695 – Experimental *mz* 408.4683 – Error: -2.94 ppm<br>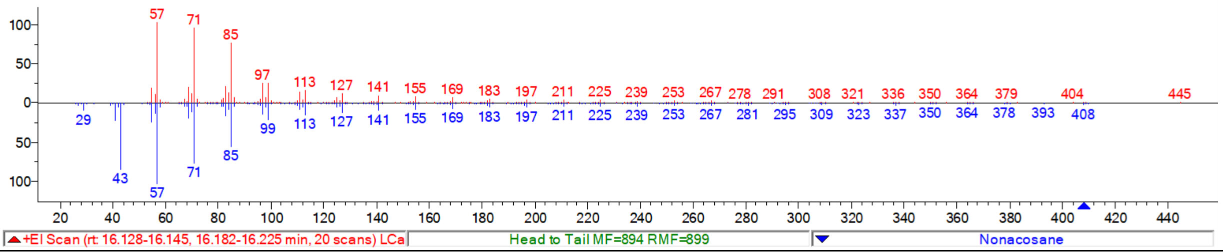
17.1	Hentriacontane – Theoretical *mz* 436.5008 – Experimental *mz* 436.5026 – Error: 4.12 ppm<br>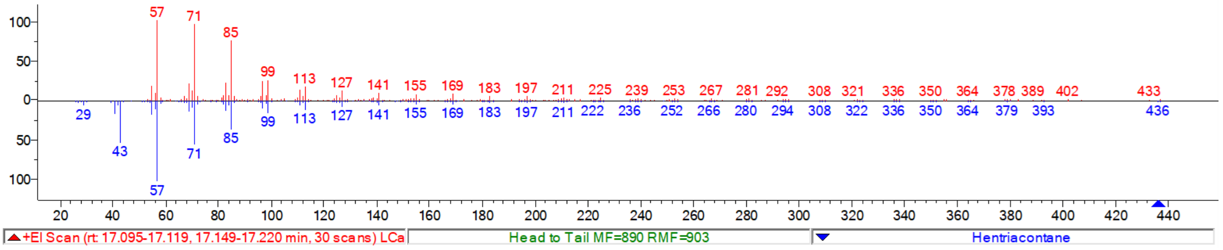

Table S 2 - Compounds identified in Modern Honey by TSP-GC/MS

RT (min)	Compound Matching<br>unknown in RED (top) and matched compound in BLUE (bottom)
12.4	Hexadecanoic acid – Theoretical *mz* 256.2402 – Experimental *mz* 256.2381 – Error: 5.28 ppm<br>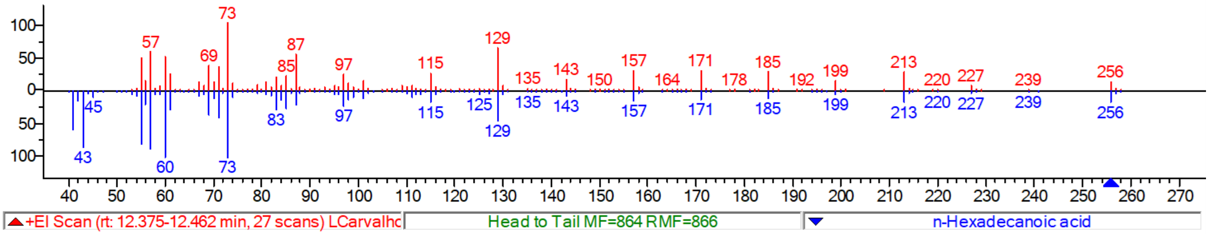
13.3	Octadecanoic acid – Theoretical *mz* 284.2715 – Experimental *mz* 284.2699 – Error: -5.73 ppm<br>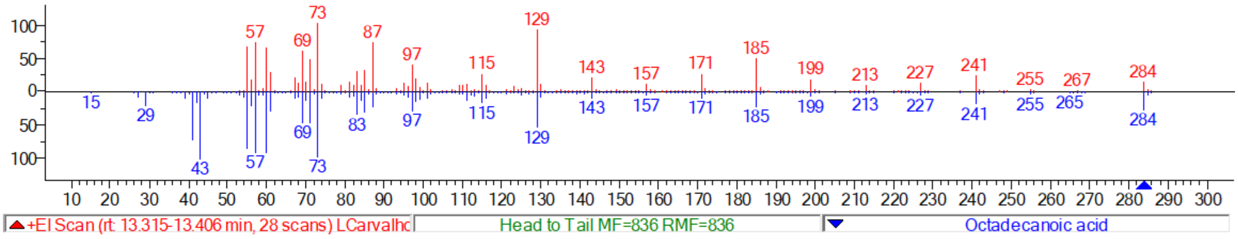

Table S 3 - Compounds identified in Fresh Honeycomb from Greece by TSP-GC/MS

RT (min)	Compound Matching unknown in RED (top) and matched compound in BLUE (bottom)
12.4	Hexadecanoic acid – Theoretical *mz* 256.2402 – Experimental *mz* 256.2438 – Error: -5.73 ppm 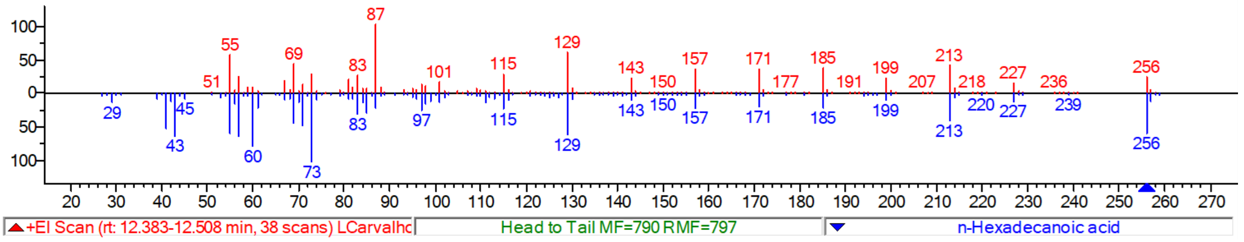
13.0	Heneicosane– Theoretical *mz* 296.3443 – Experimental *mz* 296.3454 – Error: 11.81 ppm 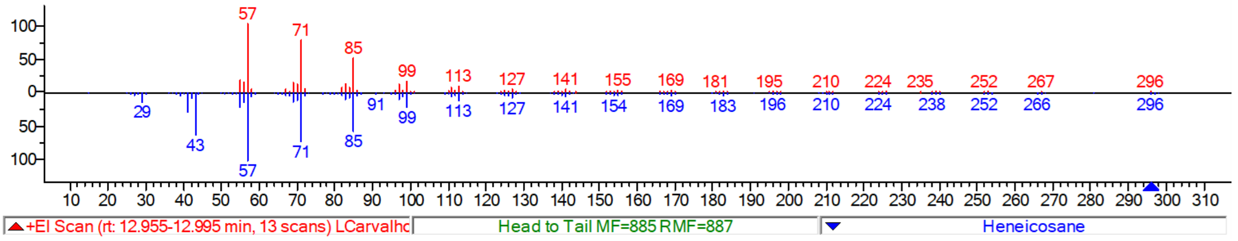
13.4	Octadecanoic acid – Theoretical *mz* 284.2715 – Experimental *mz* 284.2752 – Error: 11.81 ppm 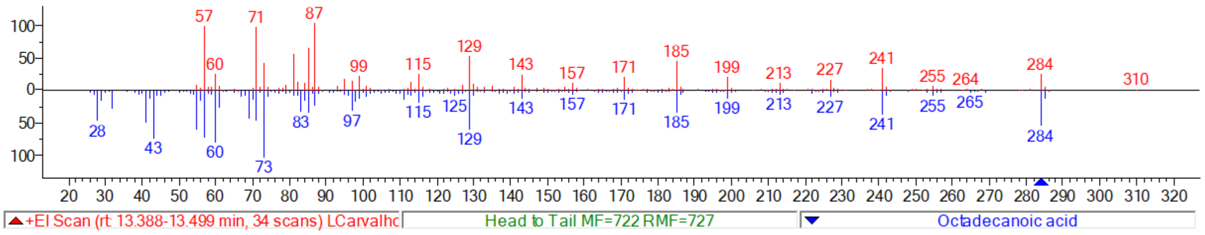
14.7	Pentacosane – Theoretical *mz* 352.4069 – Experimental *mz* 352.4116 – Error: 13.33 ppm 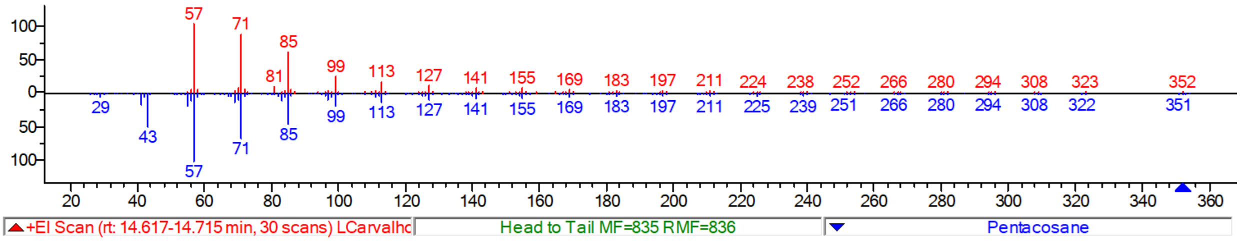
15.4	Heptacosane – Theoretical *mz* 380.4382 – Experimental *mz* 380.4470 – Error: 13.34 ppm 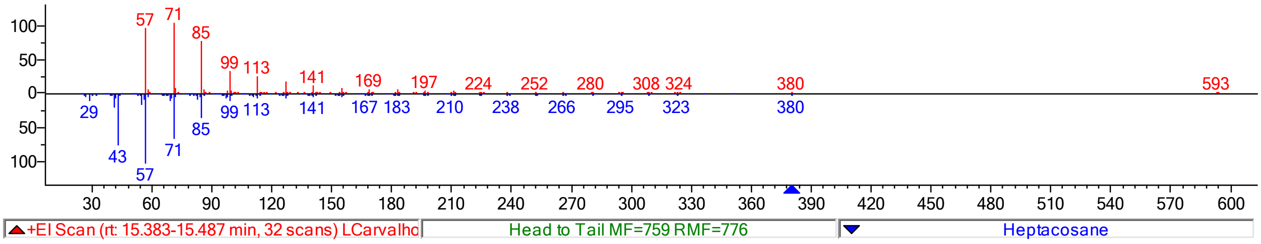

Table S 4 - Compounds identified in Aged Honeycomb from Greece by TSP-GC/MS

<table>
<tr><th>RT
(min)</th><th>Compound Matching
unknown in RED (top) and matched compound in BLUE (bottom)</th></tr>
<tr><td>12.4</td><td>Hexadecanoic acid – Theoretical mz 256.2402 – Experimental mz 256.2410 – Error: 3.12 ppm
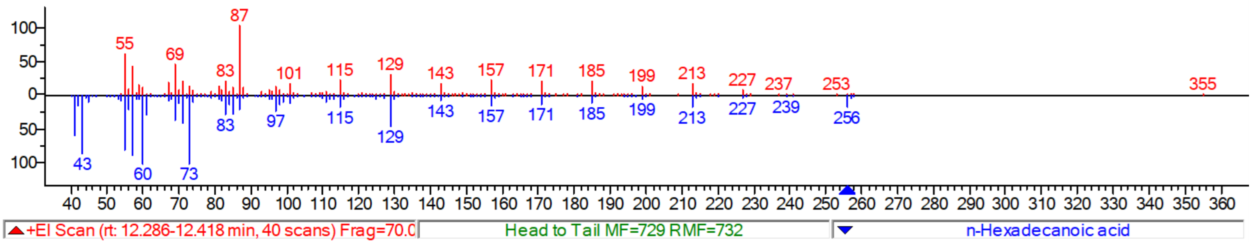</td></tr>
<tr><td>13.3</td><td>Octadecanoic acid – Theoretical mz 284.2715 – Experimental mz 284.2730 – Error: 5.28 ppm
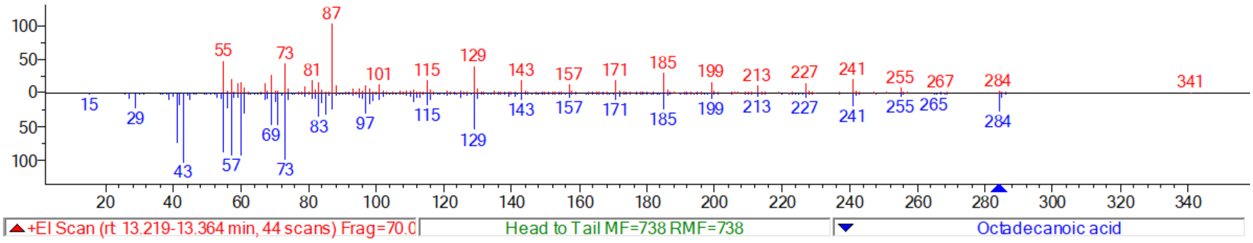</td></tr>
</table>

Table S 5 – Compounds identified in the Paestum residue by TSP-GC/MS analysis and corresponding Match Factor / Reserve Match Factor*

RT		PAESTUM RESIDUE			
(min)	Compound	Core	S. Orange	S. Black	S. Green
4.7	Hexanal	735 / 931			
5.6	Heptanal	727 / 793	746 / 806	642 / 796	801 / 841
6.2	5-methyl furfural			707 / 831	
7.0	Heptanoic acid			804 / 818	729 / 765
7.3	Nonanal		865 / 869		
7.4	Levoglucosenone			669 / 785	
7.8	Octanoic acid		744 / 829		680 / 819
8.5	Nonanoic acid			821 / 881	638 / 700
9.1	Decanoic acid			864 / 897	
9.4	Decenoic acid		839 / 855		
11.4	Tetradecanoic acid	673 / 767		737 / 825	652 / 705
12.4	Hexadecanoic acid	831 / 833	770 / 820	800 / 806	
13.1	Heptadecanoic acid	751 / 752			
13.4	Octadecanoic acid	778 / 782		709 / 714	
15.0	Hexadecanoic acid octyl ester	806 / 854			

* based on electron ionization fragmentation patterns using the NIST Library (Tables S6-9).
Note: Highlighted compounds are related to saccharides.

Table S 6 - Compounds identified in the Paestum Residue Core Sample by TSP-GC/MS

RT (min)	Compound Matching<br>unknown in RED (top) and matched compound in BLUE (bottom)
4.7	Hexanal – Theoretical *mz* 100.0894 – Experimental *mz* 100.0852 – Error: -42.0 ppm<br>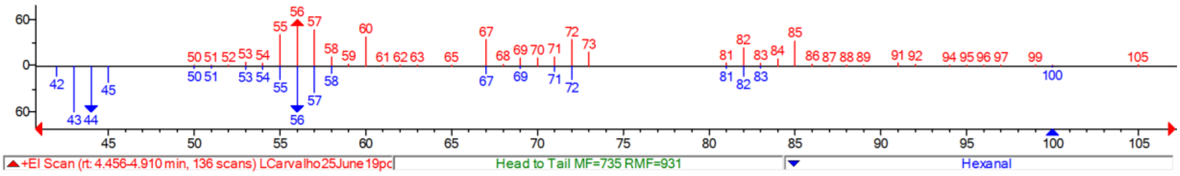
5.6	Heptanal – Theoretical *mz* 114.1050 – Experimental *mz* 114.1033 – Error: -14.9 ppm<br>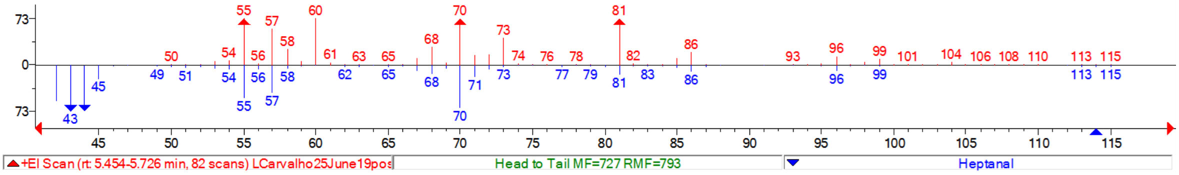
11.4	Tetradecanoic acid – Theoretical *mz* 228.2095 – Experimental *mz* 228.2054 – Error: -18.0 ppm<br>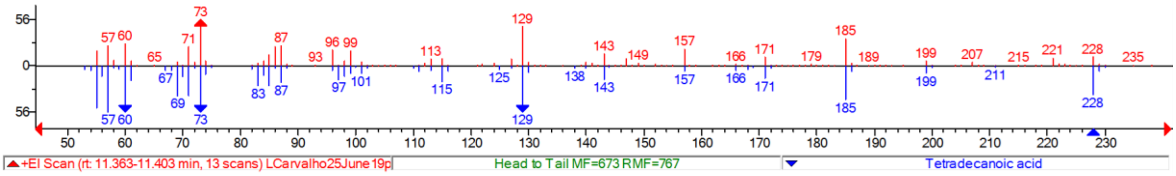
12.4	n-Hexadecanoic acid – Theoretical *mz* 256.2408 – Experimental *mz* 256.2414 – Error: 2.3 ppm<br>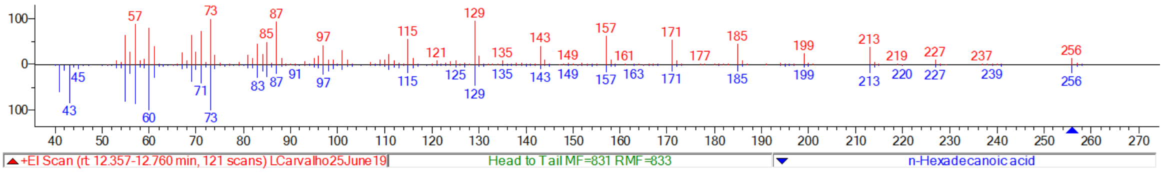
13.1	Heptadecanoic acid – Theoretical *mz* 270.2564 – Experimental *mz* 270.2560 – Error: -1.5 ppm<br>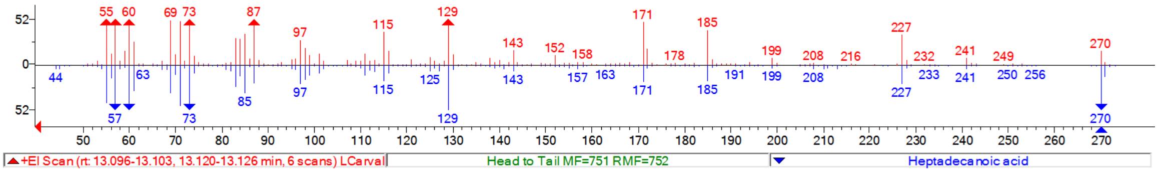
13.6	Octadecanoic acid – Theoretical *mz* 284.2715 – Experimental *mz* 284.2700 – Error: -5.28 ppm<br>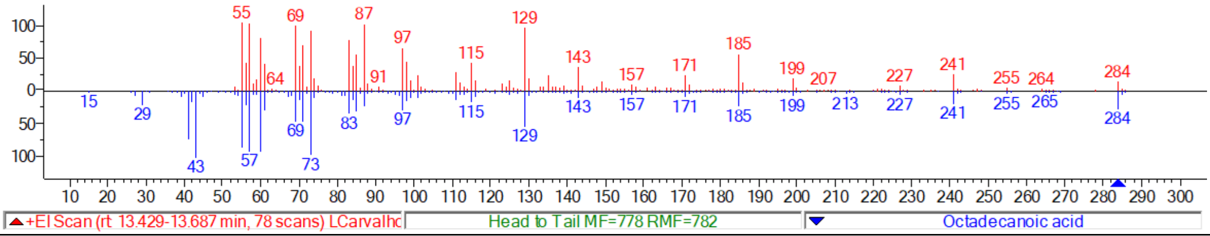
15.0	Hexadecanoic acid, octyl ester – Theoretical *mz* 368.3660 – Experimental *mz* 368.3726 – Error: 17.9 ppm<br>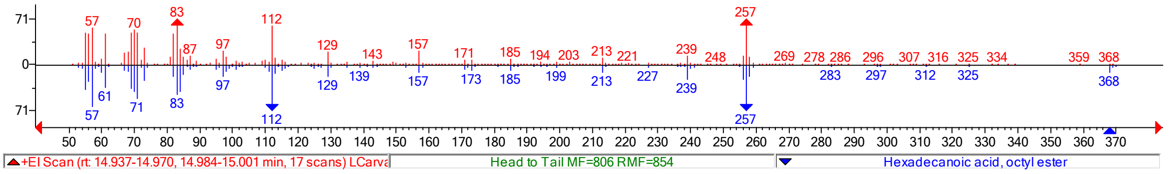

Table S 7 Compounds identified in the Paestum Residue Surface Orange-colored Sample by TSP-GC/MS

RT (Min)	Compound Matching<br>unknown in RED (top) and matched compound in BLUE (bottom)
5.6	Heptanal – Theoretical *mz* 114.1050 – Experimental *mz* 114.1000 – Error: -43.8 ppm<br>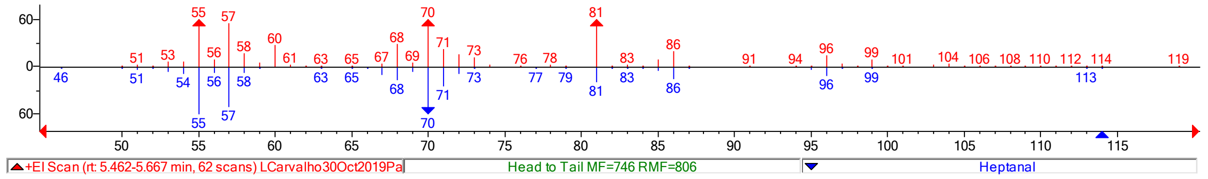
7.3	Nonanal – Theoretical *mz* 142.1363 – Experimental *mz* 142.1319 – Error: -31.0 ppm<br>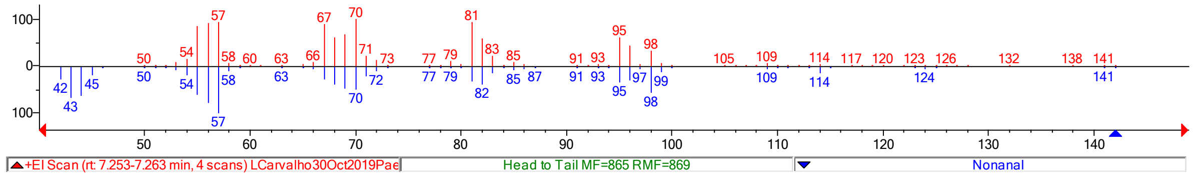
7.8	Octanoic acid – Theoretical *mz* 144.1156 – Experimental *mz* 144.1094 – Error: -43.0 ppm<br>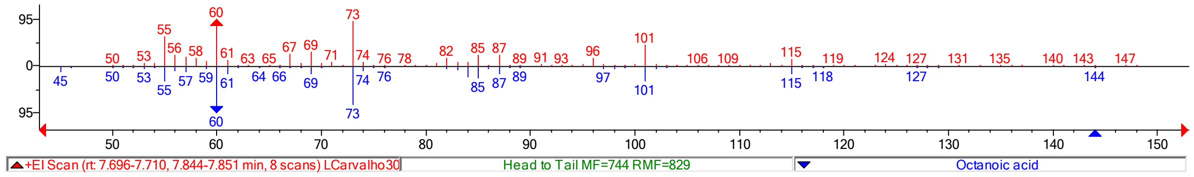
9.4	2-Decenoic acid – Theoretical *mz* 170.1312 – Experimental *mz* 170.0995 – Error: -34.3 ppm<br>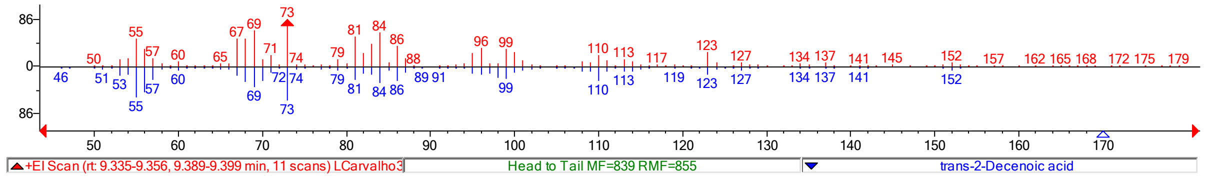
12.4	n-Hexadecanoic acid – Theoretical *mz* 256.2408 – Experimental *mz* 256.2381 – Error: -10.5 ppm<br>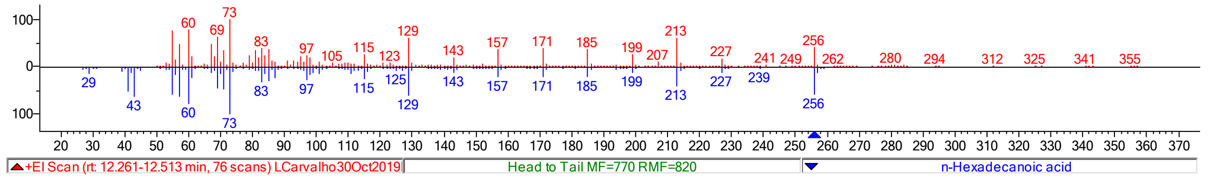

Table S 8 - Compounds identified in the Paestum Residue Surface Black-colored Sample by TSP-GC/MS

RT (Min)	Compound Matching<br>unknown in RED (top) and matched compound in BLUE (bottom)
5.6	Heptanal – Theoretical *mz* 114.1050 – Experimental *mz* 114.1010 – Error: -35.1 ppm<br>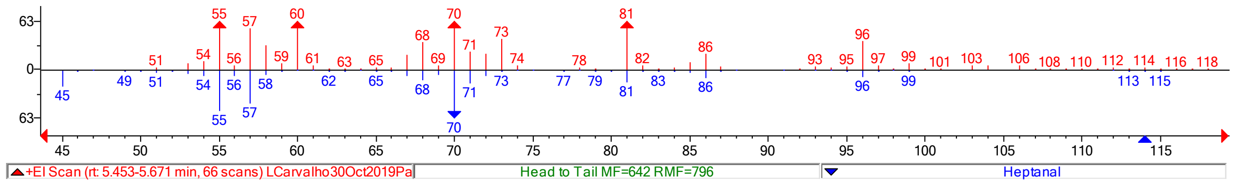
6.2	2-Furancarboxaldehyde, 5-methyl – Theoretical *mz* 110.0373 – Experimental *mz* 110.0359 – Error: -12.7 ppm<br>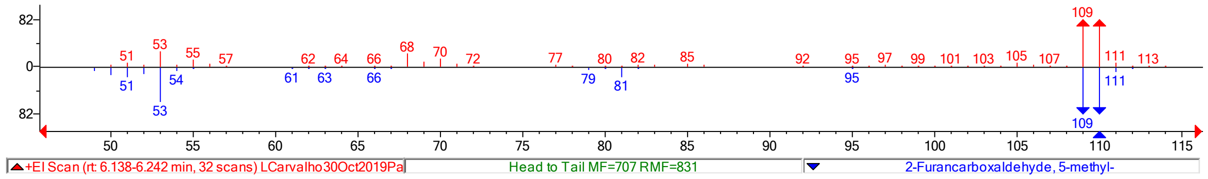
7.0	Heptanoic acid – Theoretical *mz* 130.0999 – Experimental *mz* 130.0933 – Error: -50.7 ppm<br>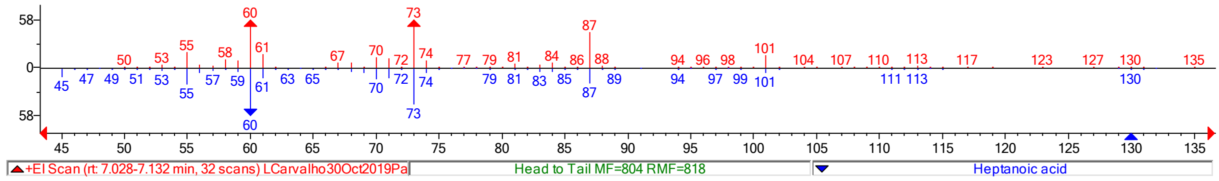
7.5	Levoglucosenone – Theoretical *mz* 126.0322 – Experimental *mz* 126.0322 – Error: ZERO<br>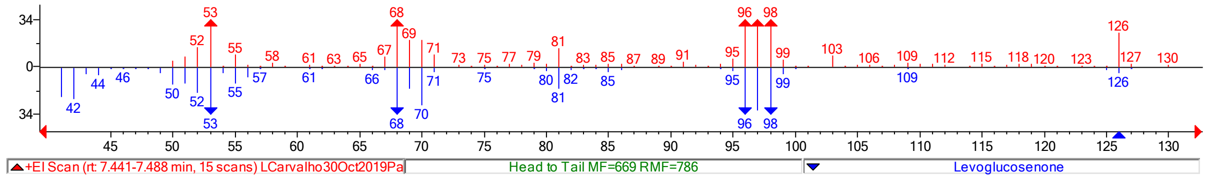
8.5	Nonanoic acid – Theoretical *mz* 158.1312 – Experimental *mz* 158.1286 – Error: -16.4 ppm<br>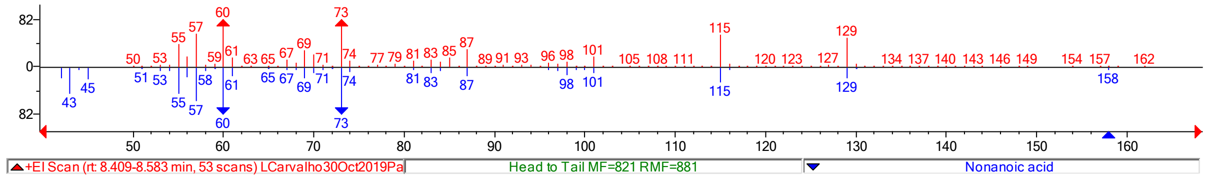
9.1	n-Decanoic acid – Theoretical *mz* 172.1469 – Experimental *mz* 172.1438 – Error: -18.0 ppm<br>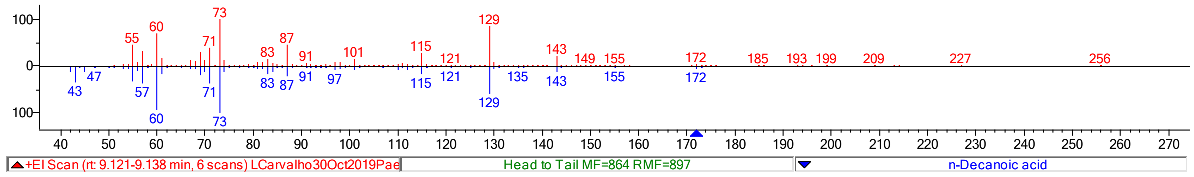
11.4	Tetradecanoic acid – Theoretical *mz* 228.2095 – Experimental *mz* 228.2076 – Error: -8.3 ppm<br>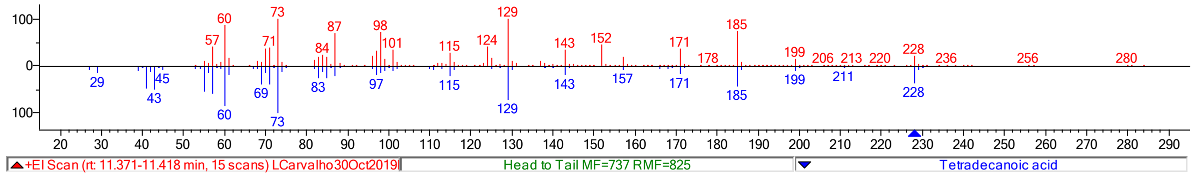

12.4	n-Hexadecanoic acid – Theoretical *mz* 256.2408 – Experimental *mz* 256.2394 – Error: -5.5 ppm 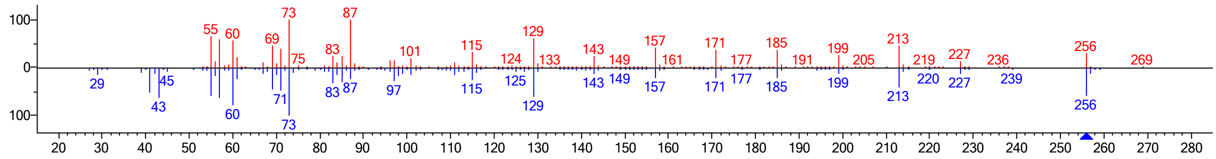
13.5	Octadecanoic acid – Theoretical *mz* 284.2707 – Experimental *mz* 284.2700 – Error: -2.92 ppm 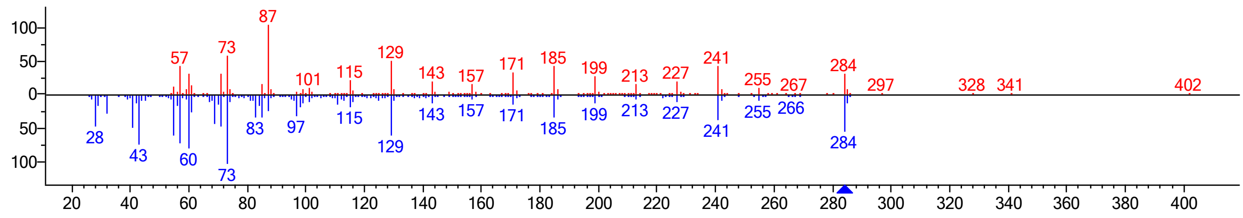

Table S 9 - Compounds identified in the Paestum Residue Surface Green-colored Sample by TSP-GC/MS

RT (Min)	Compound Matching<br>unknown in RED (top) and matched compound in BLUE (bottom)
5.6	Heptanal – Theoretical *mz* 114.1050 – Experimental *mz* 114.1050 – Error: -10.5 ppm 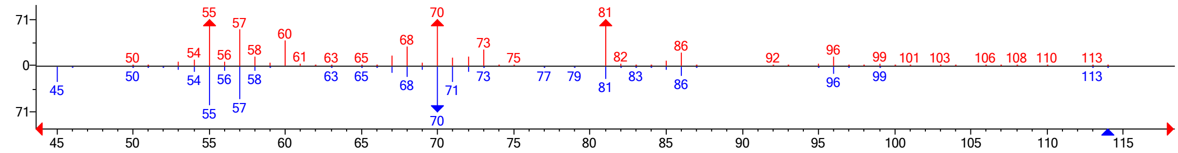
7.0	Heptanoic acid – Theoretical *mz* 130.0999 – Experimental *mz* 130.0963 – Error: -27.7 ppm 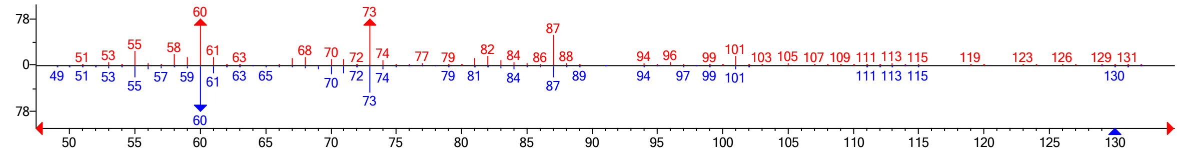
7.8	Octanoic acid – Theoretical *mz* 144.1156 – Experimental *mz* 144.1156 – Error: 2.8 ppm 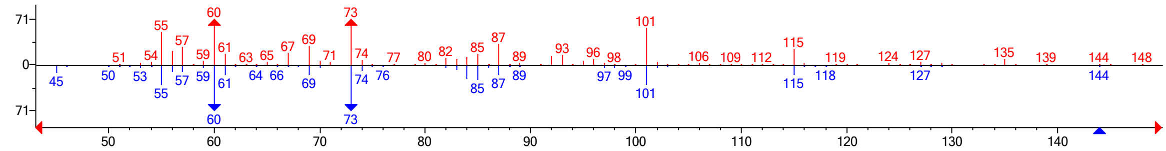
8.5	Nonanoic acid – Theoretical *mz* 158.1312 – Experimental *mz* 158.1310 – Error: -1.3 ppm 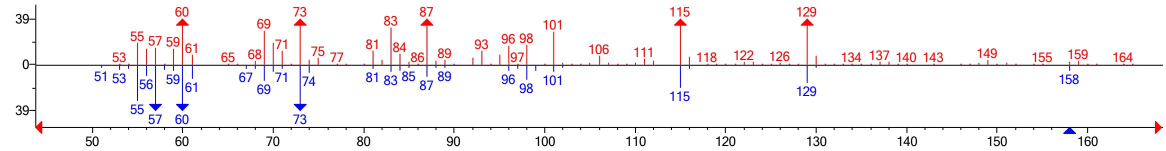
11.4	Tetradecanoic acid – Theoretical *mz* 228.2095 – Experimental *mz* 228.2143 – Error: 21.0 ppm 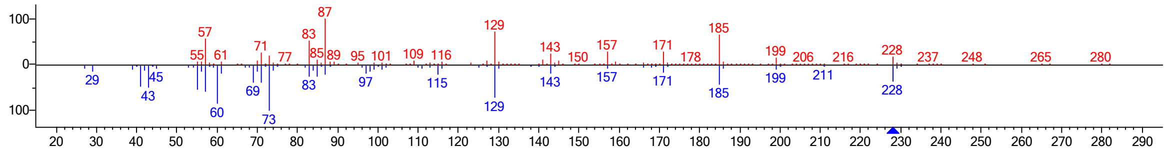

Table S 10 - Compounds identified in the methanol extract of the Paestum Residue's Core Sample by TSP-GC/MS

RT (min)	Compound Matching unknown in RED (top) and matched compound in BLUE (bottom
7.0	Heptanoic acid – Theoretical *mz* 130.0999 – Experimental *mz* 130.0910 – Error: - 64.4 ppm 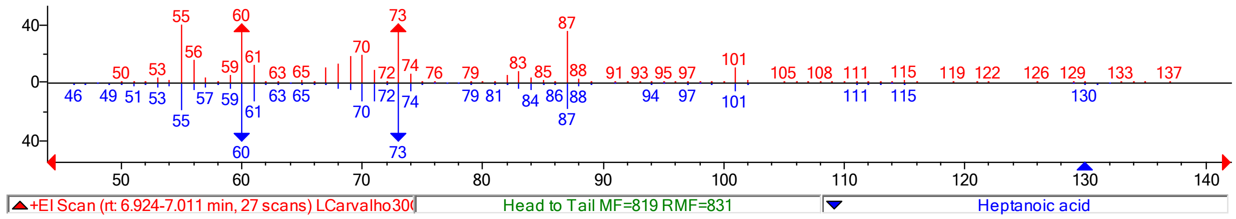
7.2	Nonanal – Theoretical *mz* 142.1363 – Experimental *mz* 142.1260 – Error: -68.70 ppm 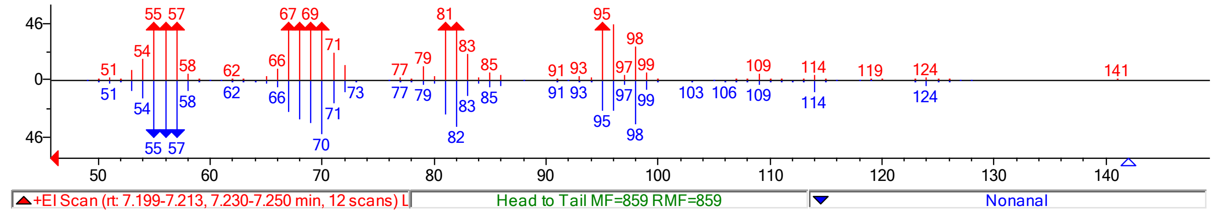
7.7	Octanoic acid – Theoretical *mz* 144.1156 – Experimental *mz* 144.1006 – Error: -100.12 ppm 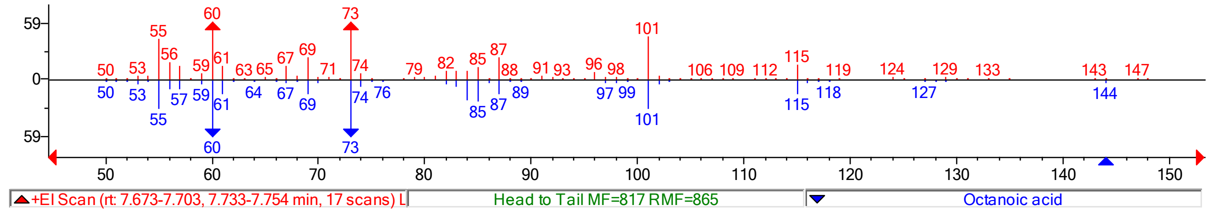
8.4	Nonanoic acid – Theoretical *mz* 158.1312 – Experimental *mz* 158.1286 – Error: -16.44 ppm 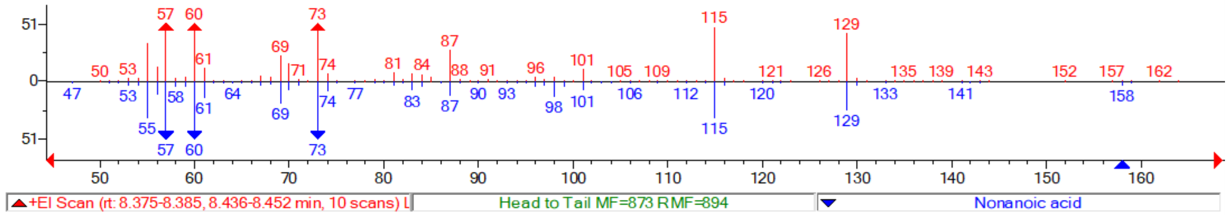
9.0	n-Decanoic acid – Theoretical *mz* 172.14633 – Experimental *mz* 172.1433 - Error: -17.60 ppm 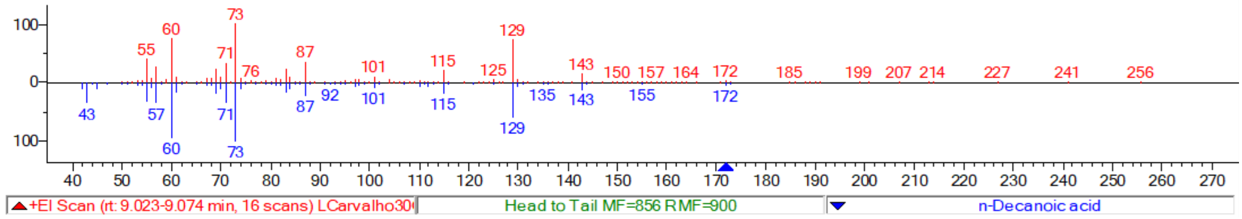
9.3	3-Decenoic acid - Theoretical *mz* 170.13068 – Experimental *mz* 170.1059 - Error: -145.65 ppm 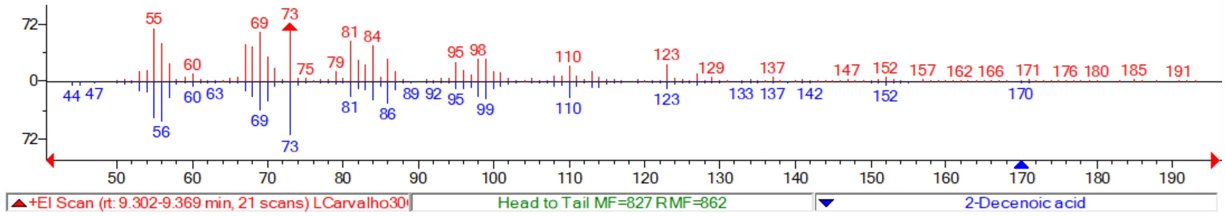

9.8	1-Dodecanol – Theoretical *mz* 186.198365 – Experimental *mz* 186.2316 – Error: 178.49 ppm 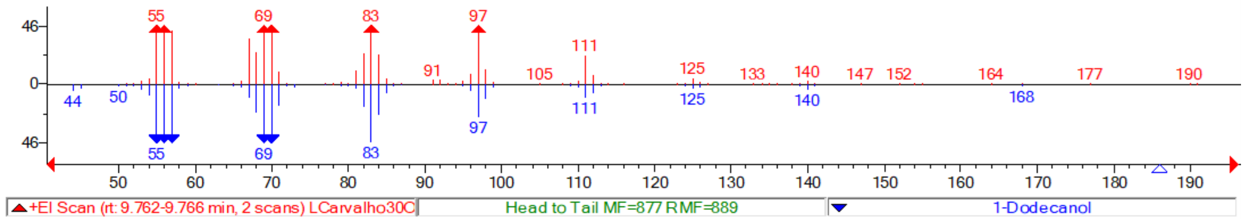
11.0	Azelaic acid – Theoretical *mz* 188.104859 – Experimental *mz* 188.1158 – Error: 58.16 ppm 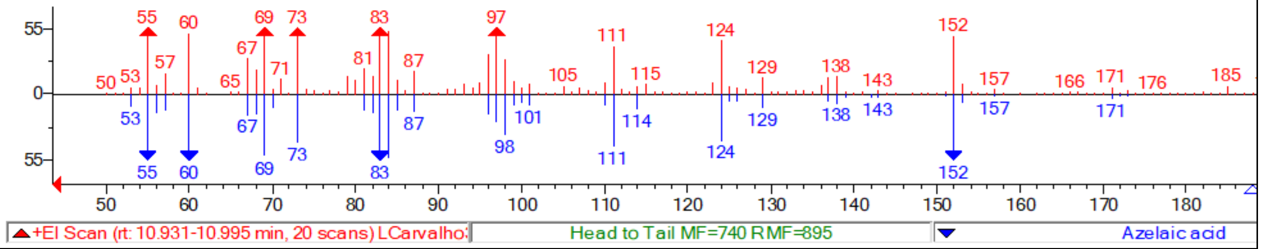
12.5	n-Hexadecanoic acid – Theoretical *mz* 256.2408 – Experimental *mz* 256.2399 – Error: -3.51 ppm 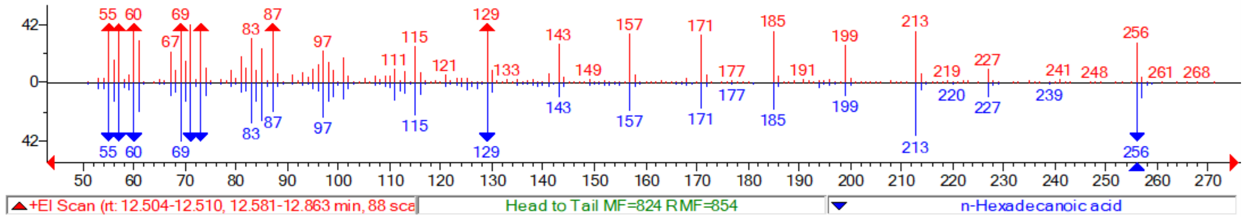
13.4	Octadecanoic acid – Theoretical *mz* 284.27153 – Experimental *mz* 284.2697 – Error: -6.44 ppm 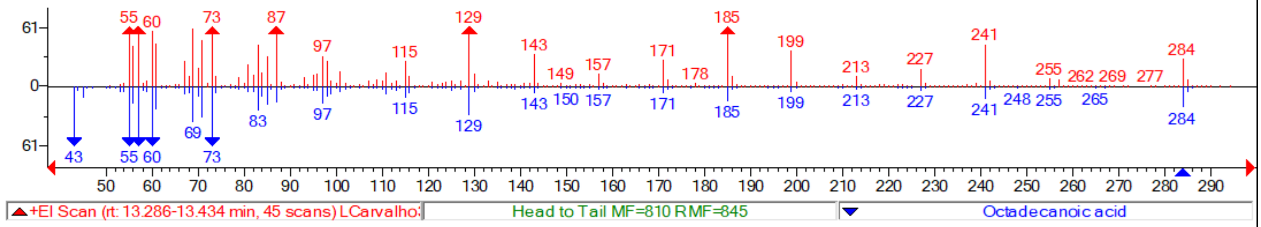

Table S 11 Compounds identified in the H2O extract of honey, beeswax and Paestum residue (core sample) by AEC-MS

M-H adduct m/z	RT (min)	Peak width (min)	Max Fold Change	Isotope Distribution	Raw abundance Honey	Beeswax	P. Residue	Accepted Description	Formula	Score	Mass Error (ppm)	Isotope Similarity	RT error (min)
179.0559	2.89	1.96	122.87	100 - 4.54 - 0.566	4879.79	40.08	442.48	Hexose Sugars	C6H12O6	39.6	-1.35	97.06	-0.54
165.0405	4.34	1.83	1.26	100	416.44	530.63	535.15	Arabinonic acid	C5H10O6	39.3	0.36	93.35	-0.56
124.0072	4.46	1.03	7.90	100	103.61	13.23	1542.77	Taurine	C2H7NO3S	38.5	-1.37	91.87	-1.07
117.0190	10.50	36.95	1.00	100	24766.18	25101.07	25356.39	Succinic acid	C4H6O4	39.1	-3.23	94.83	-0.98
159.0661	10.82	36.95	1.04	100	5778.35	6036.23	6782.04	3,3-Dimethylglutaric acid	C7H12O4	39.7	-1.25	91.78	0.47
143.0348	11.27	3.88	1.15	100	356.15	313.01	303.24	Dimethyl fumarate	C6H8O4	38.8	-1.34	92.81	-1.89
191.0196	16.13	0.88	1.52	100 - 2.31	949.25	628.80	1340.58	Citric acid	C6H8O7	39.9	-0.51	94.22	-1.15

Table S 12 Compounds identified in the H2O extracts of fresh and aged honeycombs by AEC-MS

M-H adduct *m/z*	RT (min)	Peak width (min)	Max Fold Change	Isotope Distribution	Raw abundance *Honeycomb Greece* Fresh	*Honeycomb Greece* Aged	*Honeycomb Italy* Fresh	*Honeycomb Italy* Aged	Accepted Description	Formula	Score	Mass Error (ppm)	Isotope Similarity	RT error (min)
177.0406	4.39	1.44	2.09	100 - 0.307	3386.81	1916.27	3787.95	1484.16	Gluconolactone	C6H10O6	47.3	0.90	92.65	-0.32
179.0563	4.39	1.18	3.69	100 - 1.63	1458.72	923.26	1604.34	355.99	Hexose Sugars	C6H12O6	38.9	0.82	93.85	0.96
193.0355	5.62	0.66	14.90	100 - 4.47	2445.69	2131.55	4028.19	221.40	Galacturonic acid	C6H10O7	46.1	0.48	96.19	-0.89
117.0195	11.89	0.79	Infinity	100	145.31	97.93	126.69	0.00	Succinic acid	C4H6O4	39.8	1.17	94.83	0.78
133.0144	11.89	1.01	9.20	100	188.95	146.97	442.23	39.36	Malic acid	C4H6O5	53	1.27	94.61	0.75
191.0201	17.27	0.31	Infinity	100	125.25	0.00	490.23	0.00	Citric acid	C6H8O7	41.2	1.89	92.09	0.36

Table S 13 - Protein matches obtained for Paestum Residue against Uniprot All Proteins Database

Accession	-10lgP	Coverage (%)	Area	#Peptides (#unique)	PTM	Avg. Mass	Description
P35527	331.01	97	5.22E+08	193 (184)	Carbamidomethylation; Oxidation (M); Deamidation (NQ)	62064	Keratin type I cytoskeletal 9 OS=Homo sapiens OX=9606 GN=KRT9 PE=1 SV=3
P04264	311.18	88	3.06E+06	196 (20)	Carbamidomethylation; Oxidation (M); Deamidation (NQ)	66039	Keratin type II cytoskeletal 1 OS=Homo sapiens OX=9606 GN=KRT1 PE=1 SV=6
P35908	288.6	88	4.87E+07	99 (52)	Carbamidomethylation; Oxidation (M); Deamidation (NQ)	65433	Keratin type II cytoskeletal 2 epidermal OS=Homo sapiens OX=9606 GN=KRT2 PE=1 SV=2
P00761	284.6	94	3.91E+08	127 (65)	Carbamidomethylation; Oxidation (M); Deamidation (NQ)	24409	Trypsin OS=Sus scrofa OX=9823 PE=1 SV=1
P13645	284.44	82	5.46E+07	95 (34)	Carbamidomethylation; Oxidation (M); Deamidation (NQ)	58827	Keratin type I cytoskeletal 10 OS=Homo sapiens OX=9606 GN=KRT10 PE=1 SV=6
Q86YZ3	253.76	23	7.22E+06	93 (91)	Deamidation (NQ)	282389	Hornerin OS=Homo sapiens OX=9606 GN=HRNR PE=1 SV=2
P13647	247.63	72	1.26E+07	70 (11)	Carbamidomethylation; Oxidation (M); Deamidation (NQ)	62378	Keratin type II cytoskeletal 5 OS=Homo sapiens OX=9606 GN=KRT5 PE=1 SV=3
P04259	239.62	74	7.07E+05	64 (3)	Carbamidomethylation; Oxidation (M); Deamidation (NQ)	60067	Keratin type II cytoskeletal 6B OS=Homo sapiens OX=9606 GN=KRT6B PE=1 SV=5
P15924	239.17	25	5.09E+06	79 (71)	Carbamidomethylation; Oxidation (M); Deamidation (NQ)	331774	Desmoplakin OS=Homo sapiens OX=9606 GN=DSP PE=1 SV=3
Q6EIZ0	237.86	37	7.89E+06	55 (2)	Oxidation (M); Deamidation (NQ)	57711	Keratin type I cytoskeletal 10 OS=Canis lupus familiaris OX=9615 GN=KRT10 PE=2 SV=1
O43790	237.5	74	2.21E+05	44 (3)	Carbamidomethylation; Oxidation (M)	53501	Keratin type II cuticular Hb6 OS=Homo sapiens OX=9606 GN=KRT86 PE=1 SV=1
P02533	236.97	68	8.40E+06	51 (7)	Carbamidomethylation; Oxidation (M); Deamidation (NQ)	51562	Keratin type I cytoskeletal 14 OS=Homo sapiens OX=9606 GN=KRT14 PE=1 SV=4
P08779	232.1	74	7.63E+06	54 (24)	Carbamidomethylation; Oxidation (M); Deamidation (NQ)	51268	Keratin type I cytoskeletal 16 OS=Homo sapiens OX=9606 GN=KRT16 PE=1 SV=4
Q14533	226.07	64	1.49E+05	41 (2)	Carbamidomethylation; Oxidation (M)	54928	Keratin type II cuticular Hb1 OS=Homo sapiens OX=9606 GN=KRT81 PE=1 SV=3
Q02SZ7	221.75	51	2.94E+07	34 (34)	Carbamidomethylation; Deamidation (NQ)	48178	Lysyl endopeptidase OS=Pseudomonas aeruginosa (strain UCBPP-PA14) OX=208963 GN=prpL PE=1 SV=1
P02788	215.51	38	6.18E+05	26 (24)	Carbamidomethylation	78182	Lactotransferrin OS=Homo sapiens OX=9606 GN=LTF PE=1 SV=6
Q04695	214.89	65	1.07E+06	41 (5)	Carbamidomethylation; Oxidation (M); Deamidation (NQ)	48106	Keratin type I cytoskeletal 17 OS=Homo sapiens OX=9606 GN=KRT17 PE=1 SV=2
P78386	208.14	63	3.59E+05	40 (5)	Carbamidomethylation; Oxidation (M)	55802	Keratin type II cuticular Hb5 OS=Homo sapiens OX=9606 GN=KRT85 PE=1 SV=1
Q8NHM4	205.18	38	7.69E+05	37 (2)	Carbamidomethylation; Deamidation (NQ)	26537	Putative trypsin-6 OS=Homo sapiens OX=9606 GN=PRSS3P2 PE=5 SV=2

Accession	-10lgP	Coverage (%)	Area	#Peptides (#unique)	PTM	Avg. Mass	Description
Q15323	201.35	74	6.84E+05	32 (5)	Carbamidomethylation; Oxidation (M); Deamidation (NQ)	47237	Keratin type I cuticular Ha1 OS=Homo sapiens OX=9606 GN=KRT31 PE=1 SV=3
P02768	196.2	47	4.38E+06	34 (21)	Carbamidomethylation	69367	Serum albumin OS=Homo sapiens OX=9606 GN=ALB PE=1 SV=2
Q14525	191.96	67	4.58E+05	27 (4)	Carbamidomethylation; Oxidation (M); Deamidation (NQ)	46214	Keratin type I cuticular Ha3-II OS=Homo sapiens OX=9606 GN=KRT33B PE=1 SV=3
Q6IG02	178.2	30	1.40E+06	33 (5)	Carbamidomethylation; Oxidation (M); Deamidation (NQ)	69127	Keratin type II cytoskeletal 2 epidermal OS=Rattus norvegicus OX=10116 GN=Krt2 PE=3 SV=1
Q6EIZ1	178.14	29	1.85E+04	35 (2)	Carbamidomethylation; Oxidation (M); Deamidation (NQ)	64575	Keratin type II cytoskeletal 2 epidermal OS=Canis lupus familiaris OX=9615 GN=KRT2 PE=2 SV=1
P19013	176.94	42	1.11E+06	28 (9)	Oxidation (M)	56144	Keratin type II cytoskeletal 4 OS=Homo sapiens OX=9606 GN=KRT4 PE=1 SV=5
P0AEX9	176.16	43	2.74E+06	26 (8)	Oxidation (M); Deamidation (NQ)	43388	Maltose/maltodextrin-binding periplasmic protein OS=Escherichia coli (strain K12) OX=83333 GN=malE PE=1 SV=1
P0AEY0	176.16	43	2.74E+06	26 (8)	Oxidation (M); Deamidation (NQ)	43388	Maltose/maltodextrin-binding periplasmic protein OS=Escherichia coli O157:H7 OX=83334 GN=malE PE=1 SV=1
O76011	174.12	49	5.79E+05	21 (4)	Carbamidomethylation; Oxidation (M)	49424	Keratin type I cuticular Ha4 OS=Homo sapiens OX=9606 GN=KRT34 PE=1 SV=2
Q7Z794	164.2	26	2.96E+05	26 (6)	Oxidation (M); Deamidation (NQ)	61901	Keratin type II cytoskeletal 1b OS=Homo sapiens OX=9606 GN=KRT77 PE=2 SV=3
Q8N1N4	162.14	34	4.55E+05	22 (11)	Carbamidomethylation; Deamidation (NQ)	56866	Keratin type II cytoskeletal 78 OS=Homo sapiens OX=9606 GN=KRT78 PE=1 SV=2
Q02413	160.59	18	2.12E+06	15 (10)	Carbamidomethylation; Oxidation (M)	113748	Desmoglein-1 OS=Homo sapiens OX=9606 GN=DSG1 PE=1 SV=2
P23490	159.38	50	2.38E+05	14 (14)	Carbamidomethylation	25761	Loricrin OS=Homo sapiens OX=9606 GN=LORICRIN PE=1 SV=2
P08426	157.1	35	1.40E+07	23 (2)	Carbamidomethylation; Deamidation (NQ)	26269	Cationic trypsin-3 OS=Rattus norvegicus OX=10116 GN=Try3 PE=2 SV=1
P13646	153.44	29	4.87E+05	19 (3)	Carbamidomethylation; Oxidation (M); Deamidation (NQ)	49588	Keratin type I cytoskeletal 13 OS=Homo sapiens OX=9606 GN=KRT13 PE=1 SV=4
Q6IG01	152.91	19	2.72E+06	23 (3)	Oxidation (M); Deamidation (NQ)	57255	Keratin type II cytoskeletal 1b OS=Rattus norvegicus OX=10116 GN=Krt77 PE=3 SV=1
P00760	150.87	41	1.76E+06	23 (7)	Carbamidomethylation; Oxidation (M); Deamidation (NQ)	25785	Cationic trypsin OS=Bos taurus OX=9913 PE=1 SV=3
P19012	148.45	31	3.45E+04	20 (5)	Carbamidomethylation	49212	Keratin type I cytoskeletal 15 OS=Homo sapiens OX=9606 GN=KRT15 PE=1 SV=3
Q8WNW3	147.25	24	1.19E+06	18 (15)	Carbamidomethylation; Oxidation (M)	81850	Junction plakoglobin OS=Sus scrofa OX=9823 GN=Jup PE=2 SV=1
P01834	140.44	64	1.81E+06	5 (5)	Carbamidomethylation; Deamidation (NQ)	11765	Immunoglobulin kappa constant OS=Homo sapiens OX=9606 GN=IGKC PE=1 SV=2

Accession	-10lgP	Coverage (%)	Area	#Peptides (#unique)	PTM	Avg. Mass	Description
P81605	140.36	82	2.03E+07	23 (20)	Carbamidomethylation; Deamidation (NQ)	11284	Dermcidin OS=Homo sapiens OX=9606 GN=DCD PE=1 SV=2
Q08554	136.84	13	5.73E+05	9 (9)	Carbamidomethylation	99987	Desmocollin-1 OS=Homo sapiens OX=9606 GN=DSC1 PE=1 SV=2
P32765	135	36	1.79E+05	7 (7)	Carbamidomethylation	24039	21 kDa seed protein OS=Theobroma cacao OX=3641 GN=ASP PE=2 SV=1
P01876	130.9	25	1.99E+05	8 (5)	Carbamidomethylation	37655	Immunoglobulin heavy constant alpha 1 OS=Homo sapiens OX=9606 GN=IGHA1 PE=1 SV=2
P01040	129.01	74	3.70E+06	8 (8)		11006	Cystatin-A OS=Homo sapiens OX=9606 GN=CSTA PE=1 SV=1
O18330	128.64	23	7.99E+05	8 (8)	Oxidation (M)	48886	Major royal jelly protein 1 OS=Apis mellifera OX=7460 GN=MRJP1 PE=1 SV=1
P0DOX5	127.59	26	9.49E+05	9 (8)	Carbamidomethylation; Deamidation (NQ)	49329	Immunoglobulin gamma-1 heavy chain OS=Homo sapiens OX=9606 PE=1 SV=2
P01857	127.59	35	9.49E+05	9 (8)	Carbamidomethylation; Deamidation (NQ)	36106	Immunoglobulin heavy constant gamma 1 OS=Homo sapiens OX=9606 GN=IGHG1 PE=1 SV=1
Q6UWP8	126.83	20	2.36E+05	11 (11)		60541	Suprabasin OS=Homo sapiens OX=9606 GN=SBSN PE=1 SV=2
P12273	124.32	54	2.97E+05	6 (6)	Carbamidomethylation	16572	Prolactin-inducible protein OS=Homo sapiens OX=9606 GN=PIP PE=1 SV=1
Q13835	123.98	10	5.78E+04	7 (2)		82861	Plakophilin-1 OS=Homo sapiens OX=9606 GN=PKP1 PE=1 SV=2
P05109	121.98	69	3.40E+05	9 (7)	Carbamidomethylation	10835	Protein S100-A8 OS=Homo sapiens OX=9606 GN=S100A8 PE=1 SV=1
Q17060	121.15	17	4.13E+05	8 (7)	Oxidation (M)	61662	Major royal jelly protein 3 OS=Apis mellifera OX=7460 GN=MRJP3 PE=1 SV=1
P00763	120.47	37	6.56E+05	19 (2)	Carbamidomethylation; Oxidation (M); Deamidation (NQ)	26228	Anionic trypsin-2 OS=Rattus norvegicus OX=10116 GN=Prss2 PE=1 SV=2
P04406	117.15	29	4.07E+05	10 (10)	Carbamidomethylation; Deamidation (NQ)	36053	Glyceraldehyde-3-phosphate dehydrogenase OS=Homo sapiens OX=9606 GN=GAPDH PE=1 SV=3
Q9NZT1	116.35	42	1.66E+06	5 (5)	Deamidation (NQ)	15893	Calmodulin-like protein 5 OS=Homo sapiens OX=9606 GN=CALML5 PE=1 SV=2
P04745	115.82	15	6.62E+04	5 (5)		57768	Alpha-amylase 1 OS=Homo sapiens OX=9606 GN=AMY1A PE=1 SV=2
P61626	114.08	58	9.06E+05	10 (4)	Carbamidomethylation; Oxidation (M)	16537	Lysozyme C OS=Homo sapiens OX=9606 GN=LYZ PE=1 SV=1
P61627	114.08	58	9.06E+05	10 (4)	Carbamidomethylation; Oxidation (M)	16537	Lysozyme C OS=Pan paniscus OX=9597 GN=LYZ PE=3 SV=1
P61628	114.08	58	9.06E+05	10 (4)	Carbamidomethylation; Oxidation (M)	16537	Lysozyme C OS=Pan troglodytes OX=9598 GN=LYZ PE=2 SV=1

Accession	-10lgP	Coverage (%)	Area	#Peptides (#unique)	PTM	Avg. Mass	Description
P79179	114.08	58	9.06E+05	10 (4)	Carbamidomethylation; Oxidation (M)	16567	Lysozyme C OS=Gorilla gorilla gorilla OX=9595 GN=LYZ PE=2 SV=1
P91754	112.77	19	2.45E+05	6 (3)		41323	Actin (Fragment) OS=Lumbricus rubellus OX=35632 PE=2 SV=1
P10365	112.77	19	2.45E+05	6 (3)		41635	Actin OS=Thermomyces lanuginosus OX=5541 PE=3 SV=1
P41341	112.77	19	2.45E+05	6 (3)		41789	Actin-11 OS=Limulus polyphemus OX=6850 PE=2 SV=1
P53463	112.77	19	2.45E+05	6 (3)		41748	Actin cytoskeletal OS=Heliocidaris erythrogramma OX=7634 PE=3 SV=1
P17126	112.77	19	2.45E+05	6 (3)		41797	Actin non-muscle 6.2 OS=Hydra vulgaris OX=6087 PE=3 SV=1
Q26065	112.77	19	2.45E+05	6 (3)		41762	Actin adductor muscle OS=Placopecten magellanicus OX=6577 PE=2 SV=1
P53464	112.77	19	2.45E+05	6 (3)		41762	Actin cytoskeletal OS=Heliocidaris tuberculata OX=7635 PE=3 SV=1
P12717	112.77	19	2.45E+05	6 (3)		41952	Actin muscle OS=Pisaster ochraceus OX=7612 PE=3 SV=1
P02578	112.77	19	2.45E+05	6 (3)		41676	Actin-1 OS=Acanthamoeba castellanii OX=5755 PE=1 SV=1
P02576	112.77	19	2.45E+05	6 (3)		41800	Actin plasmodial isoform OS=Physarum polycephalum OX=5791 GN=ARDA PE=1 SV=2
P49871	112.77	19	2.45E+05	6 (3)		41777	Actin muscle OS=Manduca sexta OX=7130 PE=2 SV=1
P90689	112.77	19	2.45E+05	6 (3)		41710	Actin OS=Brugia malayi OX=6279 PE=1 SV=1
P07830	112.77	19	2.45E+05	6 (3)		41733	Major actin OS=Dictyostelium discoideum OX=44689 GN=act1 PE=1 SV=2
O16808	112.77	19	2.45E+05	6 (3)		41817	Actin OS=Mayetiola destructor OX=39758 PE=2 SV=1
Q54GX7	112.77	19	2.45E+05	6 (3)		41747	Actin-10 OS=Dictyostelium discoideum OX=44689 GN=act10 PE=1 SV=1
Q9NSB2	110.69	13	3.05E+05	13 (2)	Carbamidomethylation; Oxidation (M)	64842	Keratin type II cuticular Hb4 OS=Homo sapiens OX=9606 GN=KRT84 PE=2 SV=2
P04280	110.63	31	5.85E+05	12 (8)		38562	Basic salivary proline-rich protein 1 OS=Homo sapiens OX=9606 GN=PRB1 PE=1 SV=3
O76015	108.41	15	2.96E+04	11 (4)	Oxidation (M)	50480	Keratin type I cuticular Ha8 OS=Homo sapiens OX=9606 GN=KRT38 PE=1 SV=3
O77061	104.98	13	7.23E+04	4 (3)	Oxidation (M)	51074	Major royal jelly protein 2 OS=Apis mellifera OX=7460 GN=MRJP2 PE=1 SV=1

Accession	-10lgP	Coverage (%)	Area	#Peptides (#unique)	PTM	Avg. Mass	Description
P10163	103.98	28	7.28E+05	12 (6)		31326	Basic salivary proline-rich protein 4 OS=Homo sapiens OX=9606 GN=PRB4 PE=1 SV=4
Q6KB66	101.52	10	3.75E+04	8 (2)		50525	Keratin type II cytoskeletal 80 OS=Homo sapiens OX=9606 GN=KRT80 PE=1 SV=2
Q6TEQ7	97.94	14	2.22E+05	4 (4)		38654	Annexin A2 OS=Canis lupus familiaris OX=9615 GN=ANXA2 PE=1 SV=1
P07356	97.94	14	2.22E+05	4 (4)		38676	Annexin A2 OS=Mus musculus OX=10090 GN=Anxa2 PE=1 SV=2
P19620	97.94	14	2.22E+05	4 (4)		38542	Annexin A2 OS=Sus scrofa OX=9823 GN=ANXA2 PE=1 SV=4
Q5R5A0	97.94	14	2.22E+05	4 (4)		38604	Annexin A2 OS=Pongo abelii OX=9601 GN=ANXA2 PE=2 SV=1
P07355	97.94	14	2.22E+05	4 (4)		38604	Annexin A2 OS=Homo sapiens OX=9606 GN=ANXA2 PE=1 SV=2
C0HJG9	97.94	24	2.22E+05	4 (4)		22447	Annexin A2 (Fragments) OS=Mesocricetus auratus OX=10036 PE=1 SV=1
Q04118	97.61	36	7.52E+05	12 (10)		30980	Basic salivary proline-rich protein 3 OS=Homo sapiens OX=9606 GN=PRB3 PE=1 SV=2
Q7YR44	96.84	14	5.90E+05	6 (6)	Carbamidomethylation	51611	Corneodesmosin OS=Pan troglodytes OX=9598 GN=CDSN PE=2 SV=1
P31025	94.51	34	2.05E+05	5 (5)	Carbamidomethylation; Deamidation (NQ)	19250	Lipocalin-1 OS=Homo sapiens OX=9606 GN=LCN1 PE=1 SV=1
P31151	93.45	37	1.19E+05	4 (4)	Carbamidomethylation	11471	Protein S100-A7 OS=Homo sapiens OX=9606 GN=S100A7 PE=1 SV=4
Q96DA0	92.96	21	8.87E+04	4 (4)		22739	Zymogen granule protein 16 homolog B OS=Homo sapiens OX=9606 GN=ZG16B PE=1 SV=3
P31944	92.58	24	2.16E+05	6 (5)	Carbamidomethylation	27680	Caspase-14 OS=Homo sapiens OX=9606 GN=CASP14 PE=1 SV=2
P02662	92.02	28	4.70E+05	5 (5)		24529	Alpha-S1-casein OS=Bos taurus OX=9913 GN=CSN1S1 PE=1 SV=2
Q43358	91.58	14	5.72E+04	7 (7)	Carbamidomethylation	60798	Vicilin OS=Theobroma cacao OX=3641 GN=CSV PE=2 SV=1
P02754	91.55	30	7.05E+05	4 (4)	Carbamidomethylation	19883	Beta-lactoglobulin OS=Bos taurus OX=9913 GN=LGB PE=1 SV=3
Q01469	89.91	30	4.11E+05	6 (5)	Carbamidomethylation	15164	Fatty acid-binding protein 5 OS=Homo sapiens OX=9606 GN=FABP5 PE=1 SV=3
P05089	83.89	12	2.36E+05	5 (5)		34735	Arginase-1 OS=Homo sapiens OX=9606 GN=ARG1 PE=1 SV=2
P25311	81.18	20	3.13E+06	6 (6)		34259	Zinc-alpha-2-glycoprotein OS=Homo sapiens OX=9606 GN=AZGP1 PE=1 SV=2

Accession	-10lgP	Coverage (%)	Area	#Peptides (#unique)	PTM	Avg. Mass	Description
P06702	79.38	44	8.49E+05	6 (5)	Oxidation (M)	13242	Protein S100-A9 OS=Homo sapiens OX=9606 GN=S100A9 PE=1 SV=1
P47929	76.74	18	6.80E+04	2 (2)		15075	Galectin-7 OS=Homo sapiens OX=9606 GN=LGALS7 PE=1 SV=2
P0A9L0	75.41	13	1.88E+05	2 (2)	Oxidation (M)	20853	FKBP-type peptidyl-prolyl cis-trans isomerase SlyD OS=Escherichia coli O6:H1 (strain CFT073 / ATCC 700928 / UPEC) OX=199310 GN=slyD PE=3 SV=1
P0A9L1	75.41	13	1.88E+05	2 (2)	Oxidation (M)	20853	FKBP-type peptidyl-prolyl cis-trans isomerase SlyD OS=Escherichia coli O157:H7 OX=83334 GN=slyD PE=3 SV=1
P0A9K9	75.41	13	1.88E+05	2 (2)	Oxidation (M)	20853	FKBP-type peptidyl-prolyl cis-trans isomerase SlyD OS=Escherichia coli (strain K12) OX=83333 GN=slyD PE=1 SV=1
P0A9L2	75.41	13	1.88E+05	2 (2)	Oxidation (M)	20853	FKBP-type peptidyl-prolyl cis-trans isomerase SlyD OS=Shigella flexneri OX=623 GN=slyD PE=3 SV=1
P03973	74.6	30	9.21E+04	4 (4)	Carbamidomethylation	14326	Antileukoproteinase OS=Homo sapiens OX=9606 GN=SLPI PE=1 SV=2
Q32I91	73.07	24	6.79E+05	3 (3)		18695	DNA protection during starvation protein OS=Shigella dysenteriae serotype 1 (strain Sd197) OX=300267 GN=dps PE=3 SV=3
B6I7W9	73.07	24	6.79E+05	3 (3)		18695	DNA protection during starvation protein OS=Escherichia coli (strain SE11) OX=409438 GN=dps PE=3 SV=1
B7UM08	73.07	24	6.79E+05	3 (3)		18695	DNA protection during starvation protein OS=Escherichia coli O127:H6 (strain E2348/69 / EPEC) OX=574521 GN=dps PE=3 SV=1
Q1REB2	73.07	24	6.79E+05	3 (3)		18695	DNA protection during starvation protein OS=Escherichia coli (strain UTI89 / UPEC) OX=364106 GN=dps PE=3 SV=3
Q0TJN6	73.07	24	6.79E+05	3 (3)		18695	DNA protection during starvation protein OS=Escherichia coli O6:K15:H31 (strain 536 / UPEC) OX=362663 GN=dps PE=3 SV=3
A7ZY70	73.07	24	6.79E+05	3 (3)		18695	DNA protection during starvation protein OS=Escherichia coli O9:H4 (strain HS) OX=331112 GN=dps PE=3 SV=1
B7M787	73.07	24	6.79E+05	3 (3)		18695	DNA protection during starvation protein OS=Escherichia coli O8 (strain IAI1) OX=585034 GN=dps PE=3 SV=1
B7NAB2	73.07	24	6.79E+05	3 (3)		18695	DNA protection during starvation protein OS=Escherichia coli O17:K52:H18 (strain UMN026 / ExPEC) OX=585056 GN=dps PE=3 SV=1
A7ZJM7	73.07	24	6.79E+05	3 (3)		18695	DNA protection during starvation protein OS=Escherichia coli O139:H28 (strain E24377A / ETEC) OX=331111 GN=dps PE=3 SV=1
B2TVB6	73.07	24	6.79E+05	3 (3)		18695	DNA protection during starvation protein OS=Shigella boydii serotype 18 (strain CDC 3083-94 / BS512) OX=344609 GN=dps PE=3 SV=1
B1X7E2	73.07	24	6.79E+05	3 (3)		18695	DNA protection during starvation protein OS=Escherichia coli (strain K12 / DH10B) OX=316385 GN=dps PE=3 SV=1
B5YSA4	73.07	24	6.79E+05	3 (3)		18695	DNA protection during starvation protein OS=Escherichia coli O157:H7 (strain EC4115 / EHEC) OX=444450 GN=dps PE=3 SV=1
B1IXF6	73.07	24	6.79E+05	3 (3)		18695	DNA protection during starvation protein OS=Escherichia coli (strain ATCC 8739 / DSM 1576 / Crooks) OX=481805 GN=dps PE=3 SV=1

Accession	-10lgP	Coverage (%)	Area	#Peptides (#unique)	PTM	Avg. Mass	Description
B7NNP4	73.07	24	6.79E+05	3 (3)		18695	DNA protection during starvation protein OS=Escherichia coli O7:K1 (strain IAI39 / ExPEC) OX=585057 GN=dps PE=3 SV=1
B7LC95	73.07	24	6.79E+05	3 (3)		18695	DNA protection during starvation protein OS=Escherichia coli (strain 55989 / EAEC) OX=585055 GN=dps PE=3 SV=1
C4ZXY4	73.07	24	6.79E+05	3 (3)		18695	DNA protection during starvation protein OS=Escherichia coli (strain K12 / MC4100 / BW2952) OX=595496 GN=dps PE=3 SV=1
Q6XZR0	73.07	24	6.79E+05	3 (3)		18695	DNA protection during starvation protein OS=Kluyvera cryocrescens OX=580 GN=dps PE=3 SV=1
B7LMB6	73.07	24	6.79E+05	3 (3)		18695	DNA protection during starvation protein OS=Escherichia fergusonii (strain ATCC 35469 / DSM 13698 / CDC 0568-73) OX=585054 GN=dps PE=3 SV=1
Q323Y1	73.07	24	6.79E+05	3 (3)		18695	DNA protection during starvation protein OS=Shigella boydii serotype 4 (strain Sb227) OX=300268 GN=dps PE=3 SV=3
B7MQR6	73.07	24	6.79E+05	3 (3)		18695	DNA protection during starvation protein OS=Escherichia coli O81 (strain ED1a) OX=585397 GN=dps PE=3 SV=1
P0ABT2	73.07	24	6.79E+05	3 (3)		18695	DNA protection during starvation protein OS=Escherichia coli (strain K12) OX=83333 GN=dps PE=1 SV=2
P0ABT3	73.07	24	6.79E+05	3 (3)		18695	DNA protection during starvation protein OS=Escherichia coli O157:H7 OX=83334 GN=dps PE=3 SV=2
P0ABT4	73.07	24	6.79E+05	3 (3)		18695	DNA protection during starvation protein OS=Shigella flexneri OX=623 GN=dps PE=3 SV=2
Q6Y1R6	73.07	24	6.79E+05	3 (3)		18722	DNA protection during starvation protein OS=Proteus hauseri OX=183417 GN=dps PE=3 SV=1
Q3Z3X3	73.07	24	6.79E+05	3 (3)		18695	DNA protection during starvation protein OS=Shigella sonnei (strain Ss046) OX=300269 GN=dps PE=3 SV=3
B7MGS0	73.07	24	6.79E+05	3 (3)		18695	DNA protection during starvation protein OS=Escherichia coli O45:K1 (strain S88 / ExPEC) OX=585035 GN=dps PE=3 SV=1
Q8FJM0	73.07	24	6.79E+05	3 (3)		18753	DNA protection during starvation protein OS=Escherichia coli O6:H1 (strain CFT073 / ATCC 700928 / UPEC) OX=199310 GN=dps PE=3 SV=4
B1LMA4	73.07	24	6.79E+05	3 (3)		18695	DNA protection during starvation protein OS=Escherichia coli (strain SMS-3-5 / SECEC) OX=439855 GN=dps PE=3 SV=1
P02666	70.74	14	4.20E+05	4 (4)		25107	Beta-casein OS=Bos taurus OX=9913 GN=CSN2 PE=1 SV=2
P32119	66.84	20	8.66E+03	3 (2)		21892	Peroxiredoxin-2 OS=Homo sapiens OX=9606 GN=PRDX2 PE=1 SV=5
Q2PFZ3	66.84	20	8.66E+03	3 (2)		21892	Peroxiredoxin-2 OS=Macaca fascicularis OX=9541 GN=PRDX2 PE=2 SV=3
Q8K3U7	66.84	20	8.66E+03	3 (2)		21813	Peroxiredoxin-2 OS=Cricetulus griseus OX=10029 GN=PRDX2 PE=2 SV=3
Q5RC63	66.84	22	8.66E+03	3 (2)		19430	Peroxiredoxin-2 OS=Pongo abelii OX=9601 GN=PRDX2 PE=2 SV=3

Accession	-10lgP	Coverage (%)	Area	#Peptides (#unique)	PTM	Avg. Mass	Description
Q9BYR0	66.45	10	2.19E+05	3 (2)	Carbamidomethylation	22535	Keratin-associated protein 4-7 OS=Homo sapiens OX=9606 GN=KRTAP4-7 PE=1 SV=2
Q9BQ66	66.45	10	2.19E+05	3 (2)	Carbamidomethylation	21407	Keratin-associated protein 4-12 OS=Homo sapiens OX=9606 GN=KRTAP4-12 PE=1 SV=1
Q9BYQ5	66.45	10	2.19E+05	3 (2)	Carbamidomethylation	21825	Keratin-associated protein 4-6 OS=Homo sapiens OX=9606 GN=KRTAP4-6 PE=2 SV=4
Q9BYR2	66.45	12	2.19E+05	3 (2)	Carbamidomethylation	19363	Keratin-associated protein 4-5 OS=Homo sapiens OX=9606 GN=KRTAP4-5 PE=1 SV=4
P35321	66.44	34	8.92E+04	3 (3)	Carbamidomethylation	9877	Cornifin-A OS=Homo sapiens OX=9606 GN=SPRR1A PE=1 SV=2
P35322	66.44	34	8.92E+04	3 (3)	Carbamidomethylation	9852	Cornifin OS=Macaca mulatta OX=9544 GN=SPRR1 PE=2 SV=1
P22528	66.44	34	8.92E+04	3 (3)	Carbamidomethylation	9888	Cornifin-B OS=Homo sapiens OX=9606 GN=SPRR1B PE=1 SV=2
Q9BYE4	65.62	30	1.00E+05	2 (2)	Carbamidomethylation	8158	Small proline-rich protein 2G OS=Homo sapiens OX=9606 GN=SPRR2G PE=3 SV=1
P22531	65.62	31	1.00E+05	2 (2)	Carbamidomethylation	7855	Small proline-rich protein 2E OS=Homo sapiens OX=9606 GN=SPRR2E PE=2 SV=2
P22532	65.62	31	1.00E+05	2 (2)	Carbamidomethylation	7905	Small proline-rich protein 2D OS=Homo sapiens OX=9606 GN=SPRR2D PE=2 SV=2
P35326	65.62	31	1.00E+05	2 (2)	Carbamidomethylation	7965	Small proline-rich protein 2A OS=Homo sapiens OX=9606 GN=SPRR2A PE=1 SV=1
P35325	65.62	31	1.00E+05	2 (2)	Carbamidomethylation	7975	Small proline-rich protein 2B OS=Homo sapiens OX=9606 GN=SPRR2B PE=2 SV=1
P59666	64.73	19	2.00E+05	2 (2)	Carbamidomethylation	10245	Neutrophil defensin 3 OS=Homo sapiens OX=9606 GN=DEFA3 PE=1 SV=1
Q5G863	64.73	19	2.00E+05	2 (2)	Carbamidomethylation	10196	Neutrophil defensin 1 OS=Pan troglodytes OX=9598 GN=DEFA1 PE=3 SV=1
P59665	64.73	19	2.00E+05	2 (2)	Carbamidomethylation	10201	Neutrophil defensin 1 OS=Homo sapiens OX=9606 GN=DEFA1 PE=1 SV=1
P10599	62.95	23	2.25E+05	2 (2)		11737	Thioredoxin OS=Homo sapiens OX=9606 GN=TXN PE=1 SV=3
Q5R9M3	62.95	23	2.25E+05	2 (2)		11885	Thioredoxin OS=Pongo abelii OX=9601 GN=TXN PE=3 SV=3
P0DOY3	62.95	27	2.18E+05	2 (2)		11266	Immunoglobulin lambda constant 3 OS=Homo sapiens OX=9606 GN=IGLC3 PE=1 SV=1
P0DOY2	62.95	27	2.18E+05	2 (2)		11294	Immunoglobulin lambda constant 2 OS=Homo sapiens OX=9606 GN=IGLC2 PE=1 SV=1
O75223	60.33	17	8.47E+04	3 (3)		21008	Gamma-glutamylcyclotransferase OS=Homo sapiens OX=9606 GN=GGCT PE=1 SV=1

Accession	-10lgP	Coverage (%)	Area	#Peptides (#unique)	PTM	Avg. Mass	Description
Q9BYT5	58.28	20	2.72E+05	3 (3)	Carbamidomethylation	12957	Keratin-associated protein 2-2 OS=Homo sapiens OX=9606 GN=KRTAP2-2 PE=2 SV=3
Q9BYR9	58.28	20	2.72E+05	3 (3)	Carbamidomethylation	13480	Keratin-associated protein 2-4 OS=Homo sapiens OX=9606 GN=KRTAP2-4 PE=1 SV=1
Q9BYU5	58.28	20	2.72E+05	3 (3)	Carbamidomethylation	13514	Keratin-associated protein 2-1 OS=Homo sapiens OX=9606 GN=KRTAP2-1 PE=2 SV=2
P0C7H8	58.28	20	2.72E+05	3 (3)	Carbamidomethylation	13480	Keratin-associated protein 2-3 OS=Homo sapiens OX=9606 GN=KRTAP2-3 PE=1 SV=2
P04792	57.48	20	1.31E+05	3 (3)		22783	Heat shock protein beta-1 OS=Homo sapiens OX=9606 GN=HSPB1 PE=1 SV=2
P84050	55.35	19	9.08E+04	2 (2)		11381	Histone H4 OS=Rhynchosciara americana OX=7186 GN=His4 PE=3 SV=2
P62779	55.35	19	9.08E+04	2 (2)		11369	Histone H4 OS=Pycnopodia helianthoides OX=7614 PE=3 SV=2
Q28DR4	55.35	19	9.08E+04	2 (2)		11367	Histone H4 OS=Xenopus tropicalis OX=8364 GN=TGas006m08.1 PE=3 SV=1
P62797	55.35	19	9.08E+04	2 (2)		11367	Histone H4 OS=Oncorhynchus mykiss OX=8022 PE=1 SV=2
P62783	55.35	19	9.08E+04	2 (2)		11369	Histone H4 OS=Strongylocentrotus purpuratus OX=7668 PE=3 SV=2
P62778	55.35	19	9.08E+04	2 (2)		11369	Histone H4 OS=Pisaster ochraceus OX=7612 PE=3 SV=2
P62777	55.35	19	9.08E+04	2 (2)		11369	Histone H4 OS=Pisaster brevispinus OX=7611 PE=3 SV=2
P62802	55.35	19	9.08E+04	2 (2)		11367	Histone H4 OS=Sus scrofa OX=9823 PE=1 SV=2
P62798	55.35	19	9.08E+04	2 (2)		11367	Histone H4 OS=Xenopus borealis OX=8354 PE=3 SV=2
P62794	55.35	19	9.08E+04	2 (2)		11367	Histone H4 OS=Urechis caupo OX=6431 PE=3 SV=2
Q5RCS7	55.35	19	9.08E+04	2 (2)		11367	Histone H4 OS=Pongo abelii OX=9601 PE=3 SV=1
P62795	55.35	19	9.08E+04	2 (2)		11367	Histone H4 OS=Platynereis dumerilii OX=6359 PE=3 SV=2
P27996	55.35	19	9.08E+04	2 (2)		11385	Histone H4 OS=Solaster stimpsoni OX=7598 PE=3 SV=2
P62780	55.35	19	9.08E+04	2 (2)		11369	Histone H4 OS=Paracentrotus lividus OX=7656 PE=3 SV=2
Q27765	55.35	19	9.08E+04	2 (2)		11349	Histone H4 OS=Styela plicata OX=7726 PE=3 SV=3

Accession	-10lgP	Coverage (%)	Area	#Peptides (#unique)	PTM	Avg. Mass	Description
P62796	55.35	19	9.08E+04	2 (2)		11367	Histone H4 OS=Oreochromis niloticus OX=8128 PE=3 SV=2
P83865	55.35	19	9.08E+04	2 (2)		11397	Histone H4 OS=Penaeus vannamei OX=6689 PE=1 SV=2
P62799	55.35	19	9.08E+04	2 (2)		11367	Histone H4 OS=Xenopus laevis OX=8355 PE=1 SV=2
Q6WV73	55.35	19	9.08E+04	2 (2)		11367	Histone H4 OS=Mytilus californianus OX=6549 PE=3 SV=3
Q43083	55.35	19	9.08E+04	2 (2)		11425	Histone H4 OS=Pyrenomonas salina OX=3034 PE=3 SV=3
P62781	55.35	19	9.08E+04	2 (2)		11369	Histone H4 OS=Psammechinus miliaris OX=7660 PE=1 SV=2
P84045	55.35	19	9.08E+04	2 (2)		11381	Histone H4 OS=Tigriopus californicus OX=6832 GN=His4 PE=3 SV=2
P62804	55.35	19	9.08E+04	2 (2)		11367	Histone H4 OS=Rattus norvegicus OX=10116 GN=H4c2 PE=1 SV=2
P91890	55.35	19	9.08E+04	2 (2)		11337	Histone H4 OS=Trichogramma cacaeciae OX=1667566 PE=3 SV=3
P02663	55.32	10	3.86E+05	2 (2)		26019	Alpha-S2-casein OS=Bos taurus OX=9913 GN=CSN1S2 PE=1 SV=2
Q9GZZ8	50.89	15	6.96E+03	2 (2)	Oxidation (M)	14246	Extracellular glycoprotein lacritin OS=Homo sapiens OX=9606 GN=LACRT PE=1 SV=1
Q9TSN6	39.96	18	2.89E+04	2 (2)	Carbamidomethylation	16275	Alpha-lactalbumin OS=Bubalus bubalis OX=89462 GN=LALBA PE=1 SV=2
P00711	39.96	18	2.89E+04	2 (2)	Carbamidomethylation	16247	Alpha-lactalbumin OS=Bos taurus OX=9913 GN=LALBA PE=1 SV=2
B1IXF6	73.07	24	6.79E+05	3 (3)		18695	DNA protection during starvation protein OS=Escherichia coli (strain ATCC 8739 / DSM 1576 / Crooks) OX=481805 GN=dps PE=3 SV=1
B7NNP4	73.07	24	6.79E+05	3 (3)		18695	DNA protection during starvation protein OS=Escherichia coli O7:K1 (strain IAI39 / ExPEC) OX=585057 GN=dps PE=3 SV=1
B7LC95	73.07	24	6.79E+05	3 (3)		18695	DNA protection during starvation protein OS=Escherichia coli (strain 55989 / EAEC) OX=585055 GN=dps PE=3 SV=1
C4ZXY4	73.07	24	6.79E+05	3 (3)		18695	DNA protection during starvation protein OS=Escherichia coli (strain K12 / MC4100 / BW2952) OX=595496 GN=dps PE=3 SV=1
Q6XZR0	73.07	24	6.79E+05	3 (3)		18695	DNA protection during starvation protein OS=Kluyvera cryocrescens OX=580 GN=dps PE=3 SV=1
B7LMB6	73.07	24	6.79E+05	3 (3)		18695	DNA protection during starvation protein OS=Escherichia fergusonii (strain ATCC 35469 / DSM 13698 / CDC 0568-73) OX=585054 GN=dps PE=3 SV=1
Q323Y1	73.07	24	6.79E+05	3 (3)		18695	DNA protection during starvation protein OS=Shigella boydii serotype 4 (strain Sb227) OX=300268 GN=dps PE=3 SV=3

Accession	-10lgP	Coverage (%)	Area	#Peptides (#unique)	PTM	Avg. Mass	Description
B7MQR6	73.07	24	6.79E+05	3 (3)		18695	DNA protection during starvation protein OS=Escherichia coli O81 (strain ED1a) OX=585397 GN=dps PE=3 SV=1
P0ABT2	73.07	24	6.79E+05	3 (3)		18695	DNA protection during starvation protein OS=Escherichia coli (strain K12) OX=83333 GN=dps PE=1 SV=2
P0ABT3	73.07	24	6.79E+05	3 (3)		18695	DNA protection during starvation protein OS=Escherichia coli O157:H7 OX=83334 GN=dps PE=3 SV=2
P0ABT4	73.07	24	6.79E+05	3 (3)		18695	DNA protection during starvation protein OS=Shigella flexneri OX=623 GN=dps PE=3 SV=2
Q6Y1R6	73.07	24	6.79E+05	3 (3)		18722	DNA protection during starvation protein OS=Proteus hauseri OX=183417 GN=dps PE=3 SV=1
Q3Z3X3	73.07	24	6.79E+05	3 (3)		18695	DNA protection during starvation protein OS=Shigella sonnei (strain Ss046) OX=300269 GN=dps PE=3 SV=3
B7MGS0	73.07	24	6.79E+05	3 (3)		18695	DNA protection during starvation protein OS=Escherichia coli O45:K1 (strain S88 / ExPEC) OX=585035 GN=dps PE=3 SV=1
Q8FJM0	73.07	24	6.79E+05	3 (3)		18753	DNA protection during starvation protein OS=Escherichia coli O6:H1 (strain CFT073 / ATCC 700928 / UPEC) OX=199310 GN=dps PE=3 SV=4
B1LMA4	73.07	24	6.79E+05	3 (3)		18695	DNA protection during starvation protein OS=Escherichia coli (strain SMS-3-5 / SECEC) OX=439855 GN=dps PE=3 SV=1
P02666	70.74	14	4.20E+05	4 (4)		25107	Beta-casein OS=Bos taurus OX=9913 GN=CSN2 PE=1 SV=2
P32119	66.84	20	8.66E+03	3 (2)		21892	Peroxiredoxin-2 OS=Homo sapiens OX=9606 GN=PRDX2 PE=1 SV=5
Q2PFZ3	66.84	20	8.66E+03	3 (2)		21892	Peroxiredoxin-2 OS=Macaca fascicularis OX=9541 GN=PRDX2 PE=2 SV=3
Q8K3U7	66.84	20	8.66E+03	3 (2)		21813	Peroxiredoxin-2 OS=Cricetulus griseus OX=10029 GN=PRDX2 PE=2 SV=3
Q5RC63	66.84	22	8.66E+03	3 (2)		19430	Peroxiredoxin-2 OS=Pongo abelii OX=9601 GN=PRDX2 PE=2 SV=3
Q9BYR0	66.45	10	2.19E+05	3 (2)	Carbamidomethylation	22535	Keratin-associated protein 4-7 OS=Homo sapiens OX=9606 GN=KRTAP4-7 PE=1 SV=2
Q9BQ66	66.45	10	2.19E+05	3 (2)	Carbamidomethylation	21407	Keratin-associated protein 4-12 OS=Homo sapiens OX=9606 GN=KRTAP4-12 PE=1 SV=1
Q9BYQ5	66.45	10	2.19E+05	3 (2)	Carbamidomethylation	21825	Keratin-associated protein 4-6 OS=Homo sapiens OX=9606 GN=KRTAP4-6 PE=2 SV=4
Q9BYR2	66.45	12	2.19E+05	3 (2)	Carbamidomethylation	19363	Keratin-associated protein 4-5 OS=Homo sapiens OX=9606 GN=KRTAP4-5 PE=1 SV=4
P35321	66.44	34	8.92E+04	3 (3)	Carbamidomethylation	9877	Cornifin-A OS=Homo sapiens OX=9606 GN=SPRR1A PE=1 SV=2
P35322	66.44	34	8.92E+04	3 (3)	Carbamidomethylation	9852	Cornifin OS=Macaca mulatta OX=9544 GN=SPRR1 PE=2 SV=1

Accession	-10lgP	Coverage (%)	Area	#Peptides (#unique)	PTM	Avg. Mass	Description
P22528	66.44	34	8.92E+04	3 (3)	Carbamidomethylation	9888	Cornifin-B OS=Homo sapiens OX=9606 GN=SPRR1B PE=1 SV=2
Q9BYE4	65.62	30	1.00E+05	2 (2)	Carbamidomethylation	8158	Small proline-rich protein 2G OS=Homo sapiens OX=9606 GN=SPRR2G PE=3 SV=1
P22531	65.62	31	1.00E+05	2 (2)	Carbamidomethylation	7855	Small proline-rich protein 2E OS=Homo sapiens OX=9606 GN=SPRR2E PE=2 SV=2
P22532	65.62	31	1.00E+05	2 (2)	Carbamidomethylation	7905	Small proline-rich protein 2D OS=Homo sapiens OX=9606 GN=SPRR2D PE=2 SV=2
P35326	65.62	31	1.00E+05	2 (2)	Carbamidomethylation	7965	Small proline-rich protein 2A OS=Homo sapiens OX=9606 GN=SPRR2A PE=1 SV=1
P35325	65.62	31	1.00E+05	2 (2)	Carbamidomethylation	7975	Small proline-rich protein 2B OS=Homo sapiens OX=9606 GN=SPRR2B PE=2 SV=1
P59666	64.73	19	2.00E+05	2 (2)	Carbamidomethylation	10245	Neutrophil defensin 3 OS=Homo sapiens OX=9606 GN=DEFA3 PE=1 SV=1
Q5G863	64.73	19	2.00E+05	2 (2)	Carbamidomethylation	10196	Neutrophil defensin 1 OS=Pan troglodytes OX=9598 GN=DEFA1 PE=3 SV=1
P59665	64.73	19	2.00E+05	2 (2)	Carbamidomethylation	10201	Neutrophil defensin 1 OS=Homo sapiens OX=9606 GN=DEFA1 PE=1 SV=1
P10599	62.95	23	2.25E+05	2 (2)		11737	Thioredoxin OS=Homo sapiens OX=9606 GN=TXN PE=1 SV=3
Q5R9M3	62.95	23	2.25E+05	2 (2)		11885	Thioredoxin OS=Pongo abelii OX=9601 GN=TXN PE=3 SV=3
P0DOY3	62.95	27	2.18E+05	2 (2)		11266	Immunoglobulin lambda constant 3 OS=Homo sapiens OX=9606 GN=IGLC3 PE=1 SV=1
P0DOY2	62.95	27	2.18E+05	2 (2)		11294	Immunoglobulin lambda constant 2 OS=Homo sapiens OX=9606 GN=IGLC2 PE=1 SV=1
O75223	60.33	17	8.47E+04	3 (3)		21008	Gamma-glutamylcyclotransferase OS=Homo sapiens OX=9606 GN=GGCT PE=1 SV=1
Q9BYT5	58.28	20	2.72E+05	3 (3)	Carbamidomethylation	12957	Keratin-associated protein 2-2 OS=Homo sapiens OX=9606 GN=KRTAP2-2 PE=2 SV=3
Q9BYR9	58.28	20	2.72E+05	3 (3)	Carbamidomethylation	13480	Keratin-associated protein 2-4 OS=Homo sapiens OX=9606 GN=KRTAP2-4 PE=1 SV=1
Q9BYU5	58.28	20	2.72E+05	3 (3)	Carbamidomethylation	13514	Keratin-associated protein 2-1 OS=Homo sapiens OX=9606 GN=KRTAP2-1 PE=2 SV=2
P0C7H8	58.28	20	2.72E+05	3 (3)	Carbamidomethylation	13480	Keratin-associated protein 2-3 OS=Homo sapiens OX=9606 GN=KRTAP2-3 PE=1 SV=2
P04792	57.48	20	1.31E+05	3 (3)		22783	Heat shock protein beta-1 OS=Homo sapiens OX=9606 GN=HSPB1 PE=1 SV=2
P84050	55.35	19	9.08E+04	2 (2)		11381	Histone H4 OS=Rhynchosciara americana OX=7186 GN=His4 PE=3 SV=2

Accession	-10lgP	Coverage (%)	Area	#Peptides (#unique)	PTM	Avg. Mass	Description
P62779	55.35	19	9.08E+04	2 (2)		11369	Histone H4 OS=Pycnopodia helianthoides OX=7614 PE=3 SV=2
Q28DR4	55.35	19	9.08E+04	2 (2)		11367	Histone H4 OS=Xenopus tropicalis OX=8364 GN=TGas006m08.1 PE=3 SV=1
P62797	55.35	19	9.08E+04	2 (2)		11367	Histone H4 OS=Oncorhynchus mykiss OX=8022 PE=1 SV=2
P62783	55.35	19	9.08E+04	2 (2)		11369	Histone H4 OS=Strongylocentrotus purpuratus OX=7668 PE=3 SV=2
P62778	55.35	19	9.08E+04	2 (2)		11369	Histone H4 OS=Pisaster ochraceus OX=7612 PE=3 SV=2
P62777	55.35	19	9.08E+04	2 (2)		11369	Histone H4 OS=Pisaster brevispinus OX=7611 PE=3 SV=2
P62802	55.35	19	9.08E+04	2 (2)		11367	Histone H4 OS=Sus scrofa OX=9823 PE=1 SV=2
P62798	55.35	19	9.08E+04	2 (2)		11367	Histone H4 OS=Xenopus borealis OX=8354 PE=3 SV=2
P62794	55.35	19	9.08E+04	2 (2)		11367	Histone H4 OS=Urechis caupo OX=6431 PE=3 SV=2
Q5RCS7	55.35	19	9.08E+04	2 (2)		11367	Histone H4 OS=Pongo abelii OX=9601 PE=3 SV=1
P62795	55.35	19	9.08E+04	2 (2)		11367	Histone H4 OS=Platynereis dumerilii OX=6359 PE=3 SV=2
P27996	55.35	19	9.08E+04	2 (2)		11385	Histone H4 OS=Solaster stimpsoni OX=7598 PE=3 SV=2
P62780	55.35	19	9.08E+04	2 (2)		11369	Histone H4 OS=Paracentrotus lividus OX=7656 PE=3 SV=2
Q27765	55.35	19	9.08E+04	2 (2)		11349	Histone H4 OS=Styela plicata OX=7726 PE=3 SV=3
P62796	55.35	19	9.08E+04	2 (2)		11367	Histone H4 OS=Oreochromis niloticus OX=8128 PE=3 SV=2
P83865	55.35	19	9.08E+04	2 (2)		11397	Histone H4 OS=Penaeus vannamei OX=6689 PE=1 SV=2
P62799	55.35	19	9.08E+04	2 (2)		11367	Histone H4 OS=Xenopus laevis OX=8355 PE=1 SV=2
Q6WV73	55.35	19	9.08E+04	2 (2)		11367	Histone H4 OS=Mytilus californianus OX=6549 PE=3 SV=3
Q43083	55.35	19	9.08E+04	2 (2)		11425	Histone H4 OS=Pyrenomonas salina OX=3034 PE=3 SV=3
P62781	55.35	19	9.08E+04	2 (2)		11369	Histone H4 OS=Psammechinus miliaris OX=7660 PE=1 SV=2

Accession	-10lgP	Coverage (%)	Area	#Peptides (#unique)	PTM	Avg. Mass	Description
P84045	55.35	19	9.08E+04	2 (2)		11381	Histone H4 OS=Tigriopus californicus OX=6832 GN=His4 PE=3 SV=2
P62804	55.35	19	9.08E+04	2 (2)		11367	Histone H4 OS=Rattus norvegicus OX=10116 GN=H4c2 PE=1 SV=2
P91890	55.35	19	9.08E+04	2 (2)		11337	Histone H4 OS=Trichogramma cacaeciae OX=1667566 PE=3 SV=3
P02663	55.32	10	3.86E+05	2 (2)		26019	Alpha-S2-casein OS=Bos taurus OX=9913 GN=CSN1S2 PE=1 SV=2
Q9GZZ8	50.89	15	6.96E+03	2 (2)	Oxidation (M)	14246	Extracellular glycoprotein lacritin OS=Homo sapiens OX=9606 GN=LACRT PE=1 SV=1
Q9TSN6	39.96	18	2.89E+04	2 (2)	Carbamidomethylation	16275	Alpha-lactalbumin OS=Bubalus bubalis OX=89462 GN=LALBA PE=1 SV=2
P00711	39.96	18	2.89E+04	2 (2)	Carbamidomethylation	16247	Alpha-lactalbumin OS=Bos taurus OX=9913 GN=LALBA PE=1 SV=2

(only matches with at least 2 unique peptides and above 10% coverage are listed)

Table S 14 - Protein matches obtained for Paestum Residue against Uniprot Honey Database

Accession	-10lgP	Coverage (%)	Area	#Peptides (#unique)	PTM	Avg. Mass	Description
O18330	144.42	23	7.99E+05	8 (8)	Oxidation (M)	48886	Major royal jelly protein 1 OS=Apis mellifera OX=7460 GN=MRJP1 PE=1 SV=1
O77061	138.49	13	7.23E+04	4 (3)	Oxidation (M)	51074	Major royal jelly protein 2 OS=Apis mellifera OX=7460 GN=MRJP2 PE=1 SV=1
A0A2A3E8T5	127.07	16	3.33E+05	5 (4)		45827	Actin OS=Apis cerana cerana OX=94128 GN=APICC_08334 PE=3 SV=1
A0A1V9XF46	127.07	17	3.33E+05	5 (4)		41839	Actin-5C-like OS=Tropilaelaps mercedesae OX=418985 GN=BIW11_10578 PE=3 SV=1
A0A2A3EM69	127.07	17	3.33E+05	5 (4)		41806	Actin-5C OS=Apis cerana cerana OX=94128 GN=APICC_05085 PE=3 SV=1
Q17060	126.26	18	3.93E+05	9 (6)	Oxidation (M)	61662	Major royal jelly protein 3 OS=Apis mellifera OX=7460 GN=MRJP3 PE=1 SV=1
A0A1V9XR76	83.55	5	1.39E+05	2 (2)		54441	Polyubiquitin-C isoform X1 OS=Tropilaelaps mercedesae OX=418985 GN=BIW11_08057 PE=4 SV=1
A0A2A3EG18	74.11	12	6.17E+04	5 (5)	Deamidation (NQ); Oxidation (M)	71086	Heat shock OS=Apis cerana cerana OX=94128 GN=APICC_04534 PE=3 SV=1
A0A2H3EAA9	69.38	13	7.17E+04	3 (2)	Deamidation (NQ); Oxidation (M)	41855	Actin 2 OS=Armillaria gallica OX=47427 GN=ARMGADRAFT_9722 PE=3 SV=1
A0A1V9XJI7	48.38	11	3.30E+05	3 (3)		60119	SWI/SNF-related matrix-associated actin-dependent regulator of chromatin subfamily D member 1-like OS=Tropilaelaps mercedesae OX=418985 GN=BIW11_09604 PE=4 SV=1
A0A2A3ECX5	47.43	11	1.97E+05	2 (2)	Carbamidomethylation	29815	Trypsin-2 OS=Apis cerana cerana OX=94128 GN=APICC_08270 PE=4 SV=1
A0A2A3EEC5	44.20	6	1.53E+05	5 (5)	Deamidation (NQ); Oxidation (M)	65227	Myelin expression factor OS=Apis cerana cerana OX=94128 GN=APICC_05731 PE=4 SV=1
A0A1V9XCK9	41.78	34	1.21E+04	4 (4)	Deamidation (NQ)	9408	UPF0480 protein C15orf24-like OS=Tropilaelaps mercedesae OX=418985 GN=BIW11_11081 PE=4 SV=1
A0A2A3E5C5	41.49	5	1.43E+05	3 (3)		65889	Broad-complex core protein OS=Apis cerana cerana OX=94128 GN=APICC_08530 PE=4 SV=1
A0A1V9XNM0	38.71	29	2.79E+05	3 (3)	Deamidation (NQ)	17125	Uncharacterized protein OS=Tropilaelaps mercedesae OX=418985 GN=BIW11_00866 PE=4 SV=1
A0A1V9XXZ1	37.88	8	9.08E+04	2 (2)		28105	Histone H4 OS=Tropilaelaps mercedesae OX=418985 GN=BIW11_06453 PE=3 SV=1
A0A1V9XAM9	37.88	19	9.08E+04	2 (2)		11367	Histone H4 OS=Tropilaelaps mercedesae OX=418985 GN=BIW11_01686 PE=3 SV=1
A0A2A3EQG4	37.88	19	9.08E+04	2 (2)		11381	Histone H4 OS=Apis cerana cerana OX=94128 GN=APICC_03967 PE=3 SV=1
A0A2H3E8S8	36.42	9	2.65E+05	2 (2)		30302	Uncharacterized protein (Fragment) OS=Armillaria gallica OX=47427 GN=ARMGADRAFT_899934 PE=4 SV=1

Accession	-10lgP	Coverage (%)	Area	#Peptides (#unique)	PTM	Avg. Mass	Description
A0A0F4LV26	36.21	9	6.17E+06	2 (2)	Carbamidomethylation; Deamidation (NQ)	23841	Uncharacterized protein OS=Lactobacillus apis OX=303541 GN=JF72_02070 PE=3 SV=1
A0A074V7Z4	34.56	5	3.78E+05	2 (1)	Deamidation (NQ)	38024	Holliday junction ATP-dependent DNA helicase RuvB OS=Snodgrassella alvi SCGC AB-598-J21 OX=1385367 GN=ruvB PE=3 SV=1
A0A0M9DG86	32.99	5	5.22E+03	2 (1)	Deamidation (NQ)	49265	Uncharacterized protein (Fragment) OS=Lactobacillus kunkeei OX=148814 GN=RZ72_06780 PE=4 SV=1
A0A0R1SIN9	32.61	8	7.61E+04	3 (3)	Oxidation (M)	48309	Glutathione reductase OS=Lactobacillus versmoldensis DSM 14857 = KCTC 3814 OX=1423815 GN=FC27_GL000144 PE=3 SV=1
A0A0F4LUU7	28.55	6	1.01E+05	2 (2)	Deamidation (NQ)	39690	Uncharacterized protein OS=Lactobacillus apis OX=303541 GN=JF72_02620 PE=4 SV=1
A0A1L8WSP4	27.62	5	4.45E+04	2 (2)	Deamidation (NQ)	47841	PTS system Fru family IIC component OS=Enterococcus raffinosus OX=71452 GN=RV13_GL002542 PE=4 SV=1
R2RKK3	27.62	5	4.45E+04	2 (2)	Deamidation (NQ)	49905	PTS system Fru family IIC component OS=Enterococcus raffinosus ATCC 49464 OX=1158602 GN=UAK_01026 PE=4 SV=1
A0A2A3EIS3	27.46	6	1.64E+06	2 (2)		56664	Inosine-5'-monophosphate dehydrogenase OS=Apis cerana cerana OX=94128 GN=APICC_05459 PE=3 SV=1
A0A080KLQ4	26.85	8	4.52E+05	2 (1)	Deamidation (NQ)	20781	Ribosome-recycling factor OS=Gilliamella apicola SCGC AB-598-I20 OX=1445514 GN=frr PE=3 SV=1
T0L9D2	26.58	9	3.64E+05	2 (2)		27082	Uncharacterized protein OS=Nosema apis BRL 01 OX=1037528 GN=NAPIS_ORF01322 PE=4 SV=1
A0A4R0SKY4	26.54	8	1.89E+06	1 (1)		13953	Uncharacterized protein OS=Bifidobacterium longum subsp. longum OX=1679 GN=MCC10007_2122 PE=4 SV=1
A0A0F4LUV6	26.46	5	7.34E+05	2 (2)	Deamidation (NQ)	98333	Alanine--tRNA ligase OS=Lactobacillus apis OX=303541 GN=alaS PE=3 SV=1
A0A074VCI0	26.33	9	1.34E+04	2 (1)		16415	Uncharacterized protein OS=Snodgrassella alvi SCGC AB-598-J21 OX=1385367 GN=SASC598J21_020630 PE=4 SV=1
X2HCR0	26.33	9	1.34E+04	2 (1)		16415	Transamidase GatB domain protein OS=Snodgrassella alvi wkB2 OX=1196094 GN=SALWKB2_1475 PE=4 SV=1
A0A4R0S5G1	25.83	5	1.41E+04	2 (1)		35271	Glycerate kinase OS=Bifidobacterium longum subsp. longum OX=1679 GN=MCC10002_1002 PE=3 SV=1
A0A1V9XRD7	25.22	5	5.86E+04	2 (2)	Deamidation (NQ)	122596	Pumilio2 isoform X4-like OS=Tropilaelaps mercedesae OX=418985 GN=BIW11_08020 PE=4 SV=1
A0A347SQJ1	25.09	15	2.07E+05	1 (1)	Deamidation (NQ); Oxidation (M)	8622	Uncharacterized protein OS=Lactobacillus bombi OX=1303590 GN=DS830_01945 PE=4 SV=1
A0A1L8X669	24.50	6	2.67E+04	1 (1)		15771	PTS EIIA type-2 domain-containing protein OS=Enterococcus raffinosus OX=71452 GN=RV13_GL002159 PE=4 SV=1
R2REN2	24.50	6	2.67E+04	1 (1)		15771	PTS EIIA type-2 domain-containing protein OS=Enterococcus raffinosus ATCC 49464 OX=1158602 GN=UAK_00371 PE=4 SV=1
A0A0S2MI51	24.24	5	3.53E+04	1 (1)	Oxidation (M)	33824	Uncharacterized protein OS=Bifidobacterium longum subsp. longum OX=1679 GN=BBL306_0816 PE=4 SV=1

Accession	-10lgP	Coverage (%)	Area	#Peptides (#unique)	PTM	Avg. Mass	Description
A0A2A3EQT8	23.97	8	2.31E+04	2 (2)		38908	Uncharacterized protein OS=Apis cerana cerana OX=94128 GN=APICC_10078 PE=4 SV=1
C4VAY5	23.24	5	2.41E+04	1 (1)		29591	Uncharacterized protein OS=Nosema ceranae (strain BRL01) OX=578460 GN=NCER_101890 PE=4 SV=1
A0A2H3CX12	22.54	6	3.00E+05	1 (1)		15998	Uncharacterized protein OS=Armillaria gallica OX=47427 GN=ARMGADRAFT_535066 PE=4 SV=1
A0A2H3DBF6	22.54	6	3.00E+05	1 (1)		17652	Uncharacterized protein OS=Armillaria gallica OX=47427 GN=ARMGADRAFT_1086583 PE=4 SV=1
D5H196	21.40	6	1.35E+05	1 (1)		21189	DJ-1 family protease OS=Lactobacillus crispatus (strain ST1) OX=748671 GN=thiJ PE=4 SV=1
A0A0F4L9Y1	20.41	11	6.37E+05	1 (1)		11278	Cytosolic protein YlxR OS=Lactobacillus kullabergensis OX=1218493 GN=ylxR PE=4 SV=1
A0A0F4LK07	20.41	11	6.37E+05	1 (1)		11332	Cytosolic protein YlxR OS=Lactobacillus kimbladii OX=1218506 GN=ylxR PE=4 SV=1
A0A0F4LGN0	20.41	12	6.37E+05	1 (1)		10434	Cytosolic protein YlxR OS=Lactobacillus melliventris OX=1218507 GN=ylxR PE=4 SV=1
T0M9W8	20.25	5	2.17E+04	1 (1)	Deamidation (NQ)	59294	Uncharacterized protein OS=Nosema apis BRL 01 OX=1037528 GN=NAPIS_ORF02250 PE=4 SV=1
A0A1V9XS31	20.20	7	3.31E+04	1 (1)	Carbamidomethylation; Deamidation (NQ)	37087	Mediator of RNA polymerase II transcription subunit 27-like OS=Tropilaelaps mercedesae OX=418985 GN=BIW11_07849 PE=3 SV=1

(only matches with at least 5% coverage and 1 unique peptide are listed)

Table S 15 - Protein matches obtained for Honeycomb Greece Fresh against Uniprot All Proteins Database

Accession	-10lgP	Coverage (%)	Area	#Peptides (#unique)	PTM	Avg. Mass	Description
P35527	323.41	95	3.23E+08	180 (175)	Carbamidomethylation; Oxidation (M); Deamidation (NQ)	62064	Keratin type I cytoskeletal 9 OS=Homo sapiens GN=KRT9 PE=1 SV=3
P04264	310.44	88	1.76E+08	175 (105)	Carbamidomethylation; Oxidation (M); Deamidation (NQ)	66039	Keratin type II cytoskeletal 1 OS=Homo sapiens GN=KRT1 PE=1 SV=6
P35908	290.02	85	2.68E+07	85 (47)	Carbamidomethylation; Oxidation (M); Deamidation (NQ)	65433	Keratin type II cytoskeletal 2 epidermal OS=Homo sapiens GN=KRT2 PE=1 SV=2
P13645	286.54	78	1.52E+08	101 (52)	Carbamidomethylation; Oxidation (M); Deamidation (NQ)	58827	Keratin type I cytoskeletal 10 OS=Homo sapiens GN=KRT10 PE=1 SV=6
P00761	279.37	94	3.52E+08	118 (60)	Carbamidomethylation; Oxidation (M); Deamidation (NQ)	24409	Trypsin OS=Sus scrofa PE=1 SV=1
Q86YZ3	250.68	24	6.50E+06	104 (102)	Deamidation (NQ)	282389	Hornerin OS=Homo sapiens GN=HRNR PE=1 SV=2
P04259	247.63	71	1.46E+05	67 (2)	Carbamidomethylation; Oxidation (M); Deamidation (NQ)	60067	Keratin type II cytoskeletal 6B OS=Homo sapiens GN=KRT6B PE=1 SV=5
P02533	235.09	67	5.65E+06	49 (8)	Carbamidomethylation; Oxidation (M); Deamidation (NQ)	51562	Keratin type I cytoskeletal 14 OS=Homo sapiens GN=KRT14 PE=1 SV=4
P13647	228.71	58	4.58E+06	61 (10)	Carbamidomethylation; Oxidation (M); Deamidation (NQ)	62378	Keratin type II cytoskeletal 5 OS=Homo sapiens GN=KRT5 PE=1 SV=3
Q6EIZ0	216.75	38	1.43E+06	50 (2)	Carbamidomethylation; Oxidation (M); Deamidation (NQ)	57711	Keratin type I cytoskeletal 10 OS=Canis lupus familiaris GN=KRT10 PE=2 SV=1
P08779	216.61	66	5.17E+06	46 (18)	Carbamidomethylation; Oxidation (M); Deamidation (NQ)	51268	Keratin type I cytoskeletal 16 OS=Homo sapiens GN=KRT16 PE=1 SV=4
P15924	207.75	15	1.94E+06	43 (4)	Carbamidomethylation; Oxidation (M)	331774	Desmoplakin OS=Homo sapiens GN=DSP PE=1 SV=3
O18330	206.32	64	2.05E+07	34 (28)	Carbamidomethylation; Oxidation (M); Deamidation (NQ)	48886	Major royal jelly protein 1 OS=Apis mellifera OX=7460 GN=MRJP1 PE=1 SV=1
Q04695	203.55	53	1.44E+06	34 (4)	Carbamidomethylation; Oxidation (M); Deamidation (NQ)	48106	Keratin type I cytoskeletal 17 OS=Homo sapiens GN=KRT17 PE=1 SV=2
Q8NHM4	200.64	38	2.10E+06	36 (2)	Carbamidomethylation; Deamidation (NQ)	26537	Putative trypsin-6 OS=Homo sapiens GN=PRSS3P2 PE=5 SV=2
P23490	197.62	54	7.44E+05	22 (22)	Carbamidomethylation	25761	Loricrin OS=Homo sapiens GN=LOR PE=1 SV=2
Q02SZ7	195.45	38	1.36E+07	21 (21)	Carbamidomethylation; Oxidation (M); Deamidation (NQ)	48178	Lysyl endopeptidase OS=Pseudomonas aeruginosa (strain UCBPP-PA14) OX=208963 GN=prpL PE=1 SV=1
Q9HWK6	195.45	38	1.36E+07	21 (21)	Carbamidomethylation; Oxidation (M); Deamidation (NQ)	48213	Lysyl endopeptidase OS=Pseudomonas aeruginosa (strain ATCC 15692 / DSM 22644 / CIP 104116 / JCM 14847 / LMG 12228 / 1C / PRS 101 / PAO1) OX=208964 GN=prpL PE=1 SV=1
Q17060	188.97	46	6.41E+06	27 (23)	Carbamidomethylation; Oxidation (M); Deamidation (NQ)	61662	Major royal jelly protein 3 OS=Apis mellifera OX=7460 GN=MRJP3 PE=1 SV=1
Q6EIZ1	169.01	28	5.91E+05	30 (2)	Carbamidomethylation; Oxidation (M)	64575	Keratin type II cytoskeletal 2 epidermal OS=Canis lupus familiaris GN=KRT2 PE=2 SV=1
Q6IG02	168.2	19	5.15E+04	25 (3)	Carbamidomethylation; Oxidation (M)	69127	Keratin type II cytoskeletal 2 epidermal OS=Rattus norvegicus GN=Krt2 PE=3 SV=1
P02768	160.69	27	1.61E+06	17 (17)	Carbamidomethylation	69367	Serum albumin OS=Homo sapiens GN=ALB PE=1 SV=2

Accession	-10lgP	Coverage (%)	Area	#Peptides (#unique)	PTM	Avg. Mass	Description
Q02413	159.04	14	4.12E+05	13 (11)	Carbamidomethylation; Oxidation (M)	113748	Desmoglein-1 OS=Homo sapiens GN=DSG1 PE=1 SV=2
O77061	158.36	35	4.39E+06	15 (11)	Carbamidomethylation; Oxidation (M); Deamidation (NQ)	51074	Major royal jelly protein 2 OS=Apis mellifera OX=7460 GN=MRJP2 PE=1 SV=1
O97432	154.34	27	1.76E+06	16 (11)	Oxidation (M)	70236	Major royal jelly protein 5 OS=Apis mellifera OX=7460 GN=MRJP5 PE=2 SV=1
P00760	151.2	40	4.35E+06	19 (6)	Carbamidomethylation; Deamidation (NQ)	25785	Cationic trypsin OS=Bos taurus PE=1 SV=3
Q7Z794	150.86	21	3.11E+04	20 (3)	Carbamidomethylation; Oxidation (M)	61901	Keratin type II cytoskeletal 1b OS=Homo sapiens GN=KRT77 PE=1 SV=3
P02788	149.9	18	1.65E+05	10 (8)	Carbamidomethylation	78182	Lactotransferrin OS=Homo sapiens GN=LTF PE=1 SV=6
Q6IG01	148.65	16	7.79E+05	20 (2)	Oxidation (M)	57255	Keratin type II cytoskeletal 1b OS=Rattus norvegicus GN=Krt77 PE=3 SV=1
Q01546	146.96	21	2.05E+04	26 (3)	Carbamidomethylation; Oxidation (M); Deamidation (NQ)	65841	Keratin type II cytoskeletal 2 oral OS=Homo sapiens GN=KRT76 PE=1 SV=2
Q8N1N4	146.72	26	1.24E+05	21 (9)	Carbamidomethylation	56866	Keratin type II cytoskeletal 78 OS=Homo sapiens GN=KRT78 PE=1 SV=2
Q5D862	142.8	8	6.25E+05	20 (18)	Carbamidomethylation; Oxidation (M); Deamidation (NQ)	248072	Filaggrin-2 OS=Homo sapiens GN=FLG2 PE=1 SV=1
Q02257	142.23	19	5.01E+05	14 (14)	Carbamidomethylation; Oxidation (M)	81801	Junction plakoglobin OS=Mus musculus GN=Jup PE=1 SV=3
Q6P0K8	142.23	19	5.01E+05	14 (14)	Carbamidomethylation; Oxidation (M)	81801	Junction plakoglobin OS=Rattus norvegicus GN=Jup PE=1 SV=1
Q8SPJ1	142.23	19	5.01E+05	14 (14)	Carbamidomethylation; Oxidation (M)	81821	Junction plakoglobin OS=Bos taurus GN=JUP PE=2 SV=1
P14923	142.23	19	5.01E+05	14 (14)	Carbamidomethylation; Oxidation (M)	81745	Junction plakoglobin OS=Homo sapiens GN=JUP PE=1 SV=3
Q8WNW3	142.23	19	5.01E+05	14 (14)	Carbamidomethylation; Oxidation (M)	81850	Junction plakoglobin OS=Sus scrofa GN=Jup PE=2 SV=1
P81605	141.79	77	1.59E+07	19 (18)	Carbamidomethylation	11284	Dermcidin OS=Homo sapiens GN=DCD PE=1 SV=2
P0AEX9	141.17	39	4.97E+06	17 (17)	Oxidation (M)	43388	Maltose/maltodextrin-binding periplasmic protein OS=Escherichia coli (strain K12) OX=83333 GN=malE PE=1 SV=1
P0AEY0	141.17	39	4.97E+06	17 (17)	Oxidation (M)	43388	Maltose/maltodextrin-binding periplasmic protein OS=Escherichia coli O157:H7 OX=83334 GN=malE PE=1 SV=1
Q17058	139.42	28	1.11E+06	17 (17)	Carbamidomethylation; Oxidation (M); Deamidation (NQ)	65565	Alpha-glucosidase OS=Apis mellifera PE=1 SV=1
Q6UWP8	138.28	19	3.54E+05	9 (9)		60541	Suprabasin OS=Homo sapiens GN=SBSN PE=1 SV=2
P04280	134.85	52	4.25E+05	13 (6)	Deamidation (NQ)	38546	Basic salivary proline-rich protein 1 OS=Homo sapiens GN=PRB1 PE=1 SV=2
P01040	134.61	77	2.39E+06	8 (8)		11006	Cystatin-A OS=Homo sapiens GN=CSTA PE=1 SV=1
P12273	134.13	60	1.23E+06	7 (7)	Carbamidomethylation	16572	Prolactin-inducible protein OS=Homo sapiens GN=PIP PE=1 SV=1
P61628	129.75	61	1.93E+06	12 (11)	Carbamidomethylation; Oxidation (M); Deamidation (NQ)	16537	Lysozyme C OS=Pan troglodytes GN=LYZ PE=2 SV=1
P61626	129.75	61	1.93E+06	12 (11)	Carbamidomethylation; Oxidation (M); Deamidation (NQ)	16537	Lysozyme C OS=Homo sapiens GN=LYZ PE=1 SV=1

Accession	-10lgP	Coverage (%)	Area	#Peptides (#unique)	PTM	Avg. Mass	Description
P79179	129.75	61	1.93E+06	12 (11)	Carbamidomethylation; Oxidation (M); Deamidation (NQ)	16567	Lysozyme C OS=Gorilla gorilla gorilla GN=LYZ PE=2 SV=1
P61627	129.75	61	1.93E+06	12 (11)	Carbamidomethylation; Oxidation (M); Deamidation (NQ)	16537	Lysozyme C OS=Pan paniscus GN=LYZ PE=3 SV=1
Q04118	127.64	32	3.59E+06	12 (10)		30980	Basic salivary proline-rich protein 3 OS=Homo sapiens GN=PRB3 PE=1 SV=2
P00763	122.46	34	5.31E+05	19 (2)	Carbamidomethylation; Deamidation (NQ)	26228	Anionic trypsin-2 OS=Rattus norvegicus GN=Prss2 PE=1 SV=2
Q5T749	119.16	18	6.87E+05	10 (10)	Carbamidomethylation; Deamidation (NQ)	64136	Keratinocyte proline-rich protein OS=Homo sapiens GN=KPRP PE=1 SV=1
P04406	116.78	19	3.89E+05	6 (6)	Carbamidomethylation; Deamidation (NQ)	36053	Glyceraldehyde-3-phosphate dehydrogenase OS=Homo sapiens GN=GAPDH PE=1 SV=3
Q5RAB4	116.78	19	3.89E+05	6 (6)	Carbamidomethylation; Deamidation (NQ)	35993	Glyceraldehyde-3-phosphate dehydrogenase OS=Pongo abelii GN=GAPDH PE=2 SV=3
P05109	109.09	61	4.20E+05	6 (5)	Carbamidomethylation	10835	Protein S100-A8 OS=Homo sapiens GN=S100A8 PE=1 SV=1
Q96P63	108.42	16	7.54E+04	5 (5)		46276	Serpin B12 OS=Homo sapiens GN=SERPINB12 PE=1 SV=1
Q17061	107.29	14	2.19E+05	6 (5)	Oxidation (M)	52916	Major royal jelly protein 4 OS=Apis mellifera OX=7460 GN=MRJP4 PE=1 SV=1
Q96DA0	104.93	23	5.64E+05	4 (4)		22739	Zymogen granule protein 16 homolog B OS=Homo sapiens GN=ZG16B PE=1 SV=3
Q9NZT1	103.65	23	6.90E+05	4 (4)	Deamidation (NQ)	15893	Calmodulin-like protein 5 OS=Homo sapiens GN=CALML5 PE=1 SV=2
Q7YR44	100.44	16	4.78E+05	6 (6)	Carbamidomethylation	51611	Corneodesmosin OS=Pan troglodytes GN=CDSN PE=2 SV=1
P10163	94.66	21	1.38E+06	7 (5)		31326	Basic salivary proline-rich protein 4 OS=Homo sapiens GN=PRB4 PE=1 SV=4
P02810	85.7	72	7.89E+05	8 (8)		17016	Salivary acidic proline-rich phosphoprotein 1/2 OS=Homo sapiens GN=PRH1 PE=1 SV=2
P31151	80.71	25	1.48E+05	3 (3)	Carbamidomethylation; Deamidation (NQ)	11471	Protein S100-A7 OS=Homo sapiens GN=S100A7 PE=1 SV=4
Q9GZZ8	80.58	24	9.48E+04	3 (3)	Oxidation (M)	14246	Extracellular glycoprotein lacritin OS=Homo sapiens GN=LACRT PE=1 SV=1
P31944	80.17	19	1.87E+05	5 (5)	Carbamidomethylation	27680	Caspase-14 OS=Homo sapiens GN=CASP14 PE=1 SV=2
P25311	78.91	18	3.93E+05	4 (4)		34259	Zinc-alpha-2-glycoprotein OS=Homo sapiens GN=AZGP1 PE=1 SV=2
P07521	77.56	64	1.97E+05	4 (4)	Carbamidomethylation	9719	Feather keratin B-4 OS=Columba livia PE=1 SV=1
Q5G863	73.64	19	1.12E+05	2 (2)	Carbamidomethylation	10196	Neutrophil defensin 1 OS=Pan troglodytes GN=DEFA1 PE=3 SV=1
P59666	73.64	19	1.12E+05	2 (2)	Carbamidomethylation	10245	Neutrophil defensin 3 OS=Homo sapiens GN=DEFA3 PE=1 SV=1
P59665	73.64	19	1.12E+05	2 (2)	Carbamidomethylation	10201	Neutrophil defensin 1 OS=Homo sapiens GN=DEFA1 PE=1 SV=1
P01876	72.75	16	1.21E+05	5 (5)		37655	Immunoglobulin heavy constant alpha 1 OS=Homo sapiens GN=IGHA1 PE=1 SV=2
Q01469	72.11	27	1.15E+05	5 (5)	Carbamidomethylation	15164	Fatty acid-binding protein epidermal OS=Homo sapiens GN=FABP5 PE=1 SV=3

Accession	-10lgP	Coverage (%)	Area	#Peptides (#unique)	PTM	Avg. Mass	Description
Q9BYE4	69.83	30	2.72E+05	2 (2)	Carbamidomethylation	8158	Small proline-rich protein 2G OS=Homo sapiens GN=SPRR2G PE=3 SV=1
P22532	69.83	31	2.72E+05	2 (2)	Carbamidomethylation	7905	Small proline-rich protein 2D OS=Homo sapiens GN=SPRR2D PE=2 SV=2
P35325	69.83	31	2.72E+05	2 (2)	Carbamidomethylation	7975	Small proline-rich protein 2B OS=Homo sapiens GN=SPRR2B PE=2 SV=1
P35326	69.83	31	2.72E+05	2 (2)	Carbamidomethylation	7965	Small proline-rich protein 2A OS=Homo sapiens GN=SPRR2A PE=1 SV=1
P22531	69.83	31	2.72E+05	2 (2)	Carbamidomethylation	7855	Small proline-rich protein 2E OS=Homo sapiens GN=SPRR2E PE=2 SV=2
P02537	68.78	16	1.52E+06	9 (2)	Carbamidomethylation	51889	Keratin-3 type I cytoskeletal 51 kDa OS=Xenopus laevis PE=2 SV=1
Q5T750	67.26	14	2.21E+05	3 (3)	Carbamidomethylation	26238	Skin-specific protein 32 OS=Homo sapiens GN=XP32 PE=1 SV=1
P02791	65.67	11	3.07E+04	2 (2)		19978	Ferritin light chain OS=Equus caballus GN=FTL PE=1 SV=4
P67976	64.2	16	1.16E+05	2 (2)	Deamidation (NQ)	19921	Beta-lactoglobulin-1/B OS=Ovis aries PE=1 SV=1
P02756	64.2	16	1.16E+05	2 (2)	Deamidation (NQ)	19976	Beta-lactoglobulin OS=Capra hircus OX=9925 GN=LGB PE=1 SV=2
P67975	64.2	17	1.16E+05	2 (2)	Deamidation (NQ)	18151	Beta-lactoglobulin OS=Ovis aries musimon GN=LGB PE=1 SV=1
P31025	62.66	12	4.26E+05	2 (2)		19250	Lipocalin-1 OS=Homo sapiens GN=LCN1 PE=1 SV=1
Q5VSP4	62.66	14	4.26E+05	2 (2)		17918	Putative lipocalin 1-like protein 1 OS=Homo sapiens GN=LCN1P1 PE=5 SV=1
Q63532	51.46	11	1.61E+05	2 (2)	Carbamidomethylation	16734	Cornifin-A OS=Rattus norvegicus GN=Sprr1a PE=2 SV=1
P35323	51.46	16	1.61E+05	2 (2)	Carbamidomethylation	10724	Cornifin OS=Sus scrofa GN=SPRP PE=2 SV=1
P35321	51.46	18	1.61E+05	2 (2)	Carbamidomethylation	9877	Cornifin-A OS=Homo sapiens GN=SPRR1A PE=1 SV=2
P22528	51.46	18	1.61E+05	2 (2)	Carbamidomethylation	9888	Cornifin-B OS=Homo sapiens GN=SPRR1B PE=1 SV=2
P35322	51.46	18	1.61E+05	2 (2)	Carbamidomethylation	9852	Cornifin OS=Macaca mulatta GN=SPRR1 PE=2 SV=1
Q5T751	44.9	31	1.66E+04	3 (3)	Carbamidomethylation	11543	Late cornified envelope protein 1C OS=Homo sapiens GN=LCE1C PE=1 SV=1
P06702	42.09	18	1.26E+05	2 (2)		13242	Protein S100-A9 OS=Homo sapiens GN=S100A9 PE=1 SV=1

(only matches with at least 2 unique peptides and above 10% coverage are listed)

Table S 16 – Protein matches obtained for Honeycomb Greece Fresh against Uniprot Honey Database

Accession	-10lgP	Coverage (%)	Area	#Peptides (#unique)	PTM	Avg. Mass	Description
O18330	247.39	65	2.05E+07	36 (30)	Carbamidomethylation; Deamidation (NQ); Oxidation (M)	48886	Major royal jelly protein 1 OS=Apis mellifera OX=7460 GN=MRJP1 PE=1 SV=1
Q17060	221.91	45	4.37E+06	25 (15)	Carbamidomethylation; Deamidation (NQ); Oxidation (M)	61662	Major royal jelly protein 3 OS=Apis mellifera OX=7460 GN=MRJP3 PE=1 SV=1
O77061	183.16	35	4.04E+06	14 (7)	Carbamidomethylation; Deamidation (NQ); Oxidation (M)	51074	Major royal jelly protein 2 OS=Apis mellifera OX=7460 GN=MRJP2 PE=1 SV=1
O97432	175.01	25	1.72E+06	15 (11)	Oxidation (M)	70236	Major royal jelly protein 5 OS=Apis mellifera OX=7460 GN=MRJP5 PE=2 SV=1
A0A2A3EHI0	145.81	32	2.91E+03	11 (1)	Carbamidomethylation; Deamidation (NQ); Oxidation (M)	34111	Bee-milk protein OS=Apis cerana cerana OX=94128 GN=APICC_10183 PE=3 SV=1
A0A2A3E8C9	135.51	13	6.01E+05	9 (9)	Carbamidomethylation; Oxidation (M)	66292	Alpha-glucosidase OS=Apis cerana cerana OX=94128 GN=APICC_04097 PE=4 SV=1
B6V6A2	124.67	15	1.78E+05	4 (4)		33530	Silk fibroin 3 OS=Apis mellifera OX=7460 GN=Fibroin3 PE=2 SV=1
Q17061	117.12	14	2.19E+05	6 (4)	Oxidation (M)	52916	Major royal jelly protein 4 OS=Apis mellifera OX=7460 GN=MRJP4 PE=1 SV=1
B6V6A3	81.63	13	1.10E+05	4 (4)		33931	Silk fibroin 4 OS=Apis mellifera OX=7460 GN=Fibroin4 PE=2 SV=1
A0A1V9XCK9	36.54	29	4.26E+05	3 (3)		9408	UPF0480 protein C15orf24-like OS=Tropilaelaps mercedesae OX=418985 GN=BIW11_11081 PE=4 SV=1
A0A0M9D6H7	32.9	8	8.81E+04	2 (1)	Deamidation (NQ)	33079	GW domain-containing protein OS=Lactobacillus kunkeei OX=148814 GN=RZ55_02400 PE=4 SV=1
A0A6L4UQH2	32.88	5	1.32E+05	1 (1)	Carbamidomethylation	31693	ABC transporter OS=Bifidobacterium longum OX=216816 GN=GBA95_10565 PE=4 SV=1
A0A2S8A7Q1	28.51	6	6.97E+04	2 (2)	Deamidation (NQ)	50207	Transporter OS=Apibacter adventoris OX=1679466 GN=C4S77_11995 PE=3 SV=1
A0A4R0SKY4	28.5	8	9.40E+05	1 (1)		13953	Uncharacterized protein OS=Bifidobacterium longum subsp. longum OX=1679 GN=MCC10007_2122 PE=4 SV=1
A0A1S2VX47	28.5	9	9.40E+05	1 (1)		12737	Uncharacterized protein OS=Bifidobacterium longum subsp. infantis OX=1682 GN=BFS25_05850 PE=4 SV=1
V6XYG9	28.5	9	9.40E+05	1 (1)		12738	Uncharacterized protein OS=Bifidobacterium longum E18 OX=1322347 GN=BLONG_1920 PE=4 SV=1
A0A4R0USA9	28.5	9	9.40E+05	1 (1)		12738	Uncharacterized protein OS=Bifidobacterium longum subsp. longum OX=1679 GN=MCC10102_1887 PE=4 SV=1
A0A0R1SDB6	26.06	5	1.55E+05	1 (1)		22612	Flavoprotein OS=Lactobacillus versmoldensis DSM 14857 = KCTC 3814 OX=1423815 GN=FC27_GL000524 PE=4 SV=1

Accession	-10lgP	Coverage (%)	Area	#Peptides (#unique)	PTM	Avg. Mass	Description
A0A2A3EIH7	24.7	5	9.15E+04	2 (2)		62182	Protein alan shepard OS=Apis cerana cerana OX=94128 GN=APICC_07690 PE=4 SV=1
A0A083X698	22.71	12	1.23E+04	1 (1)	Deamidation (NQ); Oxidation (M)	13299	Glycosyl hydrolase family 3 OS=Bifidobacterium longum subsp. longum 17-1B OX=1350473 GN=BL171B_08425 PE=3 SV=1
A0A0R1RJ46	21.05	5	1.50E+04	1 (1)	Deamidation (NQ)	23772	50S ribosomal protein L3 OS=Lactobacillus rossiae DSM 15814 OX=1114972 GN=rplC PE=3 SV=1
A0A0F4LV26	20.93	9	3.74E+05	1 (1)	Carbamidomethylation; Deamidation (NQ)	23841	Uncharacterized protein OS=Lactobacillus apis OX=303541 GN=JF72_02070 PE=3 SV=1

(only matches with at least 5% coverage and 1 unique peptide are listed)

Table S 17 – Protein matches obtained for Honeycomb Greece Aged against Uniprot All Database

Accession	-10lgP	Coverage (%)	Area	#Peptides (#unique)	PTM	Avg. Mass	Description
P35527	323.68	94	4.22E+08	178 (164)	Carbamidomethylation; Oxidation (M); Deamidation (NQ)	62064	Keratin type I cytoskeletal 9 OS=Homo sapiens GN=KRT9 PE=1 SV=3
P04264	307.22	85	2.63E+08	199 (114)	Carbamidomethylation; Oxidation (M); Deamidation (NQ)	66039	Keratin type II cytoskeletal 1 OS=Homo sapiens GN=KRT1 PE=1 SV=6
P13645	292.64	86	1.57E+08	105 (49)	Carbamidomethylation; Oxidation (M); Deamidation (NQ)	58827	Keratin type I cytoskeletal 10 OS=Homo sapiens GN=KRT10 PE=1 SV=6
P35908	281.37	87	4.68E+07	101 (51)	Carbamidomethylation; Oxidation (M); Deamidation (NQ)	65433	Keratin type II cytoskeletal 2 epidermal OS=Homo sapiens GN=KRT2 PE=1 SV=2
P00761	275.02	94	2.78E+08	133 (73)	Carbamidomethylation; Oxidation (M); Deamidation (NQ)	24409	Trypsin OS=Sus scrofa PE=1 SV=1
Q86YZ3	259.72	21	7.94E+06	102 (100)	Deamidation (NQ)	282389	Hornerin OS=Homo sapiens GN=HRNR PE=1 SV=2
P13647	233.36	60	5.77E+06	71 (13)	Carbamidomethylation; Oxidation (M); Deamidation (NQ)	62378	Keratin type II cytoskeletal 5 OS=Homo sapiens GN=KRT5 PE=1 SV=3
P02533	230.68	73	1.22E+07	55 (10)	Carbamidomethylation; Oxidation (M); Deamidation (NQ)	51562	Keratin type I cytoskeletal 14 OS=Homo sapiens GN=KRT14 PE=1 SV=4
P04259	229.51	63	2.40E+05	66 (2)	Carbamidomethylation; Deamidation (NQ)	60067	Keratin type II cytoskeletal 6B OS=Homo sapiens GN=KRT6B PE=1 SV=5
O43790	227.9	59	3.34E+05	37 (3)	Carbamidomethylation; Oxidation (M)	53501	Keratin type II cuticular Hb6 OS=Homo sapiens GN=KRT86 PE=1 SV=1
P08779	223.77	74	8.50E+06	52 (22)	Carbamidomethylation; Oxidation (M); Deamidation (NQ)	51268	Keratin type I cytoskeletal 16 OS=Homo sapiens GN=KRT16 PE=1 SV=4
P15924	221.17	18	3.77E+06	49 (44)	Carbamidomethylation	331774	Desmoplakin OS=Homo sapiens GN=DSP PE=1 SV=3
Q6EIZ0	220.46	37	1.51E+06	52 (2)	Carbamidomethylation; Oxidation (M); Deamidation (NQ)	57711	Keratin type I cytoskeletal 10 OS=Canis lupus familiaris GN=KRT10 PE=2 SV=1
P78386	207.41	57	5.53E+05	35 (6)	Carbamidomethylation; Oxidation (M)	55802	Keratin type II cuticular Hb5 OS=Homo sapiens GN=KRT85 PE=1 SV=1
Q02SZ7	198.68	41	1.74E+07	28 (28)	Carbamidomethylation; Deamidation (NQ)	48178	Lysyl endopeptidase OS=Pseudomonas aeruginosa (strain UCBPP-PA14) OX=208963 GN=prpL PE=1 SV=1
Q9HWK6	198.68	41	1.74E+07	28 (28)	Carbamidomethylation; Deamidation (NQ)	48213	Lysyl endopeptidase OS=Pseudomonas aeruginosa (strain ATCC 15692 / DSM 22644 / CIP 104116 / JCM 14847 / LMG 12228 / 1C / PRS 101 / PAO1) OX=208964 GN=prpL PE=1 SV=1
P02768	196.68	49	3.51E+07	44 (26)	Carbamidomethylation	69367	Serum albumin OS=Homo sapiens GN=ALB PE=1 SV=2
Q15323	195.05	60	2.46E+05	25 (3)	Carbamidomethylation	47237	Keratin type I cuticular Ha1 OS=Homo sapiens GN=KRT31 PE=1 SV=3
A5A6M5	195.05	60	2.46E+05	25 (3)	Carbamidomethylation	47247	Keratin type I cuticular Ha1 OS=Pan troglodytes GN=KRT31 PE=2 SV=1
Q8NHM4	192.17	37	1.26E+06	34 (1)	Carbamidomethylation; Deamidation (NQ)	26537	Putative trypsin-6 OS=Homo sapiens GN=PRSS3P2 PE=5 SV=2
Q04695	188.6	62	1.28E+06	38 (5)	Carbamidomethylation; Oxidation (M); Deamidation (NQ)	48106	Keratin type I cytoskeletal 17 OS=Homo sapiens GN=KRT17 PE=1 SV=2
P02788	183.83	34	1.35E+06	24 (20)	Carbamidomethylation	78182	Lactotransferrin OS=Homo sapiens GN=LTF PE=1 SV=6
Q14525	182.13	50	6.01E+04	20 (2)	Carbamidomethylation	46214	Keratin type I cuticular Ha3-II OS=Homo sapiens GN=KRT33B PE=1 SV=3
P04280	178.59	65	9.41E+06	28 (15)	Deamidation (NQ)	38546	Basic salivary proline-rich protein 1 OS=Homo sapiens GN=PRB1 PE=1 SV=2

Accession	-10lgP	Coverage (%)	Area	#Peptides (#unique)	PTM	Avg. Mass	Description
P19013	174.91	36	4.59E+05	27 (8)	Carbamidomethylation	57285	Keratin type II cytoskeletal 4 OS=Homo sapiens GN=KRT4 PE=1 SV=4
Q6EIZ1	173.35	27	9.00E+05	38 (3)	Carbamidomethylation; Deamidation (NQ)	64575	Keratin type II cytoskeletal 2 epidermal OS=Canis lupus familiaris GN=KRT2 PE=2 SV=1
O76011	169.78	42	2.10E+05	19 (2)	Carbamidomethylation	49424	Keratin type I cuticular Ha4 OS=Homo sapiens GN=KRT34 PE=1 SV=2
P01833	167.23	20	2.20E+06	23 (23)	Carbamidomethylation	83284	Polymeric immunoglobulin receptor OS=Homo sapiens GN=PIGR PE=1 SV=4
Q6IG02	163.11	22	2.99E+05	27 (3)	Carbamidomethylation; Oxidation (M)	69127	Keratin type II cytoskeletal 2 epidermal OS=Rattus norvegicus GN=Krt2 PE=3 SV=1
O18330	162.77	45	1.21E+07	20 (17)	Carbamidomethylation; Oxidation (M); Deamidation (NQ)	48886	Major royal jelly protein 1 OS=Apis mellifera OX=7460 GN=MRJP1 PE=1 SV=1
P02812	162.33	22	8.63E+04	19 (2)	Deamidation (NQ)	40799	Basic salivary proline-rich protein 2 OS=Homo sapiens GN=PRB2 PE=1 SV=3
Q08D91	159.73	27	0.00E+00	34 (1)	Carbamidomethylation	59036	Keratin type II cytoskeletal 75 OS=Bos taurus GN=KRT75 PE=2 SV=1
Q02413	159.11	19	2.02E+06	14 (14)	Carbamidomethylation; Oxidation (M)	113748	Desmoglein-1 OS=Homo sapiens GN=DSG1 PE=1 SV=2
P81605	157.93	72	2.31E+07	19 (18)	Carbamidomethylation	11284	Dermcidin OS=Homo sapiens GN=DCD PE=1 SV=2
Q8SPJ1	155.98	23	1.40E+06	17 (17)	Carbamidomethylation; Oxidation (M)	81821	Junction plakoglobin OS=Bos taurus GN=JUP PE=2 SV=1
P14923	155.98	23	1.40E+06	17 (17)	Carbamidomethylation; Oxidation (M)	81745	Junction plakoglobin OS=Homo sapiens GN=JUP PE=1 SV=3
Q8WNW3	155.98	23	1.40E+06	17 (17)	Carbamidomethylation; Oxidation (M)	81850	Junction plakoglobin OS=Sus scrofa GN=Jup PE=2 SV=1
Q02257	155.98	23	1.40E+06	17 (17)	Carbamidomethylation; Oxidation (M)	81801	Junction plakoglobin OS=Mus musculus GN=Jup PE=1 SV=3
P08426	153.73	31	2.24E+06	23 (2)	Carbamidomethylation; Deamidation (NQ)	26269	Cationic trypsin-3 OS=Rattus norvegicus GN=Try3 PE=2 SV=1
Q17060	149.86	32	2.15E+06	15 (13)	Carbamidomethylation; Oxidation (M); Deamidation (NQ)	61662	Major royal jelly protein 3 OS=Apis mellifera OX=7460 GN=MRJP3 PE=1 SV=1
P10163	149.07	42	3.09E+06	25 (16)		31326	Basic salivary proline-rich protein 4 OS=Homo sapiens GN=PRB4 PE=1 SV=4
P61628	146.79	61	5.35E+06	15 (5)	Carbamidomethylation	16537	Lysozyme C OS=Pan troglodytes GN=LYZ PE=2 SV=1
P61626	146.79	61	5.35E+06	15 (5)	Carbamidomethylation	16537	Lysozyme C OS=Homo sapiens GN=LYZ PE=1 SV=1
P79179	146.79	61	5.35E+06	15 (5)	Carbamidomethylation	16567	Lysozyme C OS=Gorilla gorilla gorilla GN=LYZ PE=2 SV=1
P61627	146.79	61	5.35E+06	15 (5)	Carbamidomethylation	16537	Lysozyme C OS=Pan paniscus GN=LYZ PE=3 SV=1
P13646	146.05	26	9.02E+04	18 (2)	Carbamidomethylation; Deamidation (NQ)	49588	Keratin type I cytoskeletal 13 OS=Homo sapiens GN=KRT13 PE=1 SV=4
P12273	145.55	64	3.73E+06	10 (8)	Carbamidomethylation	16572	Prolactin-inducible protein OS=Homo sapiens GN=PIP PE=1 SV=1
P00762	145.45	34	2.36E+05	23 (2)	Carbamidomethylation; Oxidation (M); Deamidation (NQ)	25959	Anionic trypsin-1 OS=Rattus norvegicus GN=Prss1 PE=1 SV=1
Q7Z794	145.11	17	6.63E+04	23 (4)	Oxidation (M); Deamidation (NQ)	61901	Keratin type II cytoskeletal 1b OS=Homo sapiens GN=KRT77 PE=1 SV=3
P0DJG0	144.89	61	1.67E+06	16 (16)		30778	Apolipoprotein A-I OS=Pan troglodytes GN=APOA1 PE=1 SV=1
P02647	144.89	61	1.67E+06	16 (16)		30778	Apolipoprotein A-I OS=Homo sapiens GN=APOA1 PE=1 SV=1
G3QY98	144.89	61	1.67E+06	16 (16)		30778	Apolipoprotein A-I OS=Gorilla gorilla gorilla GN=APOA1 PE=1 SV=1
P00760	143.02	40	5.76E+06	22 (6)	Carbamidomethylation; Deamidation (NQ)	25785	Cationic trypsin OS=Bos taurus PE=1 SV=3

Accession	-10lgP	Coverage (%)	Area	#Peptides (#unique)	PTM	Avg. Mass	Description
P01876	138.51	24	1.01E+06	8 (4)		37655	Immunoglobulin heavy constant alpha 1 OS=Homo sapiens GN=IGHA1 PE=1 SV=2
Q8N1N4	138.46	25	1.42E+05	20 (6)	Carbamidomethylation	56866	Keratin type II cytoskeletal 78 OS=Homo sapiens GN=KRT78 PE=1 SV=2
P06872	138.38	23	5.52E+05	22 (3)	Carbamidomethylation	26423	Anionic trypsin OS=Canis lupus familiaris PE=2 SV=1
Q04118	137.98	43	8.61E+06	21 (17)		30980	Basic salivary proline-rich protein 3 OS=Homo sapiens GN=PRB3 PE=1 SV=2
Q8TDL5	133.06	26	2.80E+05	10 (10)		52442	BPI fold-containing family B member 1 OS=Homo sapiens GN=BPIFB1 PE=1 SV=1
P04406	131.97	30	1.10E+06	10 (10)	Carbamidomethylation; Deamidation (NQ)	36053	Glyceraldehyde-3-phosphate dehydrogenase OS=Homo sapiens GN=GAPDH PE=1 SV=3
O77061	129.66	25	1.03E+06	8 (5)	Carbamidomethylation; Oxidation (M); Deamidation (NQ)	51074	Major royal jelly protein 2 OS=Apis mellifera OX=7460 GN=MRJP2 PE=1 SV=1
P31025	123.89	44	1.40E+06	9 (9)	Carbamidomethylation	19250	Lipocalin-1 OS=Homo sapiens GN=LCN1 PE=1 SV=1
Q6UWP8	123.13	23	2.95E+05	10 (10)		60541	Suprabasin OS=Homo sapiens GN=SBSN PE=1 SV=2
P0AEX9	122.7	22	3.22E+06	10 (10)		43388	Maltose/maltodextrin-binding periplasmic protein OS=Escherichia coli (strain K12) OX=83333 GN=malE PE=1 SV=1
P0AEY0	122.7	22	3.22E+06	10 (10)		43388	Maltose/maltodextrin-binding periplasmic protein OS=Escherichia coli O157:H7 OX=83334 GN=malE PE=1 SV=1
P01040	121.93	74	1.24E+06	7 (7)		11006	Cystatin-A OS=Homo sapiens GN=CSTA PE=1 SV=1
O76015	117.78	18	6.86E+04	8 (5)	Carbamidomethylation	50480	Keratin type I cuticular Ha8 OS=Homo sapiens GN=KRT38 PE=1 SV=3
P79180	117.58	47	4.47E+03	11 (1)	Carbamidomethylation; Deamidation (NQ)	16676	Lysozyme C OS=Hylobates lar GN=LYZ PE=2 SV=1
P05109	117.02	77	8.97E+05	8 (7)	Carbamidomethylation; Oxidation (M)	10835	Protein S100-A8 OS=Homo sapiens GN=S100A8 PE=1 SV=1
Q5T749	116.07	30	6.95E+05	13 (13)	Carbamidomethylation	64136	Keratinocyte proline-rich protein OS=Homo sapiens GN=KPRP PE=1 SV=1
P02671	115.47	11	3.70E+05	9 (9)		94973	Fibrinogen alpha chain OS=Homo sapiens GN=FGA PE=1 SV=2
P04745	114.08	16	1.58E+05	6 (6)		57768	Alpha-amylase 1 OS=Homo sapiens GN=AMY1A PE=1 SV=2
Q08554	110.61	10	8.92E+05	7 (17)	Carbamidomethylation	99987	Desmocollin-1 OS=Homo sapiens GN=DSC1 PE=1 SV=2
Q96DA0	105.92	27	1.68E+06	5 (5)	Oxidation (M)	22739	Zymogen granule protein 16 homolog B OS=Homo sapiens GN=ZG16B PE=1 SV=3
P0DOY2	105.81	53	1.09E+06	5 (5)		11294	Immunoglobulin lambda constant 2 OS=Homo sapiens GN=IGLC2 PE=1 SV=1
P0DOY3	105.81	53	1.09E+06	5 (5)		11266	Immunoglobulin lambda constant 3 OS=Homo sapiens GN=IGLC3 PE=1 SV=1
P31151	105.74	37	3.92E+05	5 (5)	Carbamidomethylation; Deamidation (NQ)	11471	Protein S100-A7 OS=Homo sapiens GN=S100A7 PE=1 SV=4
Q5REL2	105.25	16	8.03E+04	5 (5)		38700	Annexin A1 OS=Pongo abelii GN=ANXA1 PE=2 SV=1
A5A6M2	105.25	16	8.03E+04	5 (5)		38742	Annexin A1 OS=Pan troglodytes GN=ANXA1 PE=2 SV=1
P04083	105.25	16	8.03E+04	5 (5)		38714	Annexin A1 OS=Homo sapiens GN=ANXA1 PE=1 SV=2
Q9NP55	103.03	21	4.40E+05	4 (4)		26713	BPI fold-containing family A member 1 OS=Homo sapiens GN=BPIFA1 PE=1 SV=1
O97432	102.35	15	2.99E+05	8 (5)	Oxidation (M)	70236	Major royal jelly protein 5 OS=Apis mellifera OX=7460 GN=MRJP5 PE=2 SV=1
P0DOX5	102.04	23	1.23E+06	7 (6)	Carbamidomethylation	49330	Immunoglobulin gamma-1 heavy chain OS=Homo sapiens PE=1 SV=1

Accession	-10lgP	Coverage (%)	Area	#Peptides (#unique)	PTM	Avg. Mass	Description
P01857	102.04	32	1.23E+06	7 (6)	Carbamidomethylation	36106	Immunoglobulin heavy constant gamma 1 OS=Homo sapiens GN=IGHG1 PE=1 SV=1
P07356	101.67	18	1.13E+05	6 (3)		38676	Annexin A2 OS=Mus musculus GN=Anxa2 PE=1 SV=2
P19620	101.67	18	1.13E+05	6 (3)		38542	Annexin A2 OS=Sus scrofa GN=ANXA2 PE=1 SV=4
Q6TEQ7	101.67	18	1.13E+05	6 (3)		38654	Annexin A2 OS=Canis lupus familiaris GN=ANXA2 PE=1 SV=1
P05089	101.26	19	1.92E+05	5 (5)		34735	Arginase-1 OS=Homo sapiens GN=ARG1 PE=1 SV=2
P0DOX2	101.02	11	1.60E+04	6 (2)		48935	Immunoglobulin alpha-2 heavy chain OS=Homo sapiens PE=1 SV=1
P25311	98.95	24	1.57E+05	6 (6)	Carbamidomethylation	34259	Zinc-alpha-2-glycoprotein OS=Homo sapiens GN=AZGP1 PE=1 SV=2
Q7YR44	98.73	13	7.92E+05	6(6)	Carbamidomethylation	51611	Corneodesmosin OS=Pan troglodytes GN=CDSN PE=2 SV=1
Q9NZT1	97.54	25	5.24E+05	4 (4)		15893	Calmodulin-like protein 5 OS=Homo sapiens GN=CALML5 PE=1 SV=2
P31944	95.06	20	4.55E+05	5 (5)	Carbamidomethylation	27680	Caspase-14 OS=Homo sapiens GN=CASP14 PE=1 SV=2
Q9GZZ8	94.78	35	8.40E+05	6 (6)	Oxidation (M); Deamidation (NQ)	14246	Extracellular glycoprotein lacritin OS=Homo sapiens GN=LACRT PE=1 SV=1
P23490	94.77	39	1.14E+05	7 (7)	Carbamidomethylation	25761	Loricrin OS=Homo sapiens GN=LOR PE=1 SV=2
P02810	93.83	61	1.27E+06	8 (8)		17016	Salivary acidic proline-rich phosphoprotein 1/2 OS=Homo sapiens GN=PRH1 PE=1 SV=2
P06702	86.5	31	9.15E+05	3 (3)		13242	Protein S100-A9 OS=Homo sapiens GN=S100A9 PE=1 SV=1
O18740	82.68	13	1.32E+04	10 (2)	Deamidation (NQ)	76354	Keratin type I cytoskeletal 9 OS=Canis lupus familiaris GN=KRT9 PE=3 SV=1
P03973	76.9	30	1.53E+05	5 (5)	Carbamidomethylation	14326	Antileukoproteinase OS=Homo sapiens GN=SLPI PE=1 SV=2
Q01469	75.81	22	2.09E+05	4 (4)	Carbamidomethylation	15164	Fatty acid-binding protein epidermal OS=Homo sapiens GN=FABP5 PE=1 SV=3
Q9UBC9	70.01	24	5.38E+04	4 (4)	Carbamidomethylation	18154	Small proline-rich protein 3 OS=Homo sapiens GN=SPRR3 PE=1 SV=2
Q75VN4	63.03	21	3.55E+04	4 (4)		13906	Histone H2B OS=Rhacophorus schlegelii GN=histh2b PE=1 SV=3
P02284	63.03	21	3.55E+04	4 (4)		13525	Histone H2B gonadal OS=Patella granatina PE=1 SV=2
P30757	63.03	21	3.55E+04	4 (4)		13664	Histone H2B OS=Sipunculus nudus GN=H2B PE=1 SV=2
P59782	63.03	21	3.55E+04	4 (4)		13696	Histone H2B OS=Drosophila simulans GN=His2B PE=3 SV=2
Q76FD7	63.03	21	3.55E+04	4 (4)		13709	Histone H2B OS=Drosophila sechellia GN=His2B PE=3 SV=3
P19374	63.03	21	3.55E+04	4 (4)		13638	Histone H2B OS=Platynereis dumerilii PE=3 SV=2
Q76FE9	63.03	21	3.55E+04	4 (4)		13696	Histone H2B OS=Drosophila orena GN=His2B PE=3 SV=3
P27326	63.03	21	3.55E+04	4 (4)		13737	Histone H2B OS=Urechis caupo PE=3 SV=2
Q76FF3	63.03	21	3.55E+04	4 (4)		13696	Histone H2B OS=Drosophila teissieri GN=His2B PE=3 SV=3
Q8I1N0	63.03	21	3.55E+04	4 (4)		13724	Histone H2B OS=Drosophila yakuba GN=His2B PE=3 SV=3
P02283	63.03	21	3.55E+04	4 (4)		13696	Histone H2B OS=Drosophila melanogaster GN=His2B PE=1 SV=2

Accession	-10lgP	Coverage (%)	Area	#Peptides (#unique)	PTM	Avg. Mass	Description
P69069	63.03	21	3.55E+04	4 (4)		13596	Histone H2B OS=Oncorhynchus mykiss PE=1 SV=2
P69070	63.03	21	3.55E+04	4 (4)		13596	Histone H2B OS=Salmo trutta PE=1 SV=2
P83863	63.03	22	3.55E+04	4 (4)		12775	Histone H2B (Fragments) OS=Litopenaeus vannamei PE=1 SV=2
Q9BYE4	62.85	30	3.29E+05	2 (2)	Carbamidomethylation	8158	Small proline-rich protein 2G OS=Homo sapiens GN=SPRR2G PE=3 SV=1
P22532	62.85	31	3.29E+05	2 (2)	Carbamidomethylation	7905	Small proline-rich protein 2D OS=Homo sapiens GN=SPRR2D PE=2 SV=2
P35325	62.85	31	3.29E+05	2 (2)	Carbamidomethylation	7975	Small proline-rich protein 2B OS=Homo sapiens GN=SPRR2B PE=2 SV=1
P35326	62.85	31	3.29E+05	2 (2)	Carbamidomethylation	7965	Small proline-rich protein 2A OS=Homo sapiens GN=SPRR2A PE=1 SV=1
P22531	62.85	31	3.29E+05	2 (2)	Carbamidomethylation	7855	Small proline-rich protein 2E OS=Homo sapiens GN=SPRR2E PE=2 SV=2
Q2PFZ3	61.01	15	2.14E+04	2 (2)		21892	Peroxiredoxin-2 OS=Macaca fascicularis GN=PRDX2 PE=2 SV=3
P32119	61.01	15	2.14E+04	2 (2)		21892	Peroxiredoxin-2 OS=Homo sapiens GN=PRDX2 PE=1 SV=5
Q8K3U7	61.01	15	2.14E+04	2 (2)		21813	Peroxiredoxin-2 OS=Cricetulus griseus GN=PRDX2 PE=2 SV=3
Q5RC63	61.01	16	2.14E+04	2 (2)		19430	Peroxiredoxin-2 OS=Pongo abelii GN=PRDX2 PE=2 SV=3
P35321	60.6	43	2.11E+05	4 (4)	Carbamidomethylation	9877	Cornifin-A OS=Homo sapiens GN=SPRR1A PE=1 SV=2
P22528	60.6	43	2.11E+05	4 (4)	Carbamidomethylation	9888	Cornifin-B OS=Homo sapiens GN=SPRR1B PE=1 SV=2
P17785	57.48	10	5.07E+03	4 (4)		38640	Annexin A2 OS=Gallus gallus GN=ANXA2 PE=1 SV=2
P02264	56.72	12	7.47E+04	2 (2)		13731	Histone H2A OS=Oncorhynchus mykiss PE=1 SV=2
P0CN99	56.72	12	7.47E+04	2 (2)		13914	Histone H2A OS=Cryptococcus neoformans var. neoformans serotype D (strain B-3501A) GN=HTA1 PE=3 SV=1
P0CN98	56.72	12	7.47E+04	2 (2)		13914	Histone H2A OS=Cryptococcus neoformans var. neoformans serotype D (strain JEC21 / ATCC MYA-565) GN=HTA1 PE=3 SV=1
P40282	56.72	12	7.47E+04	2 (2)		14122	Histone H2A OS=Plasmodium falciparum PE=2 SV=1
Q4PEF9	56.72	12	7.47E+04	2 (2)		14199	Histone H2A OS=Ustilago maydis (strain 521 / FGSC 9021) GN=HTA1 PE=3 SV=3
Q6PV61	56.72	13	7.47E+04	2 (2)		13267	Histone H2A OS=Litopenaeus vannamei PE=1 SV=3
P84052	56.72	13	7.47E+04	2 (2)		13363	Histone H2A OS=Drosophila erecta GN=His2A PE=3 SV=2
P84055	56.72	13	7.47E+04	2 (2)		13363	Histone H2A OS=Drosophila yakuba GN=His2A PE=2 SV=2
P84056	56.72	13	7.47E+04	2 (2)		13363	Histone H2A OS=Rhynchosciara americana GN=His2A PE=3 SV=2
P84053	56.72	13	7.47E+04	2 (2)		13363	Histone H2A OS=Drosophila hydei GN=His2A PE=3 SV=2
P69141	56.72	13	7.47E+04	2 (2)		13332	Histone H2A embryonic OS=Strongylocentrotus purpuratus PE=1 SV=2
P84054	56.72	13	7.47E+04	2 (2)		13363	Histone H2A OS=Drosophila simulans GN=His2A PE=3 SV=2
P13630	56.72	13	7.47E+04	2 (2)		13304	Histone H2A OS=Paracentrotus lividus PE=3 SV=2
P19178	56.72	13	7.47E+04	2 (2)		13310	Histone H2A OS=Platynereis dumerilii PE=3 SV=2

Accession	-10lgP	Coverage (%)	Area	#Peptides (#unique)	PTM	Avg. Mass	Description
P02270	56.72	13	7.47E+04	2 (2)		13353	Histone H2A OS=Sipunculus nudus PE=1 SV=2
P84057	56.72	13	7.47E+04	2 (2)		13363	Histone H2A OS=Tigriopus californicus GN=His2A PE=3 SV=2
P84051	56.72	13	7.47E+04	2 (2)		13363	Histone H2A OS=Drosophila melanogaster GN=His2A PE=1 SV=2
P04736	56.72	13	7.47E+04	2 (2)		13199	Late histone H2A.2.1 OS=Psammechinus miliaris PE=2 SV=2
Q6WV69	56.72	13	7.47E+04	2 (2)		13360	Histone H2A OS=Mytilus chilensis PE=3 SV=3
Q6WV66	56.72	13	7.47E+04	2 (2)		13360	Histone H2A OS=Mytilus californianus PE=3 SV=3
P02268	56.72	13	7.47E+04	2 (2)		13421	Histone H2A OS=Sepia officinalis PE=1 SV=2
Q6WV67	56.72	13	7.47E+04	2 (2)		13288	Histone H2A OS=Mytilus trossulus PE=3 SV=3
P27325	56.72	13	7.47E+04	2 (2)		13440	Histone H2A OS=Urechis caupo PE=3 SV=2
Q8I0T3	56.72	13	7.47E+04	2 (2)		13360	Histone H2A OS=Mytilus edulis PE=3 SV=3
Q6WV88	56.72	13	7.47E+04	2 (2)		13360	Histone H2A OS=Mytilus galloprovincialis PE=3 SV=3
P69140	56.72	13	7.47E+04	2 (2)		13411	Histone H2A gonadal OS=Parechinus angulosus PE=1 SV=2
P62972	54.31	15	2.97E+05	2 (2)		18801	Polyubiquitin (Fragment) OS=Xenopus laevis PE=1 SV=2
P62975	54.31	33	2.97E+05	2 (2)		8565	Ubiquitin OS=Oryctolagus cuniculus PE=1 SV=1
P68197	54.31	33	2.97E+05	2 (2)		8565	Ubiquitin OS=Ceratitis capitata PE=1 SV=1
Q865C5	54.31	33	2.97E+05	2 (2)		8565	Ubiquitin OS=Camelus dromedarius PE=3 SV=2
P84589	54.31	40	2.97E+05	2 (2)		7203	Ubiquitin (Fragment) OS=Lumbricus terrestris PE=1 SV=2
Q9BYQ8	49.49	10	4.64E+04	2 (2)	Carbamidomethylation	22405	Keratin-associated protein 4-9 OS=Homo sapiens GN=KRTAP4-9 PE=2 SV=2
P62783	48.81	19	1.12E+04	2 (2)		11369	Histone H4 OS=Strongylocentrotus purpuratus PE=3 SV=2
P84049	48.81	19	1.12E+04	2 (2)		11381	Histone H4 OS=Myrmica ruginodis GN=His4 PE=3 SV=2
P62780	48.81	19	1.12E+04	2 (2)		11369	Histone H4 OS=Paracentrotus lividus PE=3 SV=2
P84045	48.81	19	1.12E+04	2 (2)		11381	Histone H4 OS=Tigriopus californicus GN=His4 PE=3 SV=2
P82888	48.81	19	1.12E+04	2 (2)		11423	Histone H4 OS=Olisthodiscus luteus PE=1 SV=2
Q6WV73	48.81	19	1.12E+04	2 (2)		11367	Histone H4 OS=Mytilus californianus PE=3 SV=3
Q5RCS7	48.81	19	1.12E+04	2 (2)		11367	Histone H4 OS=Pongo abelii PE=3 SV=1
P62781	48.81	19	1.12E+04	2 (2)		11369	Histone H4 OS=Psammechinus miliaris PE=1 SV=2
Q28DR4	48.81	19	1.12E+04	2 (2)		11367	Histone H4 OS=Xenopus tropicalis GN=TGas006m08.1 PE=3 SV=1
Q43083	48.81	19	1.12E+04	2 (2)		11425	Histone H4 OS=Pyrenomonas salina PE=3 SV=3
P62778	48.81	19	1.12E+04	2 (2)		11369	Histone H4 OS=Pisaster ochraceus PE=3 SV=2
P62804	48.81	19	1.12E+04	2 (2)		11367	Histone H4 OS=Rattus norvegicus GN=Hist1h4b PE=1 SV=2

Accession	-10lgP	Coverage (%)	Area	#Peptides (#unique)	PTM	Avg. Mass	Description
P62795	48.81	19	1.12E+04	2 (2)		11367	Histone H4 OS=Platynereis dumerilii PE=3 SV=2
P84050	48.81	19	1.12E+04	2 (2)		11381	Histone H4 OS=Rhynchosciara americana GN=His4 PE=3 SV=2
P62797	48.81	19	1.12E+04	2 (2)		11367	Histone H4 OS=Oncorhynchus mykiss PE=1 SV=2
P62796	48.81	19	1.12E+04	2 (2)		11367	Histone H4 OS=Oreochromis niloticus PE=3 SV=2
P62802	48.81	19	1.12E+04	2 (2)		11367	Histone H4 OS=Sus scrofa PE=1 SV=2
P62799	48.81	19	1.12E+04	2 (2)		11367	Histone H4 OS=Xenopus laevis PE=1 SV=2
Q6WV90	48.81	19	1.12E+04	2 (2)		11367	Histone H4 OS=Mytilus galloprovincialis PE=3 SV=3
Q8I0Y4	48.81	19	1.12E+04	2 (2)		11349	Histone H4 OS=Oikopleura dioica GN=H4.1 PE=3 SV=3
P27996	48.81	19	1.12E+04	2 (2)		11385	Histone H4 OS=Solaster stimpsoni PE=3 SV=2
P62794	48.81	19	1.12E+04	2 (2)		11367	Histone H4 OS=Urechis caupo PE=3 SV=2
P62777	48.81	19	1.12E+04	2 (2)		11369	Histone H4 OS=Pisaster brevispinus PE=3 SV=2
P91890	48.81	19	1.12E+04	2 (2)		11337	Histone H4 OS=Trichogramma cacaeciae PE=3 SV=3
Q6WV74	48.81	19	1.12E+04	2 (2)		11395	Histone H4 OS=Mytilus chilensis PE=3 SV=3
Q27765	48.81	19	1.12E+04	2 (2)		11349	Histone H4 OS=Styela plicata PE=3 SV=3
P62798	48.81	19	1.12E+04	2 (2)		11367	Histone H4 OS=Xenopus borealis PE=3 SV=2
Q6WV72	48.81	19	1.12E+04	2 (2)		11367	Histone H4 OS=Mytilus trossulus PE=3 SV=3
P62779	48.81	19	1.12E+04	2 (2)		11369	Histone H4 OS=Pycnopodia helianthoides PE=3 SV=2
Q7K8C0	48.81	19	1.12E+04	2 (2)		11367	Histone H4 OS=Mytilus edulis PE=3 SV=3
Q5T750	47.15	15	1.91E+05	3 (3)	Carbamidomethylation	26238	Skin-specific protein 32 OS=Homo sapiens GN=XP32 PE=1 SV=1
Q7SY65	47.12	11	4.41E+05	4 (2)	Deamidation (NQ)	48004	Keratin type I cytoskeletal 18-B OS=Xenopus laevis GN=krt18-b PE=2 SV=1
Q8MIQ5	47.03	11	9.11E+04	2 (2)		11220	Apolipoprotein A-II OS=Pan troglodytes GN=APOA2 PE=1 SV=1
P0DM95	47.03	11	9.11E+04	2 (2)		11220	Apolipoprotein A-II OS=Pan paniscus GN=APOA2 PE=1 SV=1
P02652	47.03	11	9.11E+04	2 (2)		11175	Apolipoprotein A-II OS=Homo sapiens GN=APOA2 PE=1 SV=1
P0DJG2	47.03	11	9.11E+04	2 (2)		11175	Apolipoprotein A-II OS=Gorilla gorilla gorilla GN=APOA2 PE=1 SV=1
P0DJD2	47.03	12	9.11E+04	2 (2)		10516	Apolipoprotein A-II OS=Pongo abelii GN=APOA2 PE=1 SV=1

(only matches with at least 2 unique peptides and above 10% coverage are listed)

Table S 18- Protein matches obtained for Honeycomb Greece Aged against Uniprot Honey Database

Accession	-10lgP	Coverage (%)	Area	#Peptides (#unique)	PTM	Avg. Mass	Description
O18330	218.73	45	1.19E+07	19 (17)	Carbamidomethylation; Deamidation (NQ); Oxidation (M)	48886	Major royal jelly protein 1 OS=Apis mellifera OX=7460 GN=MRJP1 PE=1 SV=1
Q17060	193.44	24	2.02E+06	14 (1)	Carbamidomethylation; Deamidation (NQ); Oxidation (M)	61662	Major royal jelly protein 3 OS=Apis mellifera OX=7460 GN=MRJP3 PE=1 SV=1
O77061	173.95	25	9.43E+05	8 (4)	Carbamidomethylation; Deamidation (NQ); Oxidation (M)	51074	Major royal jelly protein 2 OS=Apis mellifera OX=7460 GN=MRJP2 PE=1 SV=1
A0A1V9XF46	136.15	23	3.38E+05	6 (2)		41839	Actin-5C-like OS=Tropilaelaps mercedesae OX=418985 GN=BIW11_10578 PE=3 SV=1
A0A2A3EM69	136.15	23	3.38E+05	6 (2)		41806	Actin-5C OS=Apis cerana cerana OX=94128 GN=APICC_05085 PE=3 SV=1
A0A2A3E2E7	121.39	13	3.48E+04	5 (1)	Deamidation (NQ)	45603	Actin clone OS=Apis cerana cerana OX=94128 GN=APICC_04920 PE=3 SV=1
O97432	105.86	12	2.91E+05	6 (4)	Oxidation (M)	70236	Major royal jelly protein 5 OS=Apis mellifera OX=7460 GN=MRJP5 PE=2 SV=1
A0A1V9XXZ1	80.02	11	2.88E+04	4 (3)		28105	Histone H4 OS=Tropilaelaps mercedesae OX=418985 GN=BIW11_06453 PE=3 SV=1
A0A2A3EE69	53.78	18	1.31E+05	3 (3)		14289	Histone H2A OS=Apis cerana cerana OX=94128 GN=APICC_05173 PE=3 SV=1
T0MK29	45.98	21	6.00E+04	7 (7)	Deamidation (NQ)	44481	Uncharacterized protein OS=Nosema apis BRL 01 OX=1037528 GN=NAPIS_ORF01057 PE=4 SV=1
A0A1V9X9P2	45.52	10	3.14E+04	5 (5)	Deamidation (NQ)	40097	Transcription factor SOX-17-like OS=Tropilaelaps mercedesae OX=418985 GN=BIW11_01716 PE=4 SV=1
A0A2A3ECX5	38.68	11	3.19E+05	2 (2)	Carbamidomethylation	29815	Trypsin-2 OS=Apis cerana cerana OX=94128 GN=APICC_08270 PE=4 SV=1

(only matches with at least 5% coverage and 1 unique peptide are listed)

Table S 19 - Protein matches obtained for Honeycomb Italy Fresh against Uniprot All Proteins Database

Accession	-10lgP	Coverage (%)	Area	#Peptides (#unique)	PTM	Avg. Mass	Description
P35527	294.6	88	2.38E+08	132 (126)	Carbamidomethylation; Oxidation (M); Deamidation (NQ)	62064	Keratin type I cytoskeletal 9 OS=Homo sapiens OX=9606 GN=KRT9 PE=1 SV=3
P04264	259.45	84	1.22E+08	127 (76)	Carbamidomethylation; Oxidation (M); Deamidation (NQ)	66039	Keratin type II cytoskeletal 1 OS=Homo sapiens OX=9606 GN=KRT1 PE=1 SV=6
P00761	243.61	94	5.99E+07	95 (52)	Carbamidomethylation; Oxidation (M); Deamidation (NQ)	24409	Trypsin OS=Sus scrofa OX=9823 PE=1 SV=1
P35908	233.14	64	1.05E+07	55 (28)	Carbamidomethylation; Oxidation (M); Deamidation (NQ)	65433	Keratin type II cytoskeletal 2 epidermal OS=Homo sapiens OX=9606 GN=KRT2 PE=1 SV=2
P13645	232.68	70	4.76E+07	60 (22)	Carbamidomethylation; Oxidation (M); Deamidation (NQ)	58827	Keratin type I cytoskeletal 10 OS=Homo sapiens OX=9606 GN=KRT10 PE=1 SV=6
P02538	217.07	63	5.01E+05	63 (2)	Carbamidomethylation; Oxidation (M); Deamidation (NQ)	60045	Keratin type II cytoskeletal 6A OS=Homo sapiens OX=9606 GN=KRT6A PE=1 SV=3
P48668	214.27	73	1.46E+05	65 (4)	Carbamidomethylation; Oxidation (M); Deamidation (NQ)	60025	Keratin type II cytoskeletal 6C OS=Homo sapiens OX=9606 GN=KRT6C PE=1 SV=3
P23490	205.5	68	1.31E+06	30 (30)	Carbamidomethylation	25761	Loricrin OS=Homo sapiens OX=9606 GN=LORICRIN PE=1 SV=2
P02533	202.42	64	5.32E+06	45 (3)	Carbamidomethylation; Oxidation (M); Deamidation (NQ)	51562	Keratin type I cytoskeletal 14 OS=Homo sapiens OX=9606 GN=KRT14 PE=1 SV=4
P08779	199.1	77	5.76E+06	50 (22)	Carbamidomethylation; Oxidation (M); Deamidation (NQ)	51268	Keratin type I cytoskeletal 16 OS=Homo sapiens OX=9606 GN=KRT16 PE=1 SV=4
P13647	196.33	63	3.40E+06	55 (5)	Carbamidomethylation; Oxidation (M); Deamidation (NQ)	62378	Keratin type II cytoskeletal 5 OS=Homo sapiens OX=9606 GN=KRT5 PE=1 SV=3
Q6EIZ0	188.17	37	2.48E+05	38 (3)	Oxidation (M); Deamidation (NQ)	57711	Keratin type I cytoskeletal 10 OS=Canis lupus familiaris OX=9615 GN=KRT10 PE=2 SV=1
Q04695	184.6	64	2.19E+06	35 (5)	Carbamidomethylation; Oxidation (M); Deamidation (NQ)	48106	Keratin type I cytoskeletal 17 OS=Homo sapiens OX=9606 GN=KRT17 PE=1 SV=2
Q9HWK6	164.04	33	1.28E+07	15 (15)	Carbamidomethylation; Deamidation (NQ)	48213	Lysyl endopeptidase OS=Pseudomonas aeruginosa (strain ATCC 15692 / DSM 22644 / CIP 104116 / JCM 14847 / LMG 12228 / 1C / PRS 101 / PAO1) OX=208964 GN=prpL PE=1 SV=1
Q02SZ7	164.04	33	1.28E+07	15 (15)	Carbamidomethylation; Deamidation (NQ)	48178	Lysyl endopeptidase OS=Pseudomonas aeruginosa (strain UCBPP-PA14) OX=208963 GN=prpL PE=1 SV=1
P0AEX9	158.11	37	2.58E+06	14 (14)	Oxidation (M)	43388	Maltose/maltodextrin-binding periplasmic protein OS=Escherichia coli (strain K12) OX=83333 GN=malE PE=1 SV=1
P0AEY0	158.11	37	2.58E+06	14 (14)	Oxidation (M)	43388	Maltose/maltodextrin-binding periplasmic protein OS=Escherichia coli O157:H7 OX=83334 GN=malE PE=1 SV=1
P00760	154.55	33	3.86E+05	23 (5)	Carbamidomethylation; Deamidation (NQ)	25785	Cationic trypsin OS=Bos taurus OX=9913 PE=1 SV=3
P81605	135.02	55	6.67E+06	15 (15)	Carbamidomethylation	11284	Dermcidin OS=Homo sapiens OX=9606 GN=DCD PE=1 SV=2
O18330	134.22	29	1.56E+06	11 (11)	Oxidation (M)	48886	Major royal jelly protein 1 OS=Apis mellifera OX=7460 GN=MRJP1 PE=1 SV=1
Q02413	128.22	14	9.47E+05	13 (13)	Carbamidomethylation; Oxidation (M)	113748	Desmoglein-1 OS=Homo sapiens OX=9606 GN=DSG1 PE=1 SV=2

Accession	-10lgP	Coverage (%)	Area	#Peptides (#unique)	PTM	Avg. Mass	Description
Q8N1N4	128.01	22	8.89E+04	16 (7)	Carbamidomethylation	56866	Keratin type II cytoskeletal 78 OS=Homo sapiens OX=9606 GN=KRT78 PE=1 SV=2
P01009	123.13	30	5.63E+05	11 (11)	Deamidation (NQ)	46737	Alpha-1-antitrypsin OS=Homo sapiens OX=9606 GN=SERPINA1 PE=1 SV=3
Q04118	118.82	32	8.26E+05	9 (7)		30980	Basic salivary proline-rich protein 3 OS=Homo sapiens OX=9606 GN=PRB3 PE=1 SV=2
Q01469	111.79	48	3.59E+05	6 (6)	Carbamidomethylation	15164	Fatty acid-binding protein 5 OS=Homo sapiens OX=9606 GN=FABP5 PE=1 SV=3
P04406	108.31	25	2.41E+05	8 (7)	Carbamidomethylation; Deamidation (NQ)	36053	Glyceraldehyde-3-phosphate dehydrogenase OS=Homo sapiens OX=9606 GN=GAPDH PE=1 SV=3
Q96P63	107.28	20	1.73E+05	6 (6)	Carbamidomethylation	46276	Serpin B12 OS=Homo sapiens OX=9606 GN=SERPINB12 PE=1 SV=1
P12273	103.21	49	4.70E+05	6 (6)	Carbamidomethylation	16572	Prolactin-inducible protein OS=Homo sapiens OX=9606 GN=PIP PE=1 SV=1
Q6TEQ7	91.81	13	1.01E+05	4 (3)		38654	Annexin A2 OS=Canis lupus familiaris OX=9615 GN=ANXA2 PE=1 SV=1
Q5R5A0	91.81	13	1.01E+05	4 (3)		38604	Annexin A2 OS=Pongo abelii OX=9601 GN=ANXA2 PE=2 SV=1
P07355	91.81	13	1.01E+05	4 (3)		38604	Annexin A2 OS=Homo sapiens OX=9606 GN=ANXA2 PE=1 SV=2
P07356	91.81	13	1.01E+05	4 (3)		38676	Annexin A2 OS=Mus musculus OX=10090 GN=Anxa2 PE=1 SV=2
A6NMY6	91.81	13	1.01E+05	4 (3)		38659	Putative annexin A2-like protein OS=Homo sapiens OX=9606 GN=ANXA2P2 PE=5 SV=2
C0HJG9	91.81	22	1.01E+05	4 (3)		22447	Annexin A2 (Fragments) OS=Mesocricetus auratus OX=10036 PE=1 SV=1
P31944	90.51	22	1.27E+05	5 (5)		27680	Caspase-14 OS=Homo sapiens OX=9606 GN=CASP14 PE=1 SV=2
P91754	87.31	10	8.30E+04	3 (3)		41323	Actin (Fragment) OS=Lumbricus rubellus OX=35632 PE=2 SV=1
P10365	87.31	10	8.30E+04	3 (3)		41635	Actin OS=Thermomyces lanuginosus OX=5541 PE=3 SV=1
P50138	87.31	10	8.30E+04	3 (3)		41745	Actin OS=Puccinia graminis OX=5297 PE=3 SV=1
P49871	87.31	10	8.30E+04	3 (3)		41777	Actin muscle OS=Manduca sexta OX=7130 PE=2 SV=1
P17126	87.31	10	8.30E+04	3 (3)		41797	Actin non-muscle 6.2 OS=Hydra vulgaris OX=6087 PE=3 SV=1
O16808	87.31	10	8.30E+04	3 (3)		41817	Actin OS=Mayetiola destructor OX=39758 PE=2 SV=1
P68555	87.31	10	8.30E+04	3 (3)		41745	Actin OS=Taenia solium OX=6204 GN=ACT1 PE=3 SV=1
Q26065	87.31	10	8.30E+04	3 (3)		41762	Actin adductor muscle OS=Placopecten magellanicus OX=6577 PE=2 SV=1
O17320	87.31	10	8.30E+04	3 (3)		41792	Actin OS=Crassostrea gigas OX=29159 PE=2 SV=1
P06702	84.85	51	2.12E+05	5 (5)	Oxidation (M)	13242	Protein S100-A9 OS=Homo sapiens OX=9606 GN=S100A9 PE=1 SV=1
Q9NZT1	84.04	21	6.45E+05	2 (2)		15893	Calmodulin-like protein 5 OS=Homo sapiens OX=9606 GN=CALML5 PE=1 SV=2

Accession	-10lgP	Coverage (%)	Area	#Peptides (#unique)	PTM	Avg. Mass	Description
P04280	83.84	23	3.98E+04	7 (5)		38562	Basic salivary proline-rich protein 1 OS=Homo sapiens OX=9606 GN=PRB1 PE=1 SV=3
P01040	83.64	41	2.94E+05	4 (4)		11006	Cystatin-A OS=Homo sapiens OX=9606 GN=CSTA PE=1 SV=1
P35321	75.42	43	3.93E+05	4 (4)	Carbamidomethylation	9877	Cornifin-A OS=Homo sapiens OX=9606 GN=SPRR1A PE=1 SV=2
P22528	75.42	43	3.93E+05	4 (4)	Carbamidomethylation	9888	Cornifin-B OS=Homo sapiens OX=9606 GN=SPRR1B PE=1 SV=2
P02810	69.93	29	3.04E+05	4 (4)		17016	Salivary acidic proline-rich phosphoprotein 1/2 OS=Homo sapiens OX=9606 GN=PRH1 PE=1 SV=2
P25311	68.58	10	1.10E+05	3 (3)		34259	Zinc-alpha-2-glycoprotein OS=Homo sapiens OX=9606 GN=AZGP1 PE=1 SV=2
P31025	67.37	16	2.41E+05	3 (3)		19250	Lipocalin-1 OS=Homo sapiens OX=9606 GN=LCN1 PE=1 SV=1
Q5VSP4	67.37	18	2.41E+05	3 (3)		17918	Putative lipocalin 1-like protein 1 OS=Homo sapiens OX=9606 GN=LCN1P1 PE=5 SV=1
Q9BYE4	64.89	30	2.23E+05	2 (2)	Carbamidomethylation	8158	Small proline-rich protein 2G OS=Homo sapiens OX=9606 GN=SPRR2G PE=3 SV=1
P22531	64.89	31	2.23E+05	2 (2)	Carbamidomethylation	7855	Small proline-rich protein 2E OS=Homo sapiens OX=9606 GN=SPRR2E PE=2 SV=2
P22532	64.89	31	2.23E+05	2 (2)	Carbamidomethylation	7905	Small proline-rich protein 2D OS=Homo sapiens OX=9606 GN=SPRR2D PE=2 SV=2
P35326	64.89	31	2.23E+05	2 (2)	Carbamidomethylation	7965	Small proline-rich protein 2A OS=Homo sapiens OX=9606 GN=SPRR2A PE=1 SV=1
P35325	64.89	31	2.23E+05	2 (2)	Carbamidomethylation	7975	Small proline-rich protein 2B OS=Homo sapiens OX=9606 GN=SPRR2B PE=2 SV=1
P05109	64.79	24	9.95E+04	2 (2)	Carbamidomethylation	10835	Protein S100-A8 OS=Homo sapiens OX=9606 GN=S100A8 PE=1 SV=1
Q96FQ6	60.25	22	6.48E+04	2 (2)		11801	Protein S100-A16 OS=Homo sapiens OX=9606 GN=S100A16 PE=1 SV=1
P0DJD2	56.05	12	5.45E+04	2 (2)		10516	Apolipoprotein A-II OS=Pongo abelii OX=9601 GN=APOA2 PE=1 SV=1

(only matches with at least 2 unique peptides and 10% coverage are listed)

Table S 20 – Protein matches obtained for Honeycomb Italy Fresh against Uniprot Honey Database

Accession	-10lgP	Coverage (%)	Area	#Peptides (#unique)	PTM	Avg. Mass	Description
O18330	268.05	67	1.30E+08	43 (37)	Carbamidomethylation; Deamidation (NQ); Oxidation (M)	48886	Major royal jelly protein 1 OS=Apis mellifera OX=7460 GN=MRJP1 PE=1 SV=1
Q17060	257.37	58	3.90E+07	41 (34)	Carbamidomethylation; Deamidation (NQ); Oxidation (M)	61662	Major royal jelly protein 3 OS=Apis mellifera OX=7460 GN=MRJP3 PE=1 SV=1
O77061	242.75	52	1.58E+07	33 (21)	Carbamidomethylation; Deamidation (NQ); Oxidation (M)	51074	Major royal jelly protein 2 OS=Apis mellifera OX=7460 GN=MRJP2 PE=1 SV=1
O97432	176.49	25	3.01E+06	12 (9)	Oxidation (M)	70236	Major royal jelly protein 5 OS=Apis mellifera OX=7460 GN=MRJP5 PE=2 SV=1
A0A2A3E8C9	157.99	16	2.31E+06	10 (10)	Carbamidomethylation; Oxidation (M)	66292	Alpha-glucosidase OS=Apis cerana cerana OX=94128 GN=APICC_04097 PE=4 SV=1
Q17061	116.86	20	8.64E+05	12 (5)	Carbamidomethylation; Oxidation (M)	52916	Major royal jelly protein 4 OS=Apis mellifera OX=7460 GN=MRJP4 PE=1 SV=1
A0A2A3EG07	80.56	13	1.10E+05	8 (1)	Carbamidomethylation; Oxidation (M)	54971	Bee-milk protein OS=Apis cerana cerana OX=94128 GN=APICC_06207 PE=3 SV=1
A0A2A3ECX5	71.87	11	6.19E+05	6 (6)	Carbamidomethylation	29815	Trypsin-2 OS=Apis cerana cerana OX=94128 GN=APICC_08270 PE=4 SV=1
A0A1V9XCK9	42.47	35	2.01E+05	3 (3)	Deamidation (NQ)	9408	UPF0480 protein C15orf24-like OS=Tropilaelaps mercedesae OX=418985 GN=BIW11_11081 PE=4 SV=1
A0A2A3EPA8	31.40	11	1.96E+03	1 (1)	Carbamidomethylation	7942	Trypsin inhibitor isl-1 OS=Apis cerana cerana OX=94128 GN=APICC_08092 PE=4 SV=1
A0A2S8AEU0	23.67	10	2.14E+03	1 (1)	Carbamidomethylation	25326	Peptide methionine sulfoxide reductase MsrA OS=Apibacter adventoris OX=1679466 GN=msrA PE=3 SV=1

(only matches with at least 5% coverage and 1 unique peptide are listed)

Table S 21 – Protein matches obtained for Honeycomb Italy Aged against Uniprot All Proteins Database

Accession	-10lgP	Coverage (%)	Area	#Peptides (#unique)	PTM	Avg. Mass	Description
P35527	294.6	88	2.38E+08	132 (126)	Carbamidomethylation; Oxidation (M); Deamidation (NQ)	62064	Keratin type I cytoskeletal 9 OS=Homo sapiens OX=9606 GN=KRT9 PE=1 SV=3
P04264	259.45	84	1.22E+08	127 (76)	Carbamidomethylation; Oxidation (M); Deamidation (NQ)	66039	Keratin type II cytoskeletal 1 OS=Homo sapiens OX=9606 GN=KRT1 PE=1 SV=6
P00761	243.61	94	5.99E+07	95 (52)	Carbamidomethylation; Oxidation (M); Deamidation (NQ)	24409	Trypsin OS=Sus scrofa OX=9823 PE=1 SV=1
P35908	233.14	64	1.05E+07	55 (28)	Carbamidomethylation; Oxidation (M); Deamidation (NQ)	65433	Keratin type II cytoskeletal 2 epidermal OS=Homo sapiens OX=9606 GN=KRT2 PE=1 SV=2
P13645	232.68	70	4.76E+07	60 (22)	Carbamidomethylation; Oxidation (M); Deamidation (NQ)	58827	Keratin type I cytoskeletal 10 OS=Homo sapiens OX=9606 GN=KRT10 PE=1 SV=6
P02538	217.07	63	5.01E+05	63 (2)	Carbamidomethylation; Oxidation (M); Deamidation (NQ)	60045	Keratin type II cytoskeletal 6A OS=Homo sapiens OX=9606 GN=KRT6A PE=1 SV=3
P48668	214.27	73	1.46E+05	65 (4)	Carbamidomethylation; Oxidation (M); Deamidation (NQ)	60025	Keratin type II cytoskeletal 6C OS=Homo sapiens OX=9606 GN=KRT6C PE=1 SV=3
P23490	205.5	68	1.31E+06	30 (30)	Carbamidomethylation	25761	Loricrin OS=Homo sapiens OX=9606 GN=LORICRIN PE=1 SV=2
P02533	202.42	64	5.32E+06	45 (3)	Carbamidomethylation; Oxidation (M); Deamidation (NQ)	51562	Keratin type I cytoskeletal 14 OS=Homo sapiens OX=9606 GN=KRT14 PE=1 SV=4
P08779	199.1	77	5.76E+06	50 (22)	Carbamidomethylation; Oxidation (M); Deamidation (NQ)	51268	Keratin type I cytoskeletal 16 OS=Homo sapiens OX=9606 GN=KRT16 PE=1 SV=4
P13647	196.33	63	3.40E+06	55 (5)	Carbamidomethylation; Oxidation (M); Deamidation (NQ)	62378	Keratin type II cytoskeletal 5 OS=Homo sapiens OX=9606 GN=KRT5 PE=1 SV=3
Q6EIZ0	188.17	37	2.48E+05	38 (3)	Oxidation (M); Deamidation (NQ)	57711	Keratin type I cytoskeletal 10 OS=Canis lupus familiaris OX=9615 GN=KRT10 PE=2 SV=1
Q04695	184.6	64	2.19E+06	35 (5)	Carbamidomethylation; Oxidation (M); Deamidation (NQ)	48106	Keratin type I cytoskeletal 17 OS=Homo sapiens OX=9606 GN=KRT17 PE=1 SV=2
Q9HWK6	164.04	33	1.28E+07	15 (15)	Carbamidomethylation; Deamidation (NQ)	48213	Lysyl endopeptidase OS=Pseudomonas aeruginosa (strain ATCC 15692 / DSM 22644 / CIP 104116 / JCM 14847 / LMG 12228 / 1C / PRS 101 / PAO1) OX=208964 GN=prpL PE=1 SV=1
Q02SZ7	164.04	33	1.28E+07	15 (15)	Carbamidomethylation; Deamidation (NQ)	48178	Lysyl endopeptidase OS=Pseudomonas aeruginosa (strain UCBPP-PA14) OX=208963 GN=prpL PE=1 SV=1
P0AEX9	158.11	37	2.58E+06	14 (14)	Oxidation (M)	43388	Maltose/maltodextrin-binding periplasmic protein OS=Escherichia coli (strain K12) OX=83333 GN=malE PE=1 SV=1

Accession	-10lgP	Coverage (%)	Area	#Peptides (#unique)	PTM	Avg. Mass	Description
P0AEY0	158.11	37	2.58E+06	14 (14)	Oxidation (M)	43388	Maltose/maltodextrin-binding periplasmic protein OS=Escherichia coli O157:H7 OX=83334 GN=malE PE=1 SV=1
P00760	154.55	33	3.86E+05	23 (5)	Carbamidomethylation; Deamidation (NQ)	25785	Cationic trypsin OS=Bos taurus OX=9913 PE=1 SV=3
P14923	148.58	33	1.29E+06	20 (19)	Carbamidomethylation; Oxidation (M)	81745	Junction plakoglobin OS=Homo sapiens OX=9606 GN=JUP PE=1 SV=3
P81605	135.02	55	6.67E+06	15 (15)	Carbamidomethylation	11284	Dermcidin OS=Homo sapiens OX=9606 GN=DCD PE=1 SV=2
O18330	134.22	29	1.56E+06	11 (11)	Oxidation (M)	48886	Major royal jelly protein 1 OS=Apis mellifera OX=7460 GN=MRJP1 PE=1 SV=1
P02671	131.85	16	2.28E+05	11 (10)		94973	Fibrinogen alpha chain OS=Homo sapiens OX=9606 GN=FGA PE=1 SV=2
Q8N1N4	128.01	22	8.89E+04	16 (7)	Carbamidomethylation	56866	Keratin type II cytoskeletal 78 OS=Homo sapiens OX=9606 GN=KRT78 PE=1 SV=2
P01009	123.13	30	5.63E+05	11 (11)	Deamidation (NQ)	46737	Alpha-1-antitrypsin OS=Homo sapiens OX=9606 GN=SERPINA1 PE=1 SV=3
Q04118	118.82	32	8.26E+05	9 (7)		30980	Basic salivary proline-rich protein 3 OS=Homo sapiens OX=9606 GN=PRB3 PE=1 SV=2
P04406	108.31	25	2.41E+05	8 (7)	Carbamidomethylation; Deamidation (NQ)	36053	Glyceraldehyde-3-phosphate dehydrogenase OS=Homo sapiens OX=9606 GN=GAPDH PE=1 SV=3
Q96P63	107.28	20	1.73E+05	6 (6)	Carbamidomethylation	46276	Serpin B12 OS=Homo sapiens OX=9606 GN=SERPINB12 PE=1 SV=1
P00738	103.95	12	2.49E+05	6 (6)		45205	Haptoglobin OS=Homo sapiens OX=9606 GN=HP PE=1 SV=1
P12273	103.21	49	4.70E+05	6 (6)	Carbamidomethylation	16572	Prolactin-inducible protein OS=Homo sapiens OX=9606 GN=PIP PE=1 SV=1
Q08188	94.43	12	1.25E+05	6 (6)	Carbamidomethylation	76632	Protein-glutamine gamma-glutamyltransferase E OS=Homo sapiens OX=9606 GN=TGM3 PE=1 SV=4
Q6TEQ7	91.81	13	1.01E+05	4 (3)		38654	Annexin A2 OS=Canis lupus familiaris OX=9615 GN=ANXA2 PE=1 SV=1
Q5R5A0	91.81	13	1.01E+05	4 (3)		38604	Annexin A2 OS=Pongo abelii OX=9601 GN=ANXA2 PE=2 SV=1
P07355	91.81	13	1.01E+05	4 (3)		38604	Annexin A2 OS=Homo sapiens OX=9606 GN=ANXA2 PE=1 SV=2
P07356	91.81	13	1.01E+05	4 (3)		38676	Annexin A2 OS=Mus musculus OX=10090 GN=Anxa2 PE=1 SV=2
A6NMY6	91.81	13	1.01E+05	4 (3)		38659	Putative annexin A2-like protein OS=Homo sapiens OX=9606 GN=ANXA2P2 PE=5 SV=2
C0HJG9	91.81	22	1.01E+05	4 (3)		22447	Annexin A2 (Fragments) OS=Mesocricetus auratus OX=10036 PE=1 SV=1

Accession	-10lgP	Coverage (%)	Area	#Peptides (#unique)	PTM	Avg. Mass	Description
P31944	90.51	22	1.27E+05	5 (5)		27680	Caspase-14 OS=Homo sapiens OX=9606 GN=CASP14 PE=1 SV=2
P91754	87.31	10	8.30E+04	3 (3)		41323	Actin (Fragment) OS=Lumbricus rubellus OX=35632 PE=2 SV=1
P10365	87.31	10	8.30E+04	3 (3)		41635	Actin OS=Thermomyces lanuginosus OX=5541 PE=3 SV=1
P50138	87.31	10	8.30E+04	3 (3)		41745	Actin OS=Puccinia graminis OX=5297 PE=3 SV=1
P49871	87.31	10	8.30E+04	3 (3)		41777	Actin muscle OS=Manduca sexta OX=7130 PE=2 SV=1
P17126	87.31	10	8.30E+04	3 (3)		41797	Actin non-muscle 6.2 OS=Hydra vulgaris OX=6087 PE=3 SV=1
O16808	87.31	10	8.30E+04	3 (3)		41817	Actin OS=Mayetiola destructor OX=39758 PE=2 SV=1
P68555	87.31	10	8.30E+04	3 (3)		41745	Actin OS=Taenia solium OX=6204 GN=ACT1 PE=3 SV=1
Q26065	87.31	10	8.30E+04	3 (3)		41762	Actin adductor muscle OS=Placopecten magellanicus OX=6577 PE=2 SV=1
O17320	87.31	10	8.30E+04	3 (3)		41792	Actin OS=Crassostrea gigas OX=29159 PE=2 SV=1
P06702	84.85	51	2.12E+05	5 (5)	Oxidation (M)	13242	Protein S100-A9 OS=Homo sapiens OX=9606 GN=S100A9 PE=1 SV=1
Q9NZT1	84.04	21	6.45E+05	2 (2)		15893	Calmodulin-like protein 5 OS=Homo sapiens OX=9606 GN=CALML5 PE=1 SV=2
P04280	83.84	23	3.98E+04	7 (5)		38562	Basic salivary proline-rich protein 1 OS=Homo sapiens OX=9606 GN=PRB1 PE=1 SV=3
P01040	83.64	41	2.94E+05	4 (4)		11006	Cystatin-A OS=Homo sapiens OX=9606 GN=CSTA PE=1 SV=1
P22528	75.42	43	3.93E+05	4 (4)	Carbamidomethylation	9888	Cornifin-B OS=Homo sapiens OX=9606 GN=SPRR1B PE=1 SV=2
P02810	69.93	29	3.04E+05	4 (4)		17016	Salivary acidic proline-rich phosphoprotein 1/2 OS=Homo sapiens OX=9606 GN=PRH1 PE=1 SV=2
P25311	68.58	10	1.10E+05	3 (3)		34259	Zinc-alpha-2-glycoprotein OS=Homo sapiens OX=9606 GN=AZGP1 PE=1 SV=2
P31025	67.37	16	2.41E+05	3 (3)		19250	Lipocalin-1 OS=Homo sapiens OX=9606 GN=LCN1 PE=1 SV=1
Q5VSP4	67.37	18	2.41E+05	3 (3)		17918	Putative lipocalin 1-like protein 1 OS=Homo sapiens OX=9606 GN=LCN1P1 PE=5 SV=1
Q9BYE4	64.89	30	2.23E+05	2 (2)	Carbamidomethylation	8158	Small proline-rich protein 2G OS=Homo sapiens OX=9606 GN=SPRR2G PE=3 SV=1
P22531	64.89	31	2.23E+05	2 (2)	Carbamidomethylation	7855	Small proline-rich protein 2E OS=Homo sapiens OX=9606 GN=SPRR2E PE=2 SV=2

Accession	-10lgP	Coverage (%)	Area	#Peptides (#unique)	PTM	Avg. Mass	Description
P22532	64.89	31	2.23E+05	2 (2)	Carbamidomethylation	7905	Small proline-rich protein 2D OS=Homo sapiens OX=9606 GN=SPRR2D PE=2 SV=2
P35326	64.89	31	2.23E+05	2 (2)	Carbamidomethylation	7965	Small proline-rich protein 2A OS=Homo sapiens OX=9606 GN=SPRR2A PE=1 SV=1
P35325	64.89	31	2.23E+05	2 (2)	Carbamidomethylation	7975	Small proline-rich protein 2B OS=Homo sapiens OX=9606 GN=SPRR2B PE=2 SV=1
P05109	64.79	24	9.95E+04	2 (2)	Carbamidomethylation	10835	Protein S100-A8 OS=Homo sapiens OX=9606 GN=S100A8 PE=1 SV=1
Q96FQ6	60.25	22	6.48E+04	2 (2)		11801	Protein S100-A16 OS=Homo sapiens OX=9606 GN=S100A16 PE=1 SV=1
Q6B411	60.13	12	3.40E+05	2 (2)		16783	Lysozyme C milk isozyme OS=Bos taurus OX=9913 PE=2 SV=1
O75223	60.11	12	6.74E+04	2 (2)		21008	Gamma-glutamylcyclotransferase OS=Homo sapiens OX=9606 GN=GGCT PE=1 SV=1
P0DJD2	56.05	12	5.45E+04	2 (2)		10516	Apolipoprotein A-II OS=Pongo abelii OX=9601 GN=APOA2 PE=1 SV=1
P35325	64.89	31	2.23E+05	2 (2)	Carbamidomethylation	7975	Small proline-rich protein 2B OS=Homo sapiens OX=9606 GN=SPRR2B PE=2 SV=1
P05109	64.79	24	9.95E+04	2 (2)	Carbamidomethylation	10835	Protein S100-A8 OS=Homo sapiens OX=9606 GN=S100A8 PE=1 SV=1
Q96FQ6	60.25	22	6.48E+04	2 (2)		11801	Protein S100-A16 OS=Homo sapiens OX=9606 GN=S100A16 PE=1 SV=1
Q6B411	60.13	12	3.40E+05	2 (2)		16783	Lysozyme C milk isozyme OS=Bos taurus OX=9913 PE=2 SV=1
O75223	60.11	12	6.74E+04	2 (2)		21008	Gamma-glutamylcyclotransferase OS=Homo sapiens OX=9606 GN=GGCT PE=1 SV=1
P0DJD2	56.05	12	5.45E+04	2 (2)		10516	Apolipoprotein A-II OS=Pongo abelii OX=9601 GN=APOA2 PE=1 SV=1

(only matches with at least 2 unique peptides and 10% coverage are listed)

Table S 22 - Protein matches obtained for Honeycomb Italy Aged against Uniprot Honey Database

Accession	-10lgP	Coverage (%)	Area	#Peptides (#unique)	PTM	Avg. Mass	Description
O18330	158.74	29	1.56E+06	11 (11)	Oxidation (M)	48886	Major royal jelly protein 1 OS=Apis mellifera OX=7460 GN=MRJP1 PE=1 SV=1
A0A2A3EM69	98.71	10	8.30E+04	3 (3)		41806	Actin-5C OS=Apis cerana cerana OX=94128 GN=APICC_05085 PE=3 SV=1
A0A1V9XF46	98.71	10	8.30E+04	3 (3)		41839	Actin-5C-like OS=Tropilaelaps mercedesae OX=418985 GN=BIW11_10578 PE=3 SV=1
A0A2A3E2E7	98.71	9	8.30E+04	3 (3)		45603	Actin clone OS=Apis cerana cerana OX=94128 GN=APICC_04920 PE=3 SV=1
A0A2A3E8T5	98.71	9	8.30E+04	3 (3)		45827	Actin OS=Apis cerana cerana OX=94128 GN=APICC_08334 PE=3 SV=1
Q17060	98.45	8	1.04E+05	3 (2)	Carbamidomethylation; Deamidation (NQ); Oxidation (M)	61662	Major royal jelly protein 3 OS=Apis mellifera OX=7460 GN=MRJP3 PE=1 SV=1
O77061	88.99	8	1.14E+05	3 (2)		51074	Major royal jelly protein 2 OS=Apis mellifera OX=7460 GN=MRJP2 PE=1 SV=1
A0A1V9XCK9	57.66	35	3.35E+04	6 (6)	Deamidation (NQ)	9408	UPF0480 protein C15orf24-like OS=Tropilaelaps mercedesae OX=418985 GN=BIW11_11081 PE=4 SV=1
A0A2A3ECX5	41.73	11	6.43E+04	2 (2)	Carbamidomethylation	29815	Trypsin-2 OS=Apis cerana cerana OX=94128 GN=APICC_08270 PE=4 SV=1
A0A1S2VRR0	39.04	8	4.63E+03	2 (1)	Deamidation (NQ); Oxidation (M)	73158	Non-specific serine/threonine protein kinase OS=Bifidobacterium longum subsp. infantis OX=1682 GN=BFS25_10435 PE=4 SV=1
A0A1S2VX47	28.40	9	2.70E+06	1 (1)		12737	Uncharacterized protein OS=Bifidobacterium longum subsp. infantis OX=1682 GN=BFS25_05850 PE=4 SV=1
V6XYG9	28.40	9	2.70E+06	1 (1)		12738	Uncharacterized protein OS=Bifidobacterium longum E18 OX=1322347 GN=BLONG_1920 PE=4 SV=1
A0A4R0USA9	28.40	9	2.70E+06	1 (1)		12738	Uncharacterized protein OS=Bifidobacterium longum subsp. longum OX=1679 GN=MCC10102_1887 PE=4 SV=1
A0A6H9Y183	24.01	13	3.66E+04	1 (1)		8135	GNAT family N-acetyltransferase OS=Bifidobacterium longum subsp. infantis OX=1682 GN=F8277_04630 PE=4 SV=1
A0A1V9XJD9	21.77	10	7.43E+04	1 (1)		11001	Uncharacterized protein OS=Tropilaelaps mercedesae OX=418985 GN=BIW11_03547 PE=3 SV=1
B8XIJ0	20.30	9	7.92E+05	1 (1)		30507	Tweedle motif cuticular protein 1 (Fragment) OS=Apis mellifera OX=7460 GN=Twld1 PE=2 SV=1

(only matches with at least 5% coverage and 1 unique peptide are listed)

Table S 23 - Peptides recovered from the residue and matched to major royal jelly protein 1 *Apis mellifera,* taxonomically informative residue and amino acid variants highlighted.

Peptide	-10lgP	Avg. Mass	Intensity	PTM	Unique (Yes/No)
R.TSDYQQNDIHYEGVQNILDTQSSAK.V	64.42	2853	3.11E+05		Yes
K.M(+15.99)VNNDFNFDDVNFR.I	63.46	1762	1.47E+05	Oxidation (M)	Yes
K.LLTFDLTTSQLLK.Q	51.91	1492	2.44E+04		Yes
**R.IM(+15.99)NANVNELILNTR.C**	**49.80**	**1630**	**1.96E+05**	**Oxidation (M)**	**Yes**
K.YDDC(+57.02)SGIM(sub V)SASK.L	47.89	1333	4.73E+04	Carbamidomethylation	Yes
K.FFDYDFGSDER.R	38.91	1397	2.82E+04		Yes
L.YYVNTEQFR.T	24.05	1219	4.15E+04		Yes

Table S 24 - The analytical journey of the Paestum's residue

Report / Year	Sample Context	Analytical Methods	Main Findings	Interpretation / Conclusion
Field Archaeologists (**1952**)	Residue found in the bottom of bronze vessels originally sealed with cork stoppers; liquid traces on vessels' exterior.	Visual observation, contextual inference	Residue appears to be the same in all vessels. It has a waxy consistency, with a smell suggestive of beeswax.	Interpreted as an offering of honey as honeycomb, with beeswax being the surviving component.
Bienenabteilung, Liebefeld–Bern, via London's Bee Research Association (**1957**)	Sample origin unspecified.	Solubility tests; microscopic analysis of insoluble fraction	Insoluble in water; Waxy in character. Sediment contained fungal spores, hyphae, scarce pollen grains, insect hairs, wood & plant fragments.	Pollen profile inconsistent with honey. Sample provided hypothesized to have been of a wax seal used to close the vessel.
Central Institute of Restoration, Rome (**1970**)	Two samples taken from Amphora 4: near the neck and from the base/bottom.	Solubility tests, fusion test, chloroform extraction followed by hydrolysis and separation of fractions	Saponifiable substances detected; no sugars, fats, proteins or glycerine. Fatty acids and alcohols present; base sample richer in organics. Fusion point:130 °C.	Likely a heterogeneous mixture of wax-like substances. Suggested degradation.
Chamber of Commerce Laboratory, Rome (**1983**)	Sample origin unspecified.	Solubility tests; GC-MS	Residue insoluble in water but highly soluble in ethyl ether; 99% saponifiable. Major fatty acids: palmitic acid (77.4%), stearic (5.2%), oleic (6.1%), and heptadecanoic (1.0%). No glycerine or sugars detected.	Identified as degraded triglycerides from animal or vegetable fat origin. Absence of glycerine attributed to long-term transformation. Not diagnostic of honey.
Current study (**2025**)	Samples (core and surface) taken from a residue mass that arrived at the Ashmolean Museum in 2019 for display.	FTIR-ATR, TSP-GC/MS, AEC-MS (water extract), Bottom-up Proteomics, XPS	Core sample similar to modern beeswax, except by high acid content. Aldehydes, mid and long-chain fatty acids and an ester detected in core sample; sugar derivatives detected on the black surface area alongside Cu^{+1} ions. Intact hexose sugars and high abundance of taurine detected in core sample, together with Major royal jelly proteins from Apis mellifera (akin to those found in modern honeycombs)	Residue contained a bee product, likely to have been honey originally offered as honeycomb. Current chemical complexity due to microbial action.
